# A macrophage-targeted TIMD4 Peptide-Nanoparticle attenuates colitis symptoms and monocyte recruitment in a mouse model

**DOI:** 10.1371/journal.pbio.3004056

**Published:** 2026-10-21

**Authors:** Huimin Liu, Yuzhen Wang, Ruiqing Yu, Kexin Liu, Dezhi Zheng, Bo Wang, Yuan Chang, Bing Liu, Xuetian Yue, Zhuanchang Wu, Chunyang Li, Xiaohong Liang, Chunhong Ma, Guohua Liu, Jianhua Li, Leiqi Xu, Lifen Gao

**Affiliations:** 1 Key Laboratory for Experimental Teratology of Ministry of Education, Key Laboratory of Infection Immunity and Disease Intervention of Shandong Province, and Department of Immunology, School of Basic Medical Sciences, Cheeloo College of Medicine, Shandong University, Jinan, Shandong, P. R. China; 2 Department of Biomaterials, School and Hospital of Stomatology, Cheeloo College of Medicine, Shandong University & Shandong Key Laboratory of Oral Tissue Regeneration & Shandong Engineering Research Center of Dental Materials and Oral Tissue Regeneration & Shandong Provincial Clinical Research Center for Oral Diseases, Jinan, Shandong, P. R. China; 3 The First School of Clinical Medicine, Shandong University, Jinan, Shandong, P. R. China; 4 Department of Oral Medicine, Jining Medical University, Jining, Shandong, P. R. China; 5 Department of Rheumatology, Fuding Hospital, Ningde City, Fujian Province, P. R. China; 6 Key Laboratory for Experimental Teratology of Ministry of Education, Department of Cell Biology, School of Basic Medical Sciences, Cheeloo College of Medicine, Shandong University, Jinan, Shandong, P. R. China; 7 Key Laboratory for Experimental Teratology of Ministry of Education, Department of Histology and Embryology, School of Basic Medical Sciences, Cheeloo College of Medicine, Shandong University, Jinan, Shandong, P. R. China; 8 Institute of Pediatric Research, Children’s Hospital Affiliated to Shandong University, Jinan, Shandong, P. R. China; 9 Department of Gastroenterology, Qilu Hospital, Shandong University, Jinan, Shandong, P. R. China; 10 Shandong Key Laboratory of Oral Diseases & Shandong Engineering Research Center of Dental Materials and Oral Tissue Regeneration & Shandong Provincial Clinical Research Center for Oral Diseases, School and Hospital of Stomatology, Cheeloo College of Medicine, Shandong University, Jinan, Shandong, P. R. China; University of Pennsylvania, UNITED STATES OF AMERICA

## Abstract

Macrophages play a pivotal role in the pathogenesis of inflammatory bowel disease (IBD). T-cell immunoglobulin and mucin domain-containing molecule 4 (TIMD4) is involved in the regulation of tissue-resident macrophages. TIMD4 expression is elevated in colonic tissues from patients with ulcerative colitis (UC) and mice with dextran sulfate sodium (DSS)-induced colitis, but its role in IBD pathogenesis remains unclear. In this study, we found that *Timd4* deficiency aggravated DSS-induced colitis, accompanied by increased infiltration of monocytes into the colonic tissues. Treatment with a CCR2 antagonist attenuated colonic inflammation and reduced monocyte infiltration into colonic tissues in *Timd4* KO mice. Mechanistically, in vitro studies revealed that TIMD4 stabilized the LKB1-STRAD-MO25 complex via its intracellular domain, thereby activating the AMPK pathway and suppressing NF-κB-dependent chemokine production in macrophages. Based on these findings, we designed a TIMD4-derived peptide and developed a macrophage-targeted nanoparticle system (T4_(365–378)_‑NPs). Treatment with T4_(365–378)_-NPs reduced P65 phosphorylation and attenuated DSS-induced colitis in mice. Treatment with either T4_(365–378)_-NPs or the CCR2 antagonist attenuated DSS-induced colitis, and no statistically significant differences in the assessed outcomes were detected between the two groups. Administration of T4_(365–378)_-NPs before DSS exposure also attenuated subsequent colitis. Furthermore, combining the T4_(365–378)_‑NPs with anti-α4β7 or anti-TNF-α antibodies produced greater improvements than either monotherapy in the DSS-induced colitis model. These findings support further preclinical evaluation of TIMD4-derived peptide delivery as a potential therapeutic approach for intestinal inflammation.

## Abstract 2

Inflammatory bowel disease is fueled by macrophage signals that recruit monocytes to the gut. This study shows that TIMD4 activates the AMPK pathway and suppresses NF-κB-dependent chemokine production in macrophages, and that a TIMD4 peptide nanoparticle alleviates these effects.

## Introduction

Inflammatory bowel disease (IBD) denotes chronic inflammatory conditions of the gastrointestinal tract, primarily encompassing Crohn’s disease (CD) and ulcerative colitis (UC). IBD arises from a complex interplay between genetic susceptibility and environmental triggers [1]. The core pathogenesis resides in the disruption of intestinal immune homeostasis, characterized by persistent aberrant activation of mucosal immune cells (particularly macrophages and T-cell subsets) driving a self-perpetuating cycle of chronic inflammation and progressive tissue damage [2–4]. Macrophages play a dual role yet context-dependent role in the pathogenesis, resolution of intestinal inflammation and tissue repair with IBD. Consequently, therapeutic strategies aimed at selectively modulating macrophage phenotype and function offer considerable translational potential. Thus, elucidating the mechanisms that regulate macrophage function in IBD and developing strategies to modulate macrophage inflammatory responses may provide new opportunities to limit intestinal inflammation, promote mucosal repair and restore colonic homeostasis.

Monocytes and macrophages exhibit substantial heterogeneity and functional plasticity [5]. Under physiological conditions, Ly6C^+^ monocytes undergo terminal differentiation into CX3CR1^hi^F4/80^+^ resident macrophages, which safeguard colonic homeostasis through immunomodulatory and tissue-protective functions [6]. However, during intestinal inflammation, signals derived from damage-associated molecular patterns or pathogen-associated molecular patterns recruit abundant Ly6C^+^ monocytes but block their terminal differentiation, leading to aberrant accumulation of intermediate Ly6C^int^CX3CR1^int^ macrophages [7]. These cells not only serve as primary sources of pro-inflammatory cytokines (IL-12, IL-23, IL-1β) [6,8], but also drive Th1/Th17 immune responses, exacerbating inflammation [9,10], which might contribute to the sustained deterioration of colonic inflammation and tissue damage. Thus, reducing the accumulation of pro-inflammatory intermediate macrophages may help limit intestinal inflammation. Consistently, CCR2 deficiency has been shown to inhibit the accumulation of immature macrophages in IL10R-deficient mice [11], and administration of anti-CCR2 antibodies has been found to ameliorate colonic inflammation in murine models [12]. In addition, macrophages might be involved in driving monocyte recruitment by secreting the chemokines CCL2, CCL7 and CCL8 [12]. Macrophages also contribute to the resolution of inflammation, with IL-10 produced by CX3CR1^hi^ macrophages supporting intestinal homeostasis and tissue repair [6,13]. Therefore, regulation of monocyte recruitment and macrophage differentiation is important for maintaining intestinal homeostasis. Macrophage trafficking is regulated by chemokines and their receptors. Nuclear factor κB (NF-κB) acts as a central hub controlling the expression of a broad range of chemokines [14], thereby governing monocyte recruitment and macrophage accumulation in the inflamed intestine [15]. Accumulating evidence indicates that AMP-activated protein kinase (AMPK) can potently suppress NF-κB signaling through multiple mechanisms [16,17]. In a short-term fasting model, activation of AMPK in hepatocytes and PPARα-mediated suppression of systemic CCL2 production reduced monocyte mobilization from the bone marrow, resulting in fewer circulating monocytes [18]. AMPK activation is primarily mediated by upstream kinases, among which the liver kinase B1 (LKB1) serves as a major activating kinase [19]. Targeting the LKB1-AMPK-NF-κB regulatory axis may therefore provide a potential macrophage-directed therapeutic strategy for IBD.

T-cell immunoglobulin and mucin domain-containing protein 4 (TIMD4), an immune checkpoint molecule predominantly expressed on macrophages and dendritic cells, plays a pivotal role in regulating macrophage homeostasis and functional activity through multiple mechanisms [20,21]. TIMD4 contributes to apoptotic cell clearance by recognizing phosphatidylserine and has been associated with anti-inflammatory macrophage responses, including reduced production of TNF-α and IL-6 and increased production of IL-10 and TGF-β, which may support local immune tolerance [22–24]. In addition, TIMD4 has been reported to attenuate LPS-induced endotoxic shock by suppressing NF-κB-dependent cytokine production in macrophages. In obesity-related models, TIMD4 supported adipose tissue homeostasis by promoting M2-like macrophage polarization through suppression of NF-κB signaling [22,25]. Given these fundamental immunoregulatory functions, a close association between TIMD4 and IBD has been increasingly recognized. Genetic analyses have implicated TIMD4 as a candidate gene associated with IBD susceptibility, with evidence suggesting an association between higher TIMD4 expression and increased CD risk [26]. Consistently, it has been reported that TIMD4 expression is significantly upregulated in both UC patients and murine colitis models, and is positively correlated with disease activity and severity [27,28], further supporting its potential role as a key regulator in intestinal inflammation. However, the mechanisms through which TIMD4 influences intestinal inflammation and its potential therapeutic relevance remain incompletely understood.

Here, we found that *Timd4* deficiency aggravated DSS-induced colitis in mice, accompanied by increased colonic monocyte infiltration. Mechanistically, in vitro experiments supported a role for the intracellular domain of TIMD4 in stabilizing the LKB1-STRAD-MO25 complex and regulating chemokine expression through LKB1/AMPK/NF-κB signaling. Based on these findings, we developed a macrophage-targeted TIMD4-derived peptide delivery system that attenuated DSS-induced colitis in mice. Its combination with anti-α4β7 or anti-TNF-α antibodies produced greater improvements than the corresponding antibody monotherapy. Together, these findings support a role for TIMD4 in regulating intestinal inflammation and provide a preclinical rationale for exploring TIMD4-derived peptide delivery as a therapeutic approach.

## Results

### *Timd4* deficiency exacerbates DSS-induced colitis in mice

Immunofluorescence staining showed markedly increased TIMD4 fluorescence intensity and a greater number of TIMD4-positive cells in the colonic mucosa of UC patients compared to healthy controls (Fig 1A and 1B). Consistently, Western blotting analysis of colonic biopsy tissues showed elevated TIMD4 protein levels in UC patients (Fig 1C and 1D). Analysis of the Gene Expression Omnibus (GEO) database also showed elevated *TIMD4* expression in inflamed colonic tissues of UC patients compared to healthy controls (Fig 1E). Furthermore, the proportion of *TIMD4*-positive cells in the colonic mucosa of colitis patients was significantly higher than that in healthy controls (Fig 1F). Moreover, after 52 weeks of treatment, *TIMD4* expression in the colonic tissues of these patients was markedly reduced (Fig 1G). In mice with acute colitis induced by 3% DSS (S1 Fig), Western blotting and immunohistochemical analyses demonstrated significantly higher TIMD4 protein levels in colonic tissues of DSS-treated mice compared with those in the control group (Fig 1H–1K).

**Fig 1:**
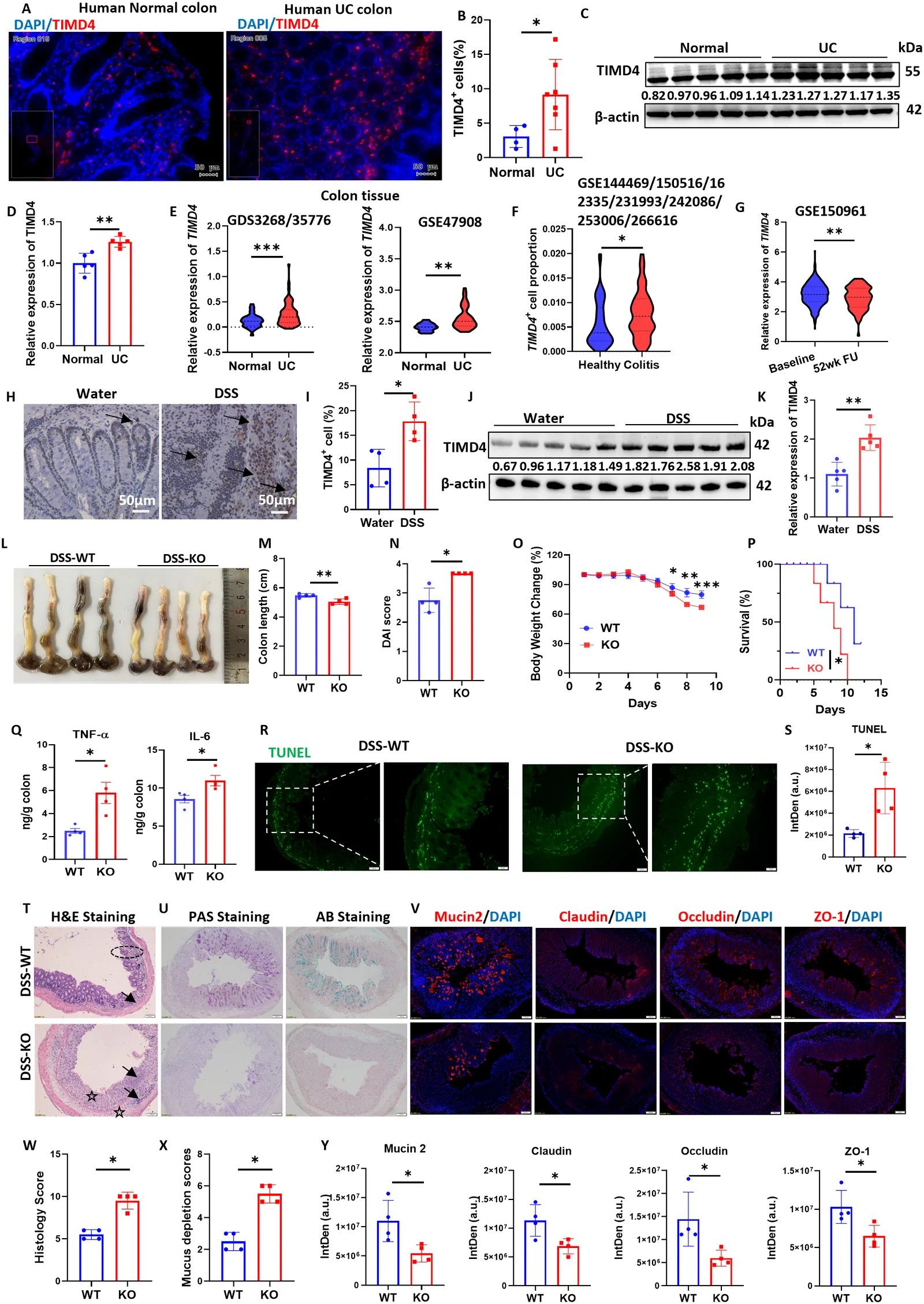
*Timd4* deficiency aggravates DSS-induced colitis in mice. **(A, B)** Immunofluorescence staining images of colon biopsy specimens from UC patients and healthy controls (normal: *n* = 4, UC: *n* = 7; scale bar: 20 μm). Statistical analysis of TIMD4 protein expression in immunofluorescence image. **(C, D)** Western blotting image and statistical analysis of the relative TIMD4 expression in human colonic biopsy tissues (*n* = 5). **(E)** The data from GEO database of GDS3268/35776 (Normal colon: *n* = 69, UC inflamed colon: *n* = 63) and GSE47908 (Normal colon: *n* = 15, UC inflamed colon: *n* = 45) were collected to compare *TIMD4* mRNA expression level between colonic tissues from UC patients and normal colonic tissues. **(F)** Validation of *TIMD4* expression changes at the single-cell level. Additional analyses of multiple publicly available single-cell RNA-seq datasets (GSE150516, GSE144469, GSE162335, GSE231993, GSE242086, GSE253006 and GSE266616) were performed (Healthy: *n* = 59, Colitis: *n* = 47). **(G)** Analysis of *TIMD4* expression in an RNA-seq dataset (GSE150961). Human colonic tissue samples were collected before treatment and after 52 weeks of therapeutic follow-up (Baseline: *n* = 331, 52wk FU: *n* = 87). **(H)** Representative immunohistochemical staining images of colon specimens obtained from DSS-induced colitis mice and controls, and the arrows indicate TIMD4^+^ cells. Scale bar: 50 μm. **(I)** Quantitative analysis of TIMD4^+^ cells in colonic tissue (*n* = 4). **(J, K)** Western blotting image and statistical analysis of the relative TIMD4 expression in colonic tissues from control mice or DSS-induced colitis mice (*n* = 5). **(L, M)** Gross morphology of mouse colon and colon length measurements recorded on day 9 (*n* = 4). **(N)** Body weight, stool consistency and rectal bleeding were scored and summated to calculate the DAI scores. **(O)** Body weight changes were assessed daily (*n* = 4). Statistics on the rate of weight loss on the days 7–9. **(P)** WT and *Timd4* KO mice were administered 4% DSS to induce acute colitis. Survival of mice was monitored until day 12 (*n* = 8). **(Q)** Colonic tissues were homogenized, and cytokines (IL-6, TNF-α) in the supernatants were measured by ELISA (*n* = 4). **(R, S)** Apoptotic cells were detected by TUNEL staining, and TUNEL fluorescence intensity was quantified (*n* = 4). **(T)** Representative H&E staining images of colonic tissues. → Inflammation, ☆ Edema, the black dotted lines highlight the crypt. **(U)** Representative images of PAS staining and AB staining in colonic sections. **(V)** Immunofluorescence images of colon sections with Mucin2, Claudin, Occludin and ZO-1 (Red). Scale bars: 100 μm. **(W)** Statistical analysis of histopathological score (*n* = 4). **(X)** Quantification of mucus depletion scores based on PAS staining and AB staining (*n* = 4). **(Y)** Statistics on immunofluorescence intensity of Mucin2, Claudin, Occludin and ZO-1 (*n* = 4). All data are presented as mean ± SEM. The unpaired Student’s *t* test was employed to assess differences between two groups. Survival curves were compared using the log-rank (Mantel–Cox) test. Statistical significance was reported as * *p* < 0.05, ** *p* < 0.01, *** *p* < 0.001. The data underlying this figure can be found in S1 Data.

To investigate the role of TIMD4 in DSS-induced colitis, WT and *Timd4* KO mice were subjected to 3% DSS treatment. Compared with WT mice, *Timd4* KO mice exhibited exacerbated pathological phenotypes, characterized by pronounced colon shortening (Fig 1L and 1M), elevated disease activity index (DAI) scores (Fig 1N) and accelerated weight loss (Fig 1O). In a separate experiment using 4% DSS, *Timd4* KO mice also showed reduced survival (Fig 1P). Colonic tissues from *Timd4* KO mice exhibited higher IL-6 and TNF-α levels (Fig 1Q) and an increased number of apoptotic cells (Fig 1R and 1S). Histopathological evaluation revealed more severe colonic damage in *Timd4* KO mice, accompanied by inflammatory cell infiltration in the lamina propria and submucosa, epithelial denudation and crypt atrophy (Fig 1T and 1W). PAS and AB staining showed higher mucus damage scores in *Timd4* KO mice (Fig 1U and 1X), together with reduced Mucin2 fluorescence intensity (Fig 1V and 1Y). In addition, the fluorescence intensities of the tight junction proteins Claudin, Occludin and ZO-1 were higher in WT mice than in *Timd4* KO mice (Fig 1V and 1Y). More severe colonic inflammation was also observed in female *Timd4* KO mice exposed to 2% DSS (S2 Fig) and in male *Timd4* KO mice exposed to 3% DSS (S3 Fig). Under steady-state conditions, no significant differences were detected between WT and *Timd4* KO mice in the parameters examined (S4 Fig). Together, these findings indicate that *Timd4* deficiency increases susceptibility to DSS-induced colitis, accompanied by greater mucosal injury and alterations in intestinal barrier-associated markers.

### Macrophages contribute to TIMD4-mediated protection against DSS-induced colitis

To further investigate the protective role of TIMD4 in DSS-induced colitis, we examined its expression in different immune cell populations in colonic tissues. Immunofluorescence staining revealed co-localization of CD68 and TIMD4 (Fig 2A). Immunofluorescence staining and flow cytometry analysis revealed significant upregulation of TIMD4 expression in colonic macrophages from mice with DSS-induced colitis (Figs 2A–2C and S5). However, no significant changes in TIMD4 expression were detected in colonic DCs, T lymphocytes or B cells (S6 Fig). These observations prompted us to further examine the contribution of macrophages to the colitis phenotype associated with *Timd4* deficiency.

**Fig 2:**
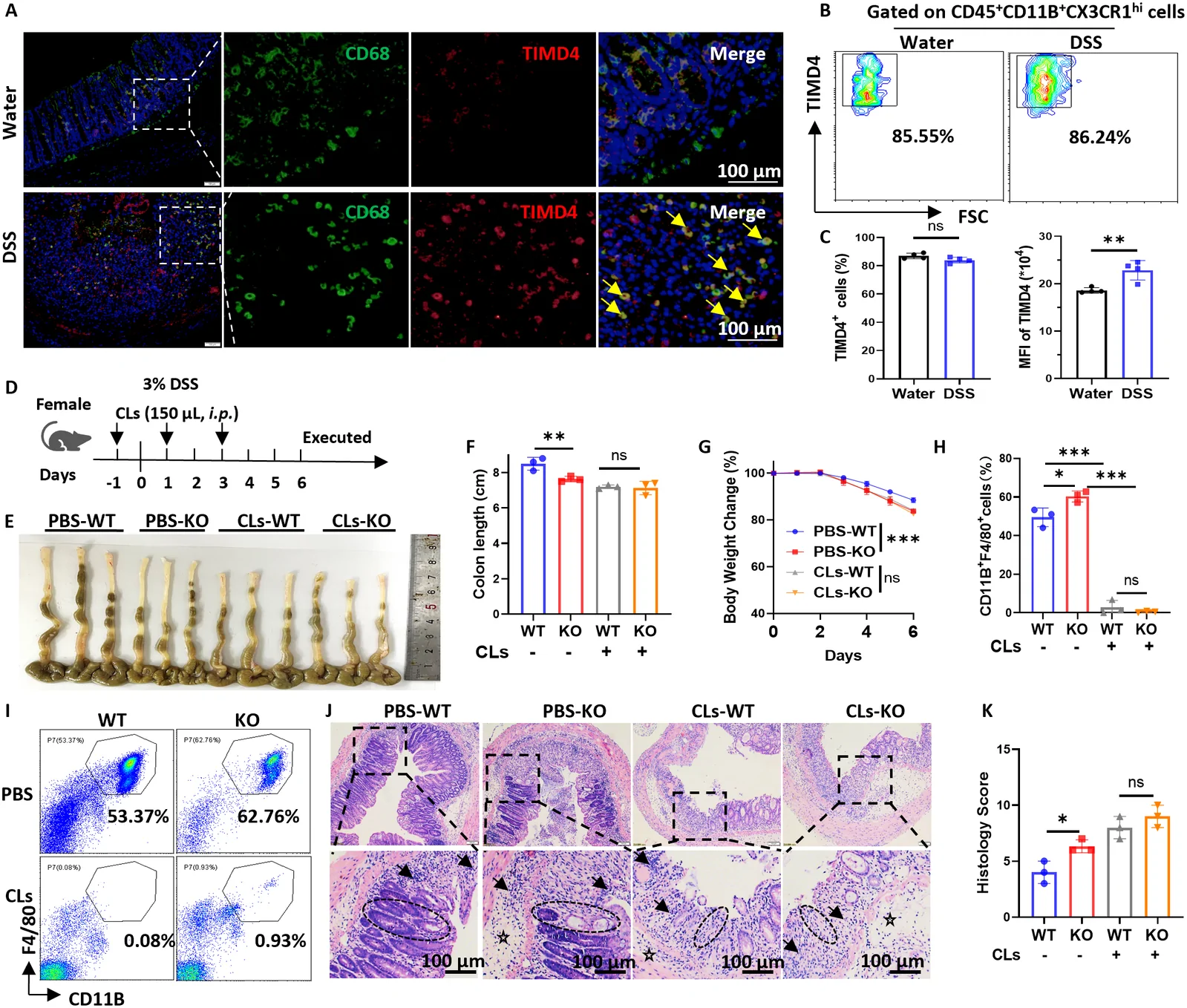
Macrophages contribute to the protective effects of TIMD4 during DSS-induced colitis. **(A)** Immunofluorescence images of colonic tissues from control mice and DSS-induced colitis mice, with TIMD4 (red), CD68 (green), and DAPI for nuclei (blue). Scale bars: 100 μm. Yellow arrows indicate CD68^+^TIMD4^+^ cells. **(B, C)** Flow cytometry analysis of the proportion of TIMD4^+^ cells and the mean fluorescence intensity of TIMD4 on CD11B^+^CX3CR1^hi^ macrophages in colonic tissues from steady-state or colitis mice (*n* = 4). **(D)** Macrophages were depleted by CLs (liposome, C-005, 150 μL) in WT and *Timd4* KO mice subjected to 3% DSS treatment for 7 days, and the mice were sacrificed on day 7. **(E, F)** Gross morphology of mouse colon and colon length measurements recorded on day 7 after DSS treatment (*n* = 3). **(G)** Body weight changes were assessed daily (*n* = 3). Statistics on the rate of weight loss on day 6. **(H, I)** Flow cytometry analysis of the efficiency of macrophage depletion in steady-state peritoneal cavity by CLs treatment. **(J)** Representative H&E-stained images of colonic sections. → Inflammation, ☆ Edema, the black dotted lines highlight the crypt. Scale bars: 100 μm (whole colonic sections). **(K)** Statistical analysis of histopathological scores. All data are presented as mean ± SEM (*n* ≥ 3). The unpaired Student’s *t* test was employed to assess differences between two groups. One-way ANOVA was employed to assess differences between multiple groups. Statistical significance was reported as * *p* < 0.05, ** *p* < 0.01, *** *p* < 0.001 and ns, no significance. The data underlying this figure can be found in S1 Data.

To assess this contribution, WT and *Timd4* KO mice were treated with clodronate liposomes (CLs) during DSS-induced colitis (Fig 2D). Flow cytometric analysis confirmed macrophage depletion in the peritoneal cavity following CLs administration (Fig 2H and 2I). Macrophage depletion exacerbated colitis in both WT and *Timd4* KO mice, as reflected by greater colon shortening and higher histopathological scores. Following depletion, no statistically significant differences in colon length, body weight loss or histopathological scores were detected between WT and *Timd4* KO mice (Fig 2E–2G, 2J and 2K). Together with the increased TIMD4 expression in colonic macrophages following DSS treatment, these findings support the involvement of macrophages in TIMD4-associated protection against DSS-induced colitis.

### *Timd4*-deficient macrophages exhibit enhanced expression of monocyte-recruiting chemokines

To further explore the protective mechanism associated with TIMD4, we performed Gene Ontology (GO) enrichment analysis of differentially expressed genes between *TIMD4*^hi^ and *TIMD4*^low^ UC samples in the GSE16879 dataset. This analysis revealed significant enrichment in leukocyte migration and chemotaxis-associated pathways (Fig 3A). Complementary Kyoto Encyclopedia of Genes and Genomes (KEGG) pathway analysis and murine Gene Set Enrichment Analysis (GSEA) of RNA-seq data revealed marked enrichment of chemokine signaling pathways and increased expression of monocyte-associated chemokines in macrophages from *Timd4* KO mice (Figs 3B, 3C, and S7). Expression of *Ccl2, Ccl3, Ccl5, Ccl7* and *Ccl8* was higher in colonic tissues from DSS-treated *Timd4* KO than in those from WT mice (Fig 3D). Consistently, LPS-stimulated bone marrow-derived macrophages (BMDMs) and peritoneal macrophages (PEMs) from *Timd4* KO mice exhibited higher mRNA expression of these chemokines than their WT counterparts (Figs 3E and S8A).

**Fig 3:**
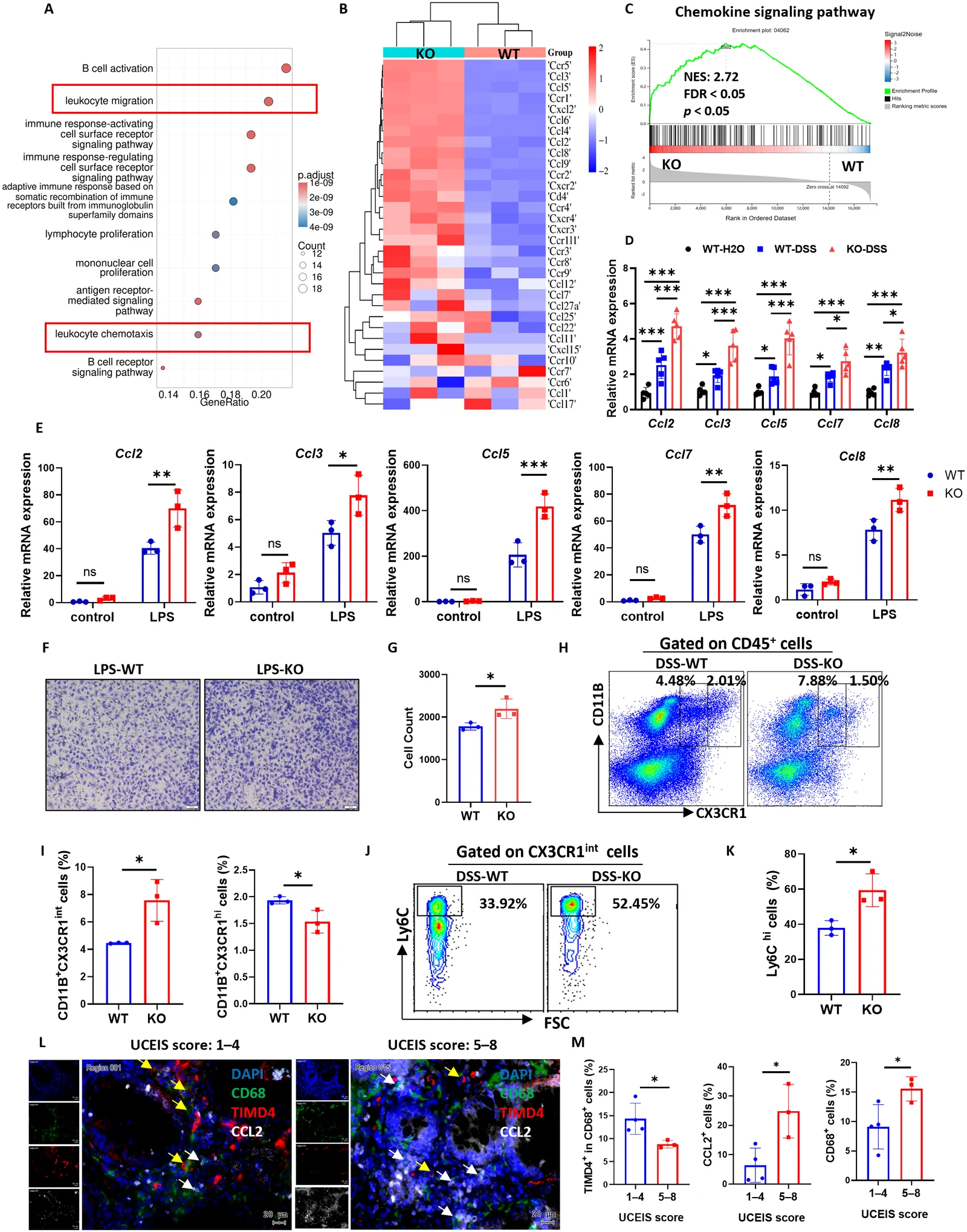
*Timd4*-deficient macrophages exhibit enhanced expression of monocyte-recruiting chemokines. **(A)** In the database GSE16879, 133 UC samples were divided into two groups according to the median of *TIMD4* expression levels: 67 cases in the *TIMD4*^hi^ group; 66 cases in the *TIMD4*^low^ group. Differential gene expression analysis was performed between the two groups using the *limma* package, and differential genes were selected for GO enrichment analysis (p < 0.05, |logFC| > 1). (B, C) Resident macrophages from WT and *Timd4* KO mice were sorted using F4/80 magnetic beads. These cells were collected and subjected to RNA sequencing. **(B)** Heatmap analysis of chemokine-related genes. **(C)** GSEA analysis of RNA-seq data. **(D)** RNA was extracted from colonic tissues of mice after DSS treatment. The expression of monocyte-recruiting chemokines in colonic tissues was detected by RT-qPCR. **(E)** RNA was extracted after BMDMs were stimulated for 24 h with LPS (100 ng/mL). The expression of monocyte-recruiting chemokines was detected by RT-qPCR. **(F)** Recruited macrophages were stained using crystal violet. Images were observed through microscopic examination (Olympus, Tokyo), Scale bars: 50 μm. **(G)** Statistical analysis on the numbers of crystal violet-stained cells. **(H)** Representative flow cytometric plots of the proportion of CD11b^+^CX3CR1^hi^ macrophages and CD11b^+^CX3CR1^int^ macrophages gated on CD45^+^ cells isolated from colonic tissues from WT and KO mice after DSS treatment. **(I)** Statistical analysis of flow cytometry results for CD11b^+^CX3CR1^hi^ and CD11b^+^CX3CR1^int^ subpopulations. **(J)** Representative flow cytometry plot of the proportion of Ly6C^hi^ cells gated on CD45^+^CD11b^+^CX3CR1^int^ cells. **(K)** Statistical analysis of flow cytometry results for CD45^+^CD11b^+^CX3CR1^int^Ly6C^hi^ cells. **(L)** Immunofluorescence image of colon biopsy specimens from UC patients with different UCEIS scores. Yellow arrows indicate CD68⁺ TIMD4⁺ cells, whereas white arrows indicate CCL2⁺ cells. **(M)** Statistical analysis of TIMD4 protein expression in CD68^+^ cells, proportion of CCL2^+^ cells and CD68^+^ cells (mild: *n* = 4, moderate and severe: *n* = 3). All data are presented as mean ± SEM (*n* ≥ 3). The unpaired Student’s *t* test was employed to assess differences between two groups. One-way ANOVA **(D)** and two-way ANOVA **(E)** were employed to assess differences between multiple groups. Statistical significance was reported as * *p* < 0.05, ** *p* < 0.01, *** *p* < 0.001 and ns, no significance. The data underlying this figure can be found in S1 Data.

We next examined whether *Timd4* deficiency affected macrophage-mediated cell recruitment. Transwell migration assays showed that *Timd4* KO macrophages promoted greater cell migration than WT macrophages (Fig 3F and 3G). In DSS-induced colitis, flow cytometry revealed a lower proportion of CD11b^+^CX3CR1^hi^ macrophages and higher proportions of CD11b^+^CX3CR1^int^ macrophages and Ly6C^hi^ monocytes in colonic tissues from *Timd4* KO mice than in those from WT mice (Fig 3H–3K). In human colonic biopsy specimens, patients with more severe UC exhibited lower TIMD4 expression in colonic macrophages, together with higher proportions of CCL2-positive and CD68-positive cells, compared with patients with mild UC (Fig 3L and 3M).

Furthermore, *Timd4* KO macrophages exhibited increased expression of additional inflammation-related chemokines, including *Cxcl1*, *Cxcl2*, *Cxcl3*, *Cxcl5*, *Cxcl9*, *Cxcl10*, *Cxcl11* and *Ccl20* (S8B Fig), suggesting that the effects of *Timd4* deficiency on chemokine expression extend beyond monocyte-associated chemokines. In addition, *Timd4* KO mice exhibited a lower proportion of CD206^+^ macrophages and reduced mRNA expression of anti-inflammatory and tissue repair-associated genes, including *Il10*, *Tgfb1* and *Arg1* in colonic tissues (S9 Fig).

Together, these findings suggest that TIMD4 contributes to the regulation of macrophage chemokine expression and cell recruitment, with *Timd4* deficiency accompanied by changes in colonic monocyte and macrophage populations during DSS-induced colitis.

### CCR2 antagonist ameliorates colitis exacerbated by *Timd4* deficiency

CCR2 is a key regulator of the directed migration of circulating monocytes. To evaluate the contribution of CCR2-dependent monocyte recruitment to the aggravated colitis associated with *Timd4* deficiency, the CCR2 antagonist RS102895 was administered to mice during DSS‑induced colitis (Fig 4A). In the absence of RS102895, *Timd4* KO mice exhibited greater body weight loss, higher DAI scores, more pronounced colon shortening and higher levels of IL-6, IL-1β and TNF-α in colonic tissues than those of WT mice. Following RS102895 treatment, no statistically significant differences in these parameters were observed between WT and *Timd4* KO mice (Fig 4B–4F).

**Fig 4:**
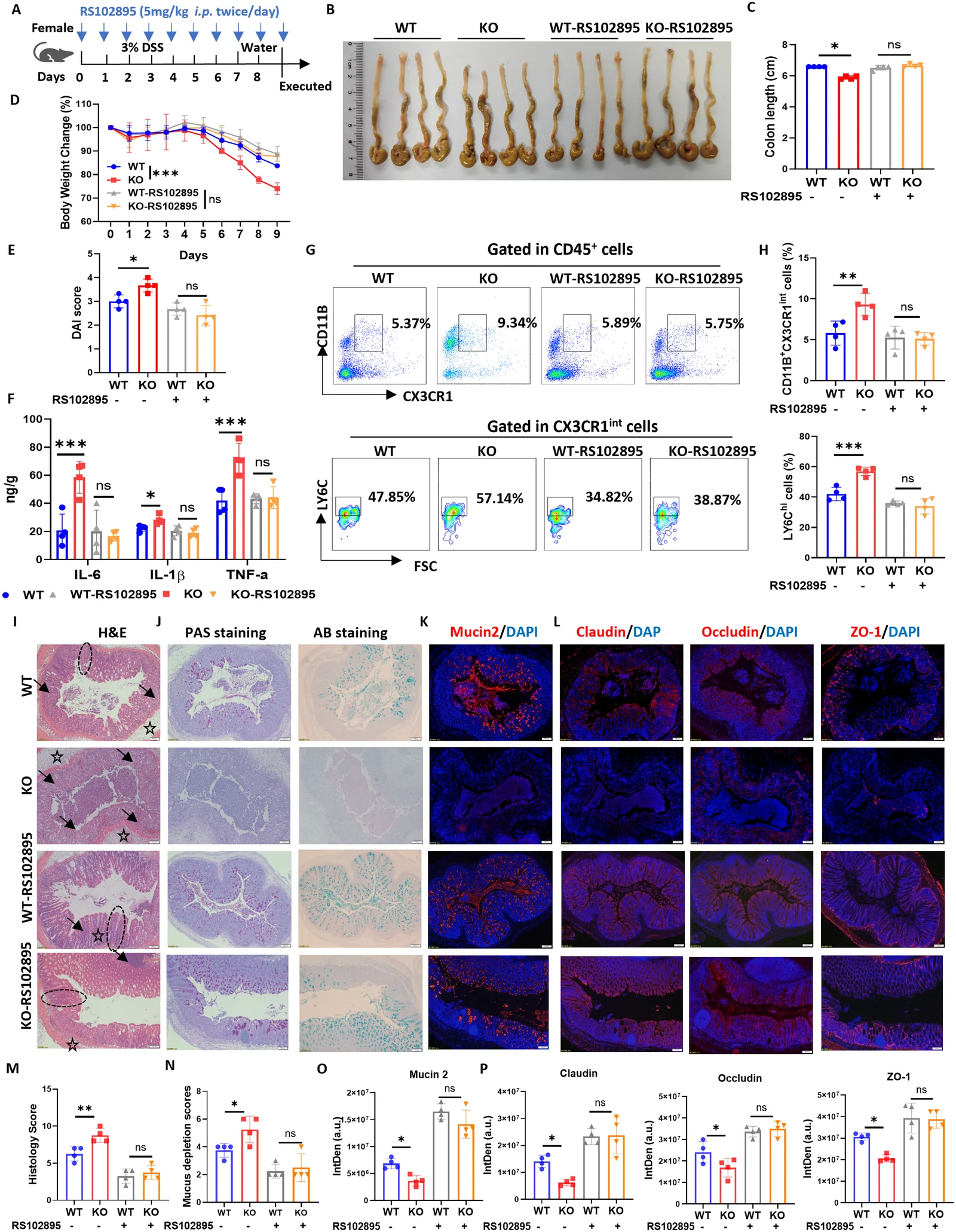
CCR2 antagonist attenuates colitis aggravated by *Timd4* deficiency. **(A)** Therapeutic regimen for DSS-induced colitis: female WT and *Timd4* KO mice received 3% DSS in drinking water for 7 days followed by 2-day recovery. RS102895 (MCE, HY18611A) was formulated in a vehicle containing 1% DMSO, 40% PEG300, 5% Tween-80 and 54% saline. The CCR2 antagonist group received RS102895 (5 mg/kg, intraperitoneal injection, 100 μL, twice daily) during the induction phase. **(B, C)** Gross morphology of mouse colon and colon length measurements recorded on day 9. **(D)** Body weight changes were assessed daily. Statistics on the rate of weight loss on day 9. **(E)** Body weight, stool consistency and rectal bleeding were scored and summated to calculate the DAI scores. **(F)** ELISA measurement of pro-inflammatory cytokines (IL-6, IL-1β, TNF-α) in colon homogenates. **(G)** Representative flow cytometry plots of CD11b^+^CX3CR1^int^ and Ly6C^hi^ cells isolated from the colonic tissues. **(H)** Statistical analysis of CD11b^+^CX3CR1^int^ and Ly6C^hi^ monocyte subpopulations in the colonic tissues. **(I)** Representative H&E staining images of colonic tissues. → Inflammation, ☆ Edema, the black dotted lines highlight the crypt. **(J)** Representative PAS staining images and AB staining images. **(K, L)** Immunofluorescence imaging of intestinal barrier markers including (**K)** Mucin2, (**L)** Claudin, Occludin and ZO-1 (red). Scale bars: 100 μm. **(M)** Statistics on histopathological score. **(N)** Statistics on the total scores of PAS staining and AB staining. **(O, P)** Statistics on immunofluorescence intensity of **(O)** Mucin2, **(P)** Claudin, Occludin and ZO-1. All data are presented as mean ± SEM (*n* = 4). One-way ANOVA was employed to assess differences between multiple groups. Statistical significance was reported as * *p* < 0.05, ** *p* < 0.01, *** *p* < 0.001 and ns, no significance. The data underlying this figure can be found in S1 Data.

Flow cytometry showed higher proportions of CD11b^+^CX3CR1^int^ macrophages and Ly6C^hi^ monocytes in the colonic tissues of *Timd4* KO mice than in those of WT mice in the absence of RS102895. After RS102895 treatment, no statistically significant differences in these cell populations were detected between the two genotypes (Fig 4G and 4H). In addition, compared with WT mice, *Timd4* KO mice exhibited more severe colonic tissue damage in the absence of RS102895. After RS102895 treatment, no statistically significant differences were detected between the WT and *Timd4* KO groups in histopathological scores, mucus depletion scores based on PAS and AB staining, or the fluorescence intensities of Mucin2 and the tight junction proteins Claudin, Occludin and ZO-1 (Fig 4I–4P). Together, these findings suggest that CCR2-dependent monocyte recruitment contributes, at least in part, to the more severe colitis phenotype observed in *Timd4* KO mice.

### TIMD4 stabilizes the LKB1-STRAD-MO25 complex through its intracellular domain

KEGG pathway analysis and GSEA revealed enrichment of the NF-κB signaling pathway in *Timd4* KO macrophages (S7 and S10A Figs). Consistently, *Timd4* deficiency was associated with increased p-P65 levels in a DSS-induced colitis model (S10B and S10C Fig). To determine whether the increased chemokine expression depends on NF‑κB activation, we treated BMDMs with the NF‑κB inhibitor QNZ (S10E and S10J–S10L Fig). QNZ attenuated the increased chemokine expression observed in *Timd4* KO macrophages (S11A Fig), supporting the involvement of NF-κB signaling in TIMD4-associated regulation of chemokine expression. Based on our previous finding that TIMD4 promotes AMPK activation via LKB1 [29] and previous reports showing that AMPK negatively regulates NF‑κB signaling [16,30], we hypothesized that TIMD4 might regulate NF‑κB activation through the LKB1‑AMPK axis.

First, we stimulated PEMs from WT and *Timd4* KO mice with increasing concentrations of LPS (0, 10, 50 and 100 ng/mL). With increasing LPS concentrations, the levels of p‑LKB1 and p‑AMPK progressively decreased in WT macrophages, whereas p‑P65 levels increased. Compared with WT macrophages, *Timd4* KO macrophages exhibited lower p‑LKB1 and p‑AMPK levels and higher levels of p‑P65 across the LPS concentrations examined (S10D and S10G–S10I Fig). To further examine the involvement of AMPK, PEMs were treated with the AMPK inhibitor Compound C. Compound C reduced p-AMPK levels and increased p‑P65 levels, with no statistically significant differences in either p-AMPK or p-P65 levels detected between WT and *Timd4* KO macrophages after Compound C treatment (S10F and S10M–S10O Fig). Consistently, no statistically significant difference in chemokine expression was detected between the two genotypes following Compound C treatment (S11B Fig), supporting the involvement of AMPK in TIMD4-associated regulation of NF‑κB signaling. Similarly, following siRNA-mediated knockdown of LKB1-encoding gene *Stk11*, no statistically significant differences in p‑LKB1, p‑AMPK and p‑P65 or chemokine expression were detected between WT and *Timd4* KO macrophages (S10P–S10S and S11C Figs). These results support the involvement of LKB1 in TIMD4-associated regulation of the AMPK-NF-κB signaling.

To elucidate the molecular basis underlying this regulatory axis, we next examined the effects of TIMD4 on LKB1 phosphorylation and subcellular localization. TIMD4 overexpression in HEK293T cells increased LKB1 phosphorylation, whereas *Timd4* deficiency in PEMs was associated with reduced LKB1 phosphorylation (Fig 5A–5D). Formation of the LKB1-STRAD-MO25 complex promotes the translocation of LKB1 from the nucleus to the cytoplasm and its association with the plasma membrane, thereby facilitating AMPK activation [19,31]. Subcellular fractionation showed that TIMD4 overexpression increased LKB1 membrane localization and reduced its nuclear localization in HEK293T cells (Fig 5E and 5F). Immunofluorescence analysis further showed a higher membrane/cytoplasmic-to-nuclear fluorescence ratio of LKB1 in TIMD4-transfected HEK293T cells and WT PEMs than in their respective controls (Fig 5H–5K).

**Fig 5:**
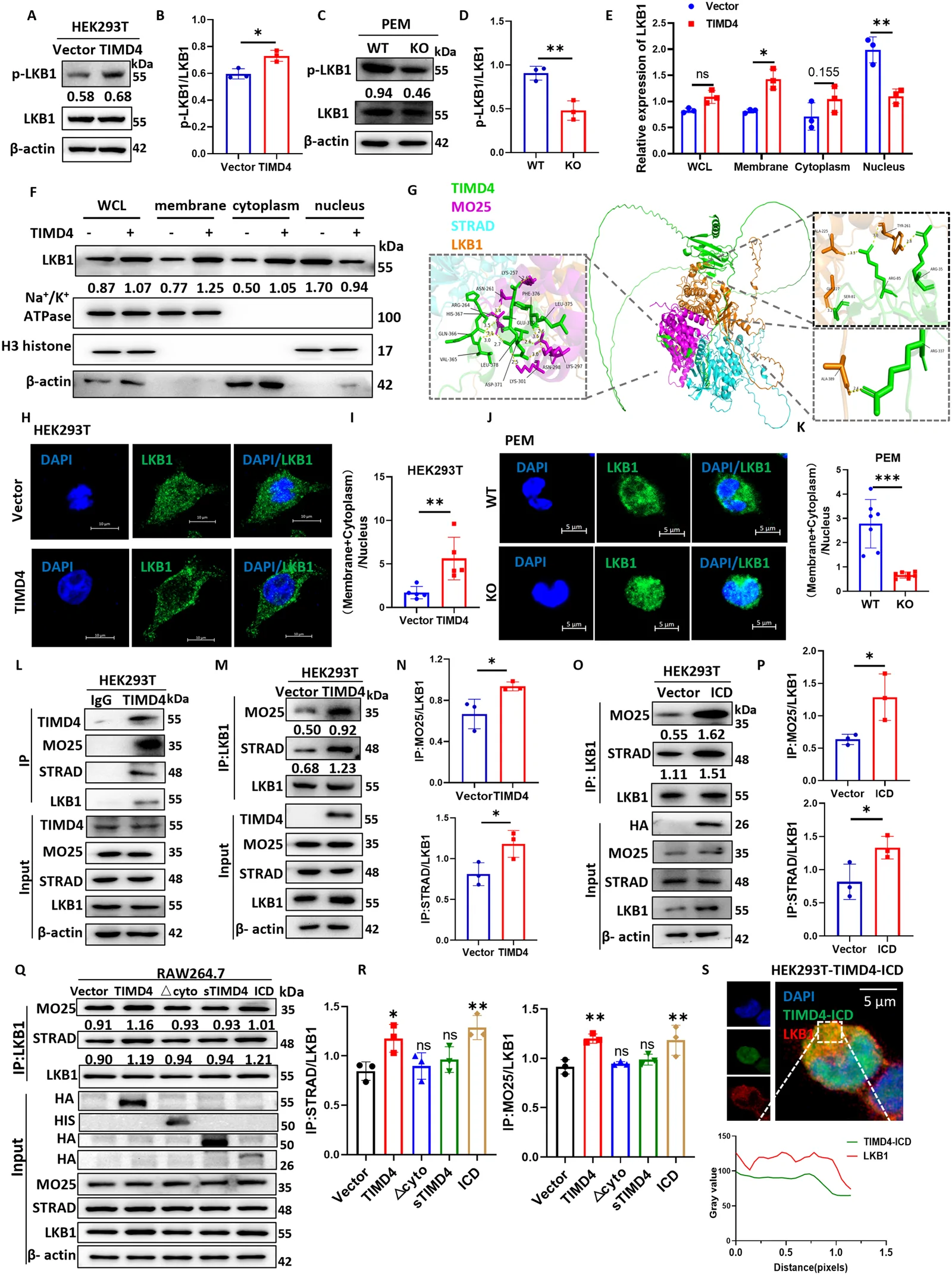
TIMD4 promotes LKB1 phosphorylation by stabilizing the LKB1-STRAD-MO25 complex. **(A, B)** Western blotting analysis of total LKB1 and phospho-LKB1 (Thr189/Ser428) in *TIMD4*-transfected HEK293T cells. Quantification of the phospho-LKB1/total LKB1 ratio from three independent experiments. **(C, D)** Western blotting analysis of total LKB1 and phospho-LKB1 in WT and *Timd4* KO PEMs. Quantification of the phospho-LKB1/total LKB1 ratio from three independent experiments. **(E)** Quantification of LKB1 protein levels in whole-cell lysate (WCL) and each subcellular fraction from three independent experiments. (Na, K-ATPase for membrane, β-actin for cytoplasm and H3 histone for nucleus). **(F)** Subcellular fractionation analysis of LKB1 protein levels in whole-cell lysate (WCL), membrane, cytoplasm and nuclear fractions of HEK293T cells. Western blotting images of LKB1 are shown. **(G)** AlphaFold 3-based structural modeling of the potential interactions among TIMD4, MO25, STRAD and LKB1. **(H, I)** Immunofluorescence imaging of LKB1 (green) localization in HEK293T cells transfected with empty vector *vs*. TIMD4 (Scale bars: 10 μm). Quantification of the ratio of the sum of membrane and cytoplasmic fluorescence intensity to nuclear fluorescence intensity in HEK293T cells. **(J, K)** Immunofluorescence imaging of LKB1 (green) localization in WT and *Timd4* KO PEMs (Scale bars: 5 μm). Quantification of the ratio of the sum of membrane and cytoplasmic fluorescence intensity to nuclear fluorescence intensity in PEMs. **(L)** Co-IP analysis in HEK293T cells transiently expressing *TIMD4* using anti-TIMD4 antibody or IgG control. **(M, N)** Co-IP assays with anti-LKB1 antibody in HEK293T cells transfected with empty vector (pcDNA3.0) and full-length *TIMD4*. Quantification of MO25 and STRAD protein levels in the IP relative to LKB1. **(O, P)** Co-IP assays with anti-LKB1 antibody in HEK293T cells transfected with pcDNA3.0 *vs*. TIMD4 intracellular domain (ICD). Quantification of MO25 and STRAD protein levels in the IP relative to LKB1. **(Q, R)** IP assays were performed using anti‑LKB1 antibody in RAW264.7 cells transfected with empty vector, TIMD4, Δcyto, sTIMD4, or ICD. Representative Western blotting images and quantification of MO25 and STRAD protein levels in the IP relative to LKB1 are shown. **(S)** Confocal microscopy demonstrating co-localization of TIMD4 ICD (GFP) and endogenous LKB1 (red) in HEK293T cells (scale bars: 5 μm). All data are presented as mean ± SEM (*n* = 3). The unpaired Student’s *t* test, one-way ANOVA **(R)** and two-way ANOVA **(E)** were employed to assess differences between two and multiple groups. Statistical significance was reported as * *p* < 0.05, ** *p* < 0.01, *** *p* < 0.001 and ns, no significance. The data underlying this figure can be found in S1 Data.

The results of AlphaFold 3-based structural modeling suggested potential interaction interfaces between TIMD4 and the MO25 and LKB1 subunits of the LKB1-STRAD-MO25 complex, with the majority of the predicted hydrogen bonds formed by residues within the intracellular domain (ICD) of TIMD4 (Fig 5G). Co-immunoprecipitation (Co-IP) assays showed associations between TIMD4 and the LKB1-STRAD-MO25 complex in HEK293T cells (Fig 5L). Overexpression of full-length TIMD4 or TIMD4 ICD increased the amounts of MO25 and STRAD immunoprecipitated with LKB1 (Fig 5M–5P), whereas TIMD4 truncation mutants lacking the intracellular domain (TIMD4 Δcyto) and soluble TIMD4 (sTIMD4) did not increase the amounts of MO25 and STRAD immunoprecipitated with LKB1 (S12A–S12D Fig). Similar results were obtained following the expression of full-length TIMD4 and TIMD4 ICD in RAW264.7 cells (Fig 5Q and 5R). Confocal immunofluorescence microscopy also showed subcellular co-localization of the TIMD4 ICD with LKB1 in HEK293T cells (Fig 5S). In addition, TIMD4 expression was associated with reduced ubiquitination and proteasomal degradation of LKB1 (S13 Fig).

Together, these in vitro findings support a role for the intracellular domain of TIMD4 in stabilizing the LKB1-STRAD-MO25 complex and modulating downstream AMPK–NF-κB signaling.

### Macrophage-targeted delivery of a TIMD4-derived peptide attenuates DSS-induced colitis in mice

Based on the interaction interfaces predicted by AlphaFold 3 (Fig 5G), we engineered a plasmid P14 encoding a TIMD4-derived peptide encompassing residues 365–378 (Fig 6A). P14 increased the amounts of MO25 and STRAD immunoprecipitated with LKB1 (S12E and S12F Fig). To enable macrophage-targeted delivery, mannose-modified hydroxyapatite (HAp) nanoparticles (NPs) were developed for plasmid and peptide loading (Fig 6B). Transmission electron microscopy analysis showed uniform dispersion of the NPs after ultrasonication (Fig 6C). Fourier-transform infrared (FTIR) spectroscopy revealed a prominent absorption peak in the 1,618–1,710 cm^−1^ region, providing evidence for plasmid loading onto the nanoparticles (Fig 6D). Agarose gel electrophoresis further showed no detectable plasmid degradation following loading (Fig 6E). In vitro experiments showed efficient delivery of P14-NPs to BMDMs (Fig 6F). Subsequent analyses detected P14 expression and showed reduced P65 phosphorylation in P14-NPs-treated BMDMs (Fig 6G and 6H).

**Fig 6:**
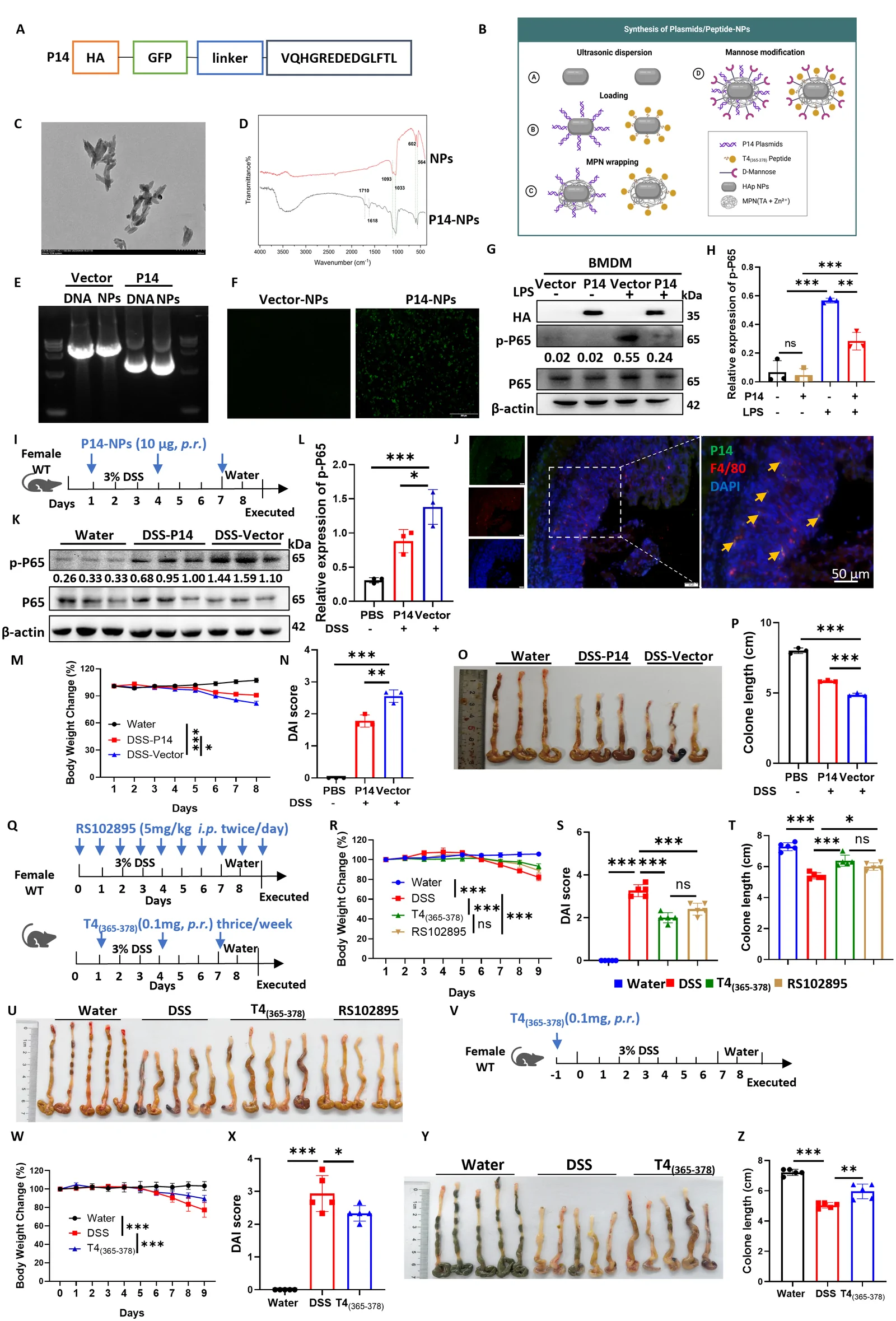
D-mannose-modified HAp-NPs deliver TIMD4-derived peptide to macrophages, reduce P65 phosphorylation and attenuate DSS-induced colitis. **(A)** A TIMD4-derived peptide encompassing residues 365–378 was selected based on the interaction interfaces predicted by AlphaFold 3 and encoded by the P14 plasmid. **(B)** Synthesis of P14/T4_(365–378)_-NPs. Ultrasonic dispersion: HAp NPs were dispersed in nuclease-free ddH_2_O by brief ultrasonication; Loading: The plasmid or peptide was mixed with HAp NPs to generate plasmid- or peptide-loading HAp NPs; Metal-phenol network wrapping: Tannic acid and ZnCl_2_ were added to the plasmid- or peptide-HAp suspension; Mannose modification: D-mannose was added, and the mixture was incubated for 8 **h.** The schematic was created in BioRender. Huimin, **L.** (2026) https://BioRender.com/l509tv6. **(C)** The morphology of the nanoparticles was analyzed using a transmission electron microscope. **(D)** FTIR spectra were recorded using a FTIR spectrometer (Bruker Alpha-II, Germany) with a spectral resolution of 4 cm^−1^. **(E)** Nucleic acid gels of Vector and P14 plasmids and Vector-NPs and P14-NPs. **(F)** Transfection efficiency of macrophages was observed under fluorescence microscopy. **(G, H)** Western blotting analysis of P65 and p-P65 levels in Vector and P14-transfected macrophages. **(I)** P14-NPs were administered by enema to *Timd4* KO mice (10 μg/mouse, three times/week). **(J)** Immunofluorescence staining of colonic tissue (Green: P14-GFP, Red: F4/80, Blue: DAPI, Scale bars: 50 μm). Arrows indicate P14-positive macrophages. **(K)** Representative Western blotting images of P65 and p-P65 in mouse colonic tissues. **(L)** Statistical analysis on relative expression levels of p-P65. **(M)** Body weight changes were assessed daily. Statistics on the rate of weight loss on day 9. **(N)** Body weight, stool consistency and rectal bleeding were scored and summated to calculate the DAI scores. **(O, P)** Gross morphology of mouse colon and colon length measurements recorded on day 9. **(Q)** Schematic diagram of the acute colitis model and treatment regimen in WT C57BL/6J mice. The CCR2 antagonist group received RS102895 (5 mg/kg, intraperitoneal injection, *i.p.*, twice daily). The T4_(365–378)_-NPs treatment group received the TIMD4 peptide via colonic administration (0.1 mg) on days 1, 4 and 7. **(R)** Body weight changes were assessed daily. Statistics on the rate of weight loss on day 9. **(S)** Body weight, stool consistency and rectal bleeding were scored and summated to calculate the DAI scores. **(T)** The quantitative analysis of colon length. **(U)** Representative images of colon. **(V)** Experimental protocol: WT C57BL/6J mice received a single intrarectal instillation of T4_(365‑378)_-NPs (0.1 mg) one day before the start of 3% DSS in drinking water; DSS was administered for 7 consecutive days, followed by water for 2 days. **(W)** Body weight changes were assessed daily. Statistics on the rate of weight loss on day 9. **(X)** Body weight, stool consistency and rectal bleeding were scored and summated to calculate the DAI scores. **(Y)** Representative images of colon. **(Z)** The quantitative analysis of colon length. All data are presented as mean ± SEM (*n* ≥ 3). One-way ANOVA was employed to assess differences between multiple groups. Statistical significance was reported as * *p* < 0.05, ** *p* < 0.01, *** *p* < 0.001 and ns, no significance. The data underlying this figure can be found in S1 Data.

To evaluate the effects of P14-NPs in vivo, P14-NPs were administered by enema three times per week to *Timd4* KO mice during DSS-induced acute colitis (Fig 6I). Immunofluorescence analysis of colonic tissues showed co-localization of P14-NPs with macrophages (Fig 6J), together with reduced P65 phosphorylation (Fig 6K and 6L). Treatment with P14-NPs reduced body weight loss (Fig 6M), DAI score (Fig 6N) and attenuated colon shortening in *Timd4* KO mice (Fig 6O and 6P). Treatment with P14-NPs also reduced colonic levels of pro-inflammatory cytokines and chemokines (S14A and S14B Fig). Histopathological analysis further showed reduced inflammatory cell infiltration and lower histopathological scores following treatment with P14-NPs (S14C and S14G Fig). Treatment with P14-NPs also reduced mucus depletion scores based on PAS and AB staining and increased fluorescence intensities of Mucin2, Claudin, Occludin and ZO-1 (S14D–S14F and S14H–S14J Fig). Treatment with P14-NPs also alleviated colitis severity in WT mice (S15 Fig). Together, these findings show that macrophage-targeted delivery of P14 was associated with reduced NF-κB signaling and attenuation of DSS-induced colitis in both *Timd4* KO and WT mice.

Based on these findings, we synthesized the corresponding TIMD4-derived peptide, T4_(365–378)_, and developed a T4_(365–378)_-NPs delivery system. To compare the efficacy of T4_(365–378)_-NPs and the CCR2 antagonist RS102895, a parallel treatment comparison was performed in WT mice (Fig 6Q). Both the T4_(365–378)_-NPs and RS102895 treatment reduced body weight loss and the DAI score and attenuated colon shortening (Fig 6R–6U). Both treatments also reduced histopathological damage and mucus depletion scores based on PAS and AB staining and increased Mucin2 fluorescence intensity (S16A–S16C and S16E–S16G Fig). Furthermore, both treatments increased the fluorescence intensities of Claudin, Occludin and ZO-1, with no statistically significant differences detected between the treatment groups (S16D and S16H Fig).

Given the potential instability of small peptides, we further established a pretreatment model in WT mice to determine whether T4_(365–378)_-NPs administered before DSS exposure could attenuate subsequent colitis (Fig 6V). In this model, T4_(365–378)_-NPs pretreatment reduced body weight loss (Fig 6W) and DAI score (Fig 6X), attenuated colon shortening (Fig 6Y and 6Z), and lowered histopathological scores (S17A and S17E Fig). T4_(365–378)_-NPs pretreatment was also associated with lower mucus depletion scores based on PAS and AB staining and increased fluorescence intensities of Mucin2, Claudin, Occludin and ZO-1 (S17B–S17D and S17F–S17H Fig).

Together, these findings indicate that macrophage-targeted delivery of TIMD4-derived peptides attenuated DSS-induced colitis in both *Timd4* KO and WT mice. In WT mice, T4_(365–378)_-NPs and RS102895 each attenuated disease severity and improved intestinal barrier-associated markers, while the effects of T4_(365–378)_-NPs were also observed when administered before DSS exposure. These results support further preclinical investigation of macrophage-targeted TIMD4-derived peptide delivery as a potential approach for modulating intestinal inflammation.

### Combination of T4_(365–378)_-NPs with anti-α4β7 or anti-TNF-α antibodies attenuates DSS-induced colitis in mice

Biologic therapies targeting α4β7 integrin or TNF-α are used in the clinical treatment of IBD. We next evaluated T4_(365–378)_-NPs in combination with antibodies targeting these molecules in WT mice with DSS-induced colitis. We first examined combination treatment with an anti-α4β7 antibody (Fig 7A). In the DSS-induced acute colitis model, the combination therapy group showed greater improvements than either monotherapy, including mitigated body weight loss (Fig 7B), lower DAI score (Fig 7C) and greater colon length (Fig 7D and 7E). The combination of T4_(365–378)_-NPs and anti-α4β7 led to a greater reduction in P65 phosphorylation levels compared with either treatment alone (Fig 7F and 7G). Compared with the anti-α4β7 monotherapy, combination treatment reduced the expression of monocyte-recruiting chemokines (Fig 7H) and pro-inflammatory cytokines (Fig 7I) in colonic tissues. H&E staining showed reduced inflammatory cell infiltration, less crypt disruption and diminished intestinal hemorrhage, together with lower histopathological scores, in the combination group (Fig 7J and 7N). Furthermore, the combination therapy reduced mucus depletion scores based on PAS and AB staining (Fig 7K and 7O) and increased Mucin2 fluorescence intensity (Fig 7L and 7P) and the fluorescence intensities of tight junction proteins, including Claudin, Occludin and ZO-1 (Fig 7M and 7Q).

**Fig 7:**
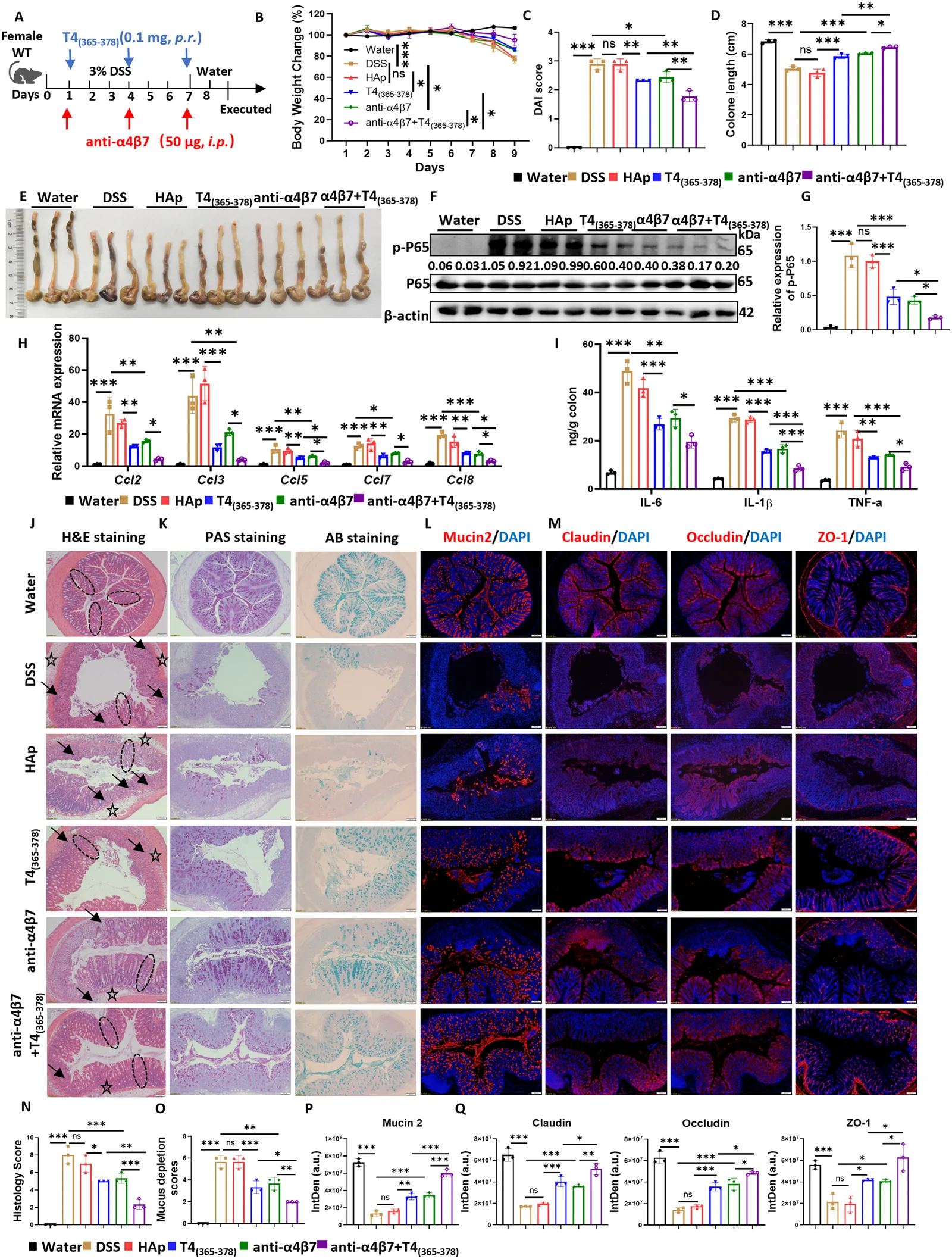
Combination treatment with T4_(365–378)_-NPs and anti-α4β7 attenuates DSS-induced acute colitis. **(A)** Female WT C57BL/6J mice aged 6–8‑weeks were randomly assigned to six groups. Therapeutic regimen for DSS-induced colitis: T4_(365–378)_-NPs (0.1 mg/mouse, *p.r.*, 3 times/week) combined with anti-α4β7 antibody (BioLegend, clone DATK32, 50 μg/mouse, *i.p.*, 3 times/week). Amino acid sequence of T4_(365–378)_: VQHGREDEDGLFTL. **(B)** Body weight changes were assessed daily. Statistics on the rate of weight loss on day 9. **(C)** Body weight, stool consistency and rectal bleeding were scored and summated to calculate the DAI scores. **(D, E)** Representative images and quantitative analysis of colon length on day 9. **(F)** Representative Western blotting images of P65 and p-P65 in mouse colonic tissues. **(G)** Statistical analysis on relative expression levels of p-P65. **(H)** RT-qPCR quantification of chemokine expression (*Ccl2, Ccl3, Ccl5, Ccl7, Ccl8*) in mouse colonic tissues. **(I)** ELISA measurement of pro-inflammatory cytokines including IL-6, IL-1β, TNF-α in colonic tissue homogenates. **(J)** Representative H&E-stained images of colonic sections. → Inflammation, ☆ Edema, the black dotted lines highlight the crypt. **(K)** Representative images of PAS and AB staining in colonic sections. **(L, M)** Immunofluorescence imaging of **(L)** Mucin2 and **(M)** tight junction proteins including Claudin, Occludin and ZO-1 (red) in colon sections (scale bars: 100 μm). **(N)** Statistical analysis of histopathological scores. **(O)** Statistical analysis of mucus depletion scores based on PAS and AB staining. **(P, Q)** Statistics on immunofluorescence intensity of **(P)** Mucin2, **(Q)** Claudin, Occludin and ZO-1. All data are presented as mean ± SEM (*n* = 3). One-way ANOVA was employed to assess differences between multiple groups. Statistical significance was reported as * *p* < 0.05, ** *p* < 0.01, *** *p* < 0.001 and ns, no significance. The data underlying this figure can be found in S1 Data.

To evaluate the effects of combination treatment during the active phase of DSS-induced colitis, treatment with anti-α4β7 and T4_(365–378)_-NPs was initiated on day 4 of DSS administration (S18A Fig). Compared with either monotherapy, the combination therapy significantly attenuated body weight loss (S18B Fig), reduced the DAI score (S18E Fig) and attenuated colon shortening (S18C and S18D Fig). Combination treatment also resulted in lower histopathological scores (S18F and S18J Fig), lower mucus depletion scores based on PAS and AB staining (S18G and S18K Fig), and increased fluorescence intensities of Mucin2 and the tight junction proteins Claudin, Occludin and ZO-1 (S18H, S18I, S18L and S18M Fig).

Furthermore, we conducted additional experiments in WT mice with DSS-induced colitis to evaluate T4_(365–378)_-NPs in combination with anti‑TNF‑α (Fig 8A). Compared with mice receiving either monotherapy, the combined regimen exhibited less body weight loss (Fig 8B), lower DAI scores (Fig 8E) and greater recovery of colonic length (Fig 8C and 8D). Histopathological evaluation revealed markedly less mucosal disruption, crypt destruction and leukocyte infiltration in the dual‑treatment group (Fig 8F and 8J). Moreover, mucus depletion scores based on PAS and AB staining were lower and Mucin2 fluorescence intensity was higher in the combination group than in either monotherapy group (Fig 8G, 8H, 8K and 8L). Additionally, the fluorescence intensities of the tight junction proteins Claudin, Occludin and ZO‑1 were higher in the combination group (Fig 8I and 8M).

**Fig 8:**
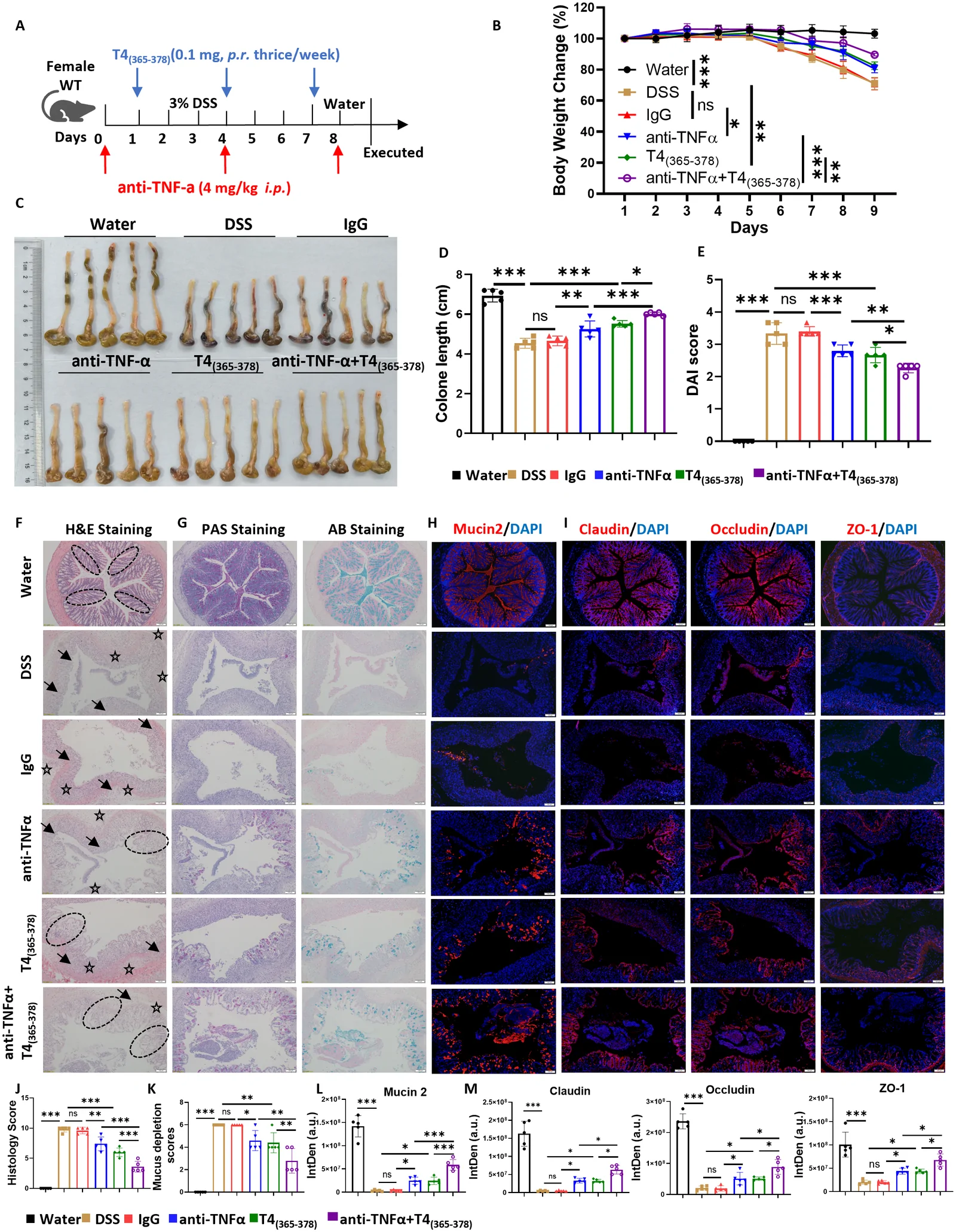
Combination treatment with T4_(365–378)_-NPs and anti-TNF-α attenuates DSS-induced acute colitis. **(A)** Experimental scheme of combination therapy. 6–8‑week‑old female C57BL/6J mice were randomly assigned to six groups: Water group; DSS group; anti-TNF-α group (anti‑mouse TNF-α antibody, XT3.11, Selleck, 4 mg/kg, *i.p.*, on day 1, 4, 8); Isotype control IgG group (Ultra‑LEAF Purified Rat IgG2a, BioLegend, 400544, 4 mg/kg, *i.p.* on day 1, 4, 8); T4_(365‑378)_‑NPs group (0.1 mg, *p.r.* on day 2, 4, 7); Combination therapy group (received both anti-TNF-α and T4_(365‑378)_‑NPs according to the above regimens). **(B)** Body weight changes were assessed daily. Statistics on the rate of weight loss on day 9. **(C)** Representative images and **(D)** quantitative analysis of colonic length. **(E)** Body weight, stool consistency and rectal bleeding were scored and summated to calculate the DAI scores. **(F)** Representative H&E staining images of colonic tissues. → Inflammation, ☆ Edema, the black dotted lines highlight the crypt. **(G)** Representative PAS staining images and AB staining images. **(H, I)** Representative immunofluorescence staining images of Mucin2, Claudin, Occludin and ZO-1. Scale bars: 100 μm. **(J)** Statistical analysis of histopathological scores. **(K)** Statistical analysis of mucus depletion scores based on PAS and AB staining. **(L, M)** Quantification of mean fluorescence intensity of Mucin2, Claudin, Occludin and ZO-1. Data are presented as mean ± SEM (*n* = 5). One-way ANOVA was employed to assess differences between multiple groups. Statistical significance was reported as * *p* < 0.05, ** *p* < 0.01, *** *p* < 0.001 and ns, no significance. The data underlying this figure can be found in S1 Data.

In summary, the combination of T4_(365‑378)_-NPs with either anti‑α4β7 or anti‑TNF‑α produced greater improvements in the assessed disease and intestinal barrier-associated outcomes than the corresponding antibody monotherapy in the DSS‑induced colitis model. These findings support further preclinical evaluation of T4_(365–378)_-NPs in combination with established therapeutic antibodies for intestinal inflammation.

## Discussion

Although TIMD4 is known to mediate apoptotic cell clearance and promote the establishment of immune tolerance, its regulatory function and underlying network in intestinal inflammation have not yet been defined. Collectively, these data suggest that TIMD4 may protect against DSS-induced colitis, at least in part, by regulating chemokine production via the LKB1-AMPK-NF-κB axis, thereby potentially limiting monocyte infiltration and local immune responses. Mechanistically, in vitro experiments suggest that the intracellular domain of TIMD4 enhances the stability of the LKB1-STRAD-MO25 complex, thereby promoting LKB1 phosphorylation. Macrophage-targeted delivery of a TIMD4-derived peptide reduced P65 phosphorylation and attenuated DSS-induced colitis in mice. Furthermore, combining T4_(365–378)_-NPs with either anti-α4β7 or anti-TNF-α antibodies produced greater improvements than the corresponding antibody monotherapy in the DSS-induced colitis model. These findings support further preclinical evaluation of macrophage-targeted TIMD4-derived peptide delivery for intestinal inflammation. A schematic summary of the findings is presented in Fig 9.

**Fig 9:**
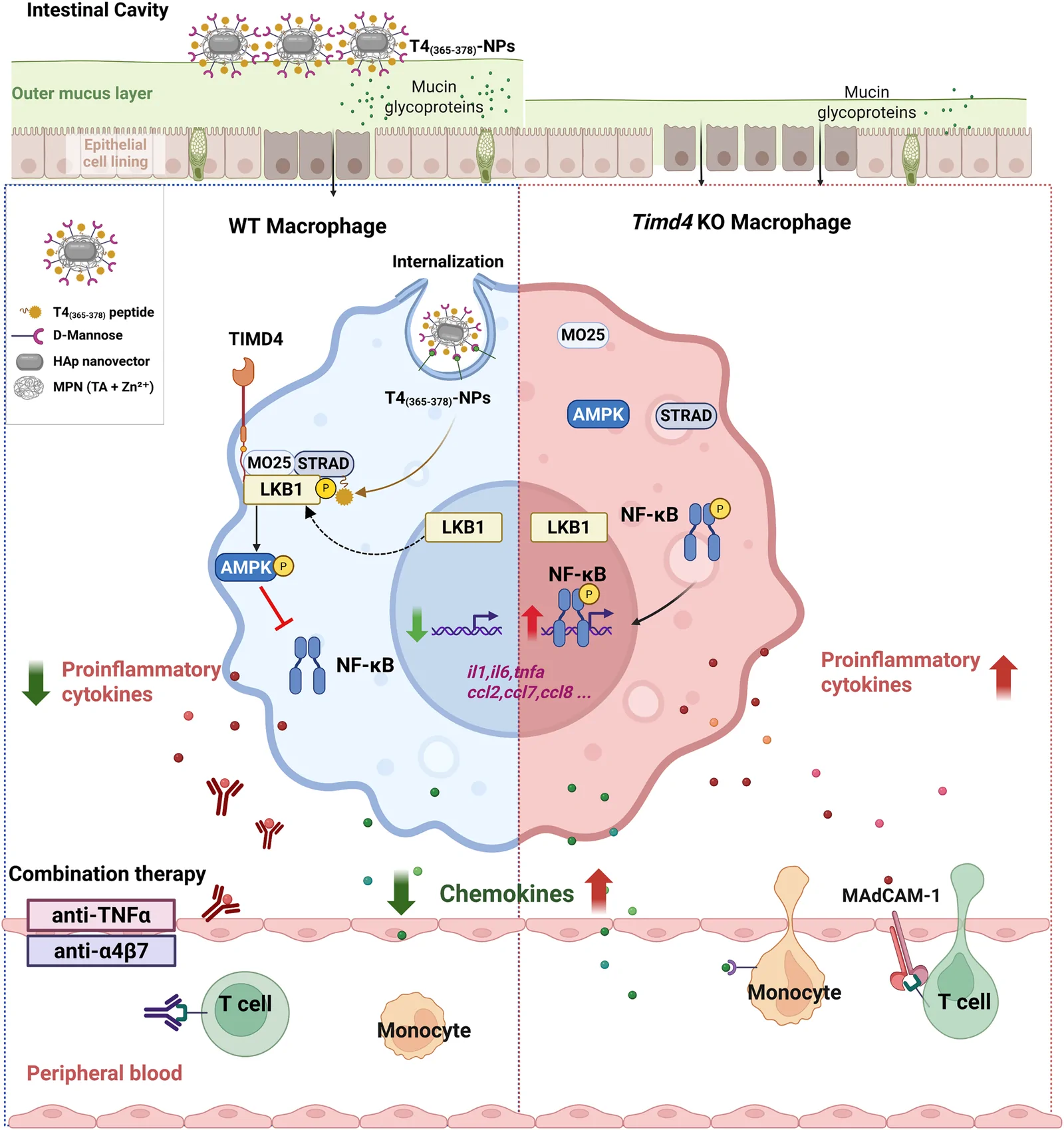
Graphical Abstract: The combination therapy of T4_(365–378)_-NPs with anti-α4β7 or anti-TNF-α for the treatment of DSS-induced colitis. The intracellular domain of TIMD4 regulates the LKB1/AMPK/NF-κB pathway by stabilizing the LKB1-STRAD-MO25 complex, thereby participating in the regulation of chemokine production. The derived peptide fragment TIMD4_(365–378)_, delivered to intestinal macrophages via HAp-NPs, is associated with reduced NF-κB activation. Its combination with anti-α4β7 or anti-TNF-α represents a dual-targeted therapeutic strategy addressing distinct inflammatory processes. Created in BioRender. Huimin, **L.** (2026) https://BioRender.com/p0e3rr2.

As an immunoregulatory molecule, TIMD4 contributes to tissue immune homeostasis by modulating pro- and anti-inflammatory responses in macrophages [23,25,32]. In this study, TIMD4 protein levels were increased in colonic tissues from patients with UC and mice with DSS-induced colitis, suggesting that TIMD4 upregulation may represent an adaptive response to intestinal inflammation. Consistent with a potential protective role for TIMD4, *Timd4* KO mice exhibited more severe DSS-induced colitis than WT mice. Given the predominant expression of TIMD4 in intestinal macrophages, we next examined the contribution of macrophages to this protective phenotype. Macrophage depletion aggravated DSS-induced colitis in both WT and *Timd4* KO mice, consistent with a protective role for macrophages in this model. Following macrophage depletion, no statistically significant differences in colon length, body weight loss or histopathological scores were detected between the two genotypes. These findings suggest that the protective effects of macrophages during DSS-induced colitis may be mediated, at least in part, by TIMD4. Analysis of patient specimens revealed that patients with higher disease activity scores exhibited lower TIMD4 expression in colonic macrophages, supporting a potential role for macrophage TIMD4 in regulating the severity of intestinal inflammation. In addition, we observed a significant increase in apoptotic cell accumulation in the colonic tissues of *Timd4* KO mice. Given the established role of TIMD4 as an efferocytic receptor, this finding suggests that *Timd4* deficiency may also impair apoptotic cell clearance. Together, these findings suggest that macrophage TIMD4 contributes to intestinal immune homeostasis and that impaired TIMD4 function may promote persistent inflammation and tissue injury.

The dynamic equilibrium of macrophage subpopulations is a critical mechanism for maintaining intestinal immune homeostasis. Under physiological conditions, CD11b⁺CX3CR1^hi^Ly6C^−^ resident macrophages predominate and contribute to the establishment of an anti-inflammatory milieu [33,34]. In contrast, inflammatory challenge can activate the CCL2-CCR2 axis, which drives the infiltration of CD11b⁺CX3CR1^int^Ly6C^hi^ monocyte-derived inflammatory macrophages, thereby fostering a pro-inflammatory microenvironment [35,36]. In this study, *Timd4* deficiency was associated with enrichment of chemokine signaling pathways, increased expression of monocyte-recruiting chemokines and greater accumulation of Ly6C^hi^ inflammatory monocytes during DSS-induced colitis. Pharmacological blockade of CCR2 attenuated colitis and reduced colonic monocyte accumulation in *Timd4* KO mice, suggesting that CCR2-dependent monocyte recruitment may contribute to the aggravated colitis associated with *Timd4* deficiency. Beyond its association with monocyte recruitment, *Timd4* deficiency was also accompanied by increased expression of CXCL family chemokines implicated in the recruitment of Th1, Th17 and neutrophils [37,38]. To examine the relevance of these findings to human UC, analysis of the GSE16879 dataset revealed that UC samples with low *TIMD4* expression showed enrichment of leukocyte migration and chemotaxis-related pathways relative to samples with high expression. Together, these findings suggest that macrophage TIMD4 contributes to maintaining monocyte–macrophage subset balance and limiting the chemokine-mediated recruitment of other inflammatory immune cells during DSS-induced colitis.

Hyperactivation of the NF-κB pathway, a major contributor to colonic inflammation, promotes the production of pro-inflammatory cytokines and induces CCL2-mediated monocyte recruitment [14,15,39,40], a pattern that aligns with our findings in murine colitis models. In contrast to these pro-inflammatory effects, AMPK signaling can negatively regulate NF-κB-dependent inflammation through downstream effectors including SIRT1, PGC-1α, p53 and forkhead box O factors [16,17,29,30]. Given our previous finding that TIMD4 promotes AMPK activation through the upstream kinase LKB1 [29], we hypothesized that TIMD4 may regulate NF-κB-dependent inflammatory responses through the LKB1–AMPK axis. Consistent with this possibility, the pharmacological inhibition of AMPK and siRNA-mediated knockdown of *Stk11* in the present study support the involvement of the LKB1–AMPK–NF-κB axis in TIMD4-associated regulation of macrophage chemokine expression. Our in vitro findings further suggest that the C-terminal intracellular domain of TIMD4 enhances the stability of the LKB1-STRAD-MO25 complex and is associated with increased LKB1 membrane localization and phosphorylation. This is functionally significant, given that the kinase activity of LKB1 is strictly dependent on complex assembly with STRAD and MO25 [41,42]. Formation of this heterotrimeric complex promotes the translocation of LKB1 from the nucleus to the cytoplasm and its association with cellular membranes, thereby facilitating AMPK phosphorylation [19,31,43]. TIMD4 expression was also associated with reduced ubiquitination and proteasomal degradation of LKB1. However, whether TIMD4 directly regulates LKB1 ubiquitination remains to be determined.

Building upon the protective role of TIMD4 in macrophages, we developed mannose-modified, macrophage-targeting HAp nanoparticles designed to deliver a peptide derived from TIMD4 (residues 365–378). Previous studies have shown that mannose modification can promote nanoparticle uptake by macrophages through mannose receptor-mediated endocytosis [44]. Following internalization, the acidic endosomal environment may promote the dissolution of hydroxyapatite and other calcium phosphate-based carriers, thereby facilitating the intracellular release of their cargo [45]. These processes provide a plausible route through which T4_(365–378)_ could gain access to intracellular targets within colonic macrophages. However, the intracellular trafficking and cytosolic release of T4_(365–378)_-NPs were not directly examined in the present study and require further investigation. Administration of T4_(365–378)_-NPs was associated with reduced P65 phosphorylation and lower colonic levels of pro-inflammatory cytokines and chemokines, together with attenuation of DSS-induced colitis. To evaluate the potential of this strategy to complement existing biologic therapies for IBD, we combined T4_(365–378)_-NPs with anti-α4β7 or anti-TNF-α antibodies. These combination regimens were designed to target distinct inflammatory processes involved in DSS-induced colitis, achieving coordinated modulation of both innate and adaptive immunity. In the DSS-induced colitis model, these two combination regimens produced greater improvements in disease severity and intestinal barrier-associated outcomes relative to each monotherapy. Together, these findings provide a preclinical basis for exploring macrophage-targeted delivery of T4_(365–378)_ in combination with established biologic therapies as a potential therapeutic strategy for IBD.

In conclusion, this study supports a protective role for macrophage TIMD4 in DSS-induced colitis, associated with the regulation of chemokine production through the LKB1-AMPK-NF-κB axis and the maintenance of monocyte–macrophage subset balance. Building on these mechanistic findings, we developed a macrophage-targeted formulation of T4_(365–378)_-NPs that significantly inhibited NF-κB activation and attenuated DSS-induced colitis. Furthermore, combination therapy with T4_(365–378)_-NPs and either anti-α4β7 or anti-TNF-α produced greater amelioration of colonic inflammation in mice than the corresponding antibody monotherapy. Collectively, these findings provide mechanistic insights into the immunoregulatory function of macrophage TIMD4 and a preclinical basis for exploring macrophage-targeted TIMD4-derived peptide delivery, alone or in combination with established biologic therapies, for intestinal inflammation.

## Materials and methods

### Ethics statement

All mice were maintained in specific pathogen-free and temperature-controlled conditions. All animal procedures were reviewed and approved by the Animal Care and Use Committee of Shandong University (Approval No: SBM5SDU2019-2-028). The collection and use of human specimens were approved by the Research Ethics Committee of Qilu Hospital of Shandong University (Approval No: KYLL-202312-039-1). Written informed consent was obtained from all participants before specimen collection.

### Mice and DSS-induced colitis models

*Timd4* KO mice were provided by the RIKEN BioResource Research Center (RBRC04895) [46,47]. In the colitis experiments using *Timd4* KO and WT mice, *Timd4* KO and WT littermates were used to maintain a consistent genetic background between groups. For the subsequent TIMD4-derived peptide nanoparticle treatment experiments, C57BL/6J WT mice were obtained from Vital River Laboratory Animal Technology. Mice (6–8 weeks old) were randomly divided into experimental and control groups. To establish acute colitis, mice received 2%, 3% or 4% (w/v) DSS (molecular weight 36,000–50,000, MP Biomedicals) in regular drinking water for 7 consecutive days, depending on the experimental protocol, followed by regular drinking water for 2 days. The DAI score was calculated by combining body weight loss, stool consistency and fecal occult blood using a standardized scoring system (S1 Table). Finally, mice were euthanized by CO_2_ asphyxiation, and colonic tissues were excised for photography and measurement of colon length.

For macrophage depletion experiments, 150 μL of Clodronate-Liposomes (CLs) (LIPOSOMA, C-005) was administered intraperitoneally every other day during the induction period of DSS colitis. The dose was selected based on the manufacturer’s recommendations and a previous study [48]. Macrophage depletion efficiency was evaluated by flow cytometry analysis.

### Isolation of colonic mononuclear cells

Colons were removed, cleared of mesenteric tissue and fecal contents, cut into 0.5- to 1-cm segments and thoroughly washed with ice-cold PBS. Initial digestion of the colonic tissues was performed using a dissociation solution containing RPMI 1640 medium, 2% fetal bovine serum (FBS), N-(2-Hydroxyethyl)piperazine-N-2-Ethane Sulfonic Acid buffer (HEPES, 10 mM), dithiothreitol (DTT, 1 mM), and ethylenediaminetetraacetic acid (EDTA, 0.5 mM). Subsequently, the tissues were further digested with an enzymatic solution consisting of RPMI 1640 medium, type IV collagenase (0.4 mg/mL), and DNase I. The digested tissues were filtered through a 200-mesh copper sieve to obtain a single-cell suspension, which was then centrifuged to collect the cell pellet. The colonic mononuclear cells were isolated by density gradient centrifugation using 40% percoll separation medium. Finally, the cells were resuspended in PBS for flow cytometry.

### Flow cytometry assay

Fluorochrome-conjugated antibodies were added to the cell suspension, mixed thoroughly and incubated at 4 °C for 30 min protected from light. After washing with PBS, the cells were resuspended in 200 μL PBS and mixed well. The antibodies for flow cytometry are shown in S2 Table.

### Histopathological, immunohistochemical (IHC) and immunofluorescence (IF) analyses

Distal colonic specimens were harvested and immersion-fixed in 4% paraformaldehyde for 24 h prior to paraffin embedding and microtome sectioning (4 μm).

Histopathological assessment was conducted through Hematoxylin and eosin (H&E) staining (Nanjing Jiancheng Bioengineering Institute, D006-1-4) to evaluate pathological changes and inflammatory cell infiltration. The grading of intestinal pathological lesions was determined by blind assessment based on three independent parameters: severity of inflammation (0–3); depth of injury (0–3); crypt damage (0–4) [49]. For further details, please refer to S3 Table. The mucus depletion scores based on Periodic Acid-Schiff (PAS, Beyotime, C0142M) and Alcian Blue (AB, Beyotime, C0155S) staining were determined by semiquantitatively assessing the extent of mucus depletion in the colonic mucosa. Mucus depletion was graded from 0 to 3: 0 (none), 1 (mild), 2 (moderate), 3 (severe). Higher scores indicated more extensive mucus depletion. Terminal Deoxynucleotidyl Transferase mediated dUTP Nick-End Labeling (TUNEL) Apoptosis Detection Kit (green fluorescence, Beyotime, C1088) was used to identify apoptotic cells.

For IHC and IF, deparaffinized sections underwent heat-mediated antigen retrieval in EDTA buffer (pH 8.0) followed by incubation with primary antibodies at 4 °C overnight. For IHC, colonic sections were incubated with HRP-conjugated secondary antibodies and visualized using 3,3′-diaminobenzidine (DAB). For IF, colonic sections were incubated with fluorophore-conjugated secondary antibodies and counterstained with DAPI. Antibody information is detailed in S4 Table.

Digital image capture system (Olympus, Tokyo, Japan) was used to capture sections. The intensity of immunohistochemistry was quantified using ImageJ software (National Institutes of Health, Bethesda, MD, USA). Specifically, the “Color Deconvolution” function (with the “H DAB” preset) was used to separate the DAB-positive signal from the hematoxylin counterstain, yielding a grayscale image representing the DAB signal. The same procedure was repeated for the channel representing all cells to obtain the total cell count. The positivity rate was calculated as: TIMD4-positive cell rate = (number of positive cells/total number of cells) × 100%. For IF analysis, mean fluorescence intensity was quantified using ImageJ software after background subtraction. Identical imaging and analysis parameters were applied to all groups.

### Transwell assay

BMDMs were generated by differentiating bone marrow-derived cells from WT and *Timd4* KO mice with M-CSF (50 ng/mL, ABclonal) for 5 days. For transwell migration assays, WT or *Timd4* KO BMDMs (1 × 10^5^ cells/well) were seeded in the lower chamber of 24-well Transwell plates (5 μm, Corning) and polarized with LPS (100 ng/mL; SIGMA) to establish inflammatory priming. Concurrently, WT BMDMs (1 × 10^5^ cells) were plated in serum-free medium within the upper inserts. Following a 20-h incubation (37 °C, 5% CO_2_), upper chambers were fixed in 4% paraformaldehyde (15 min, RT) and stained with aqueous crystal violet (15 min, RT).

Non-migrated cells on the inner surface of the upper chamber were carefully removed using cotton swabs, and excess stain was washed away with distilled water. Migrated cells were quantified by enumerating three randomly selected fields per membrane using microscope (Olympus, Tokyo, Japan).

### Quantitative real-time PCR

Total RNA was extracted from cell or tissue samples using TRNzol reagent (TIANGEN) and reverse transcribed to complementary DNA (cDNA) after removal of genomic DNA. Gene expression was detected by RT-qPCR using SYBR Green Master Mix on a StepOnePlus system. Relative gene expression was obtained using the comparative threshold cycle (2^−ΔΔCt^) method and normalized to β-actin. Primer sequences are shown in S5 Table.

### RNA sequencing

F4/80 ⁺ macrophages were isolated from WT and *Timd4* KO mice by collagenase digestion followed by magnetic separation using F4/80 antibody-conjugated magnetic beads (BioLegend, 480170). The isolated cells were collected, after which total RNA was extracted using TRIzol reagent (Invitrogen). Library construction and sequencing were performed by Beijing Genomics Institute using the DNBSEQ platform to generate 150-bp paired-end reads. Following quality filtering and adapter removal, clean reads were aligned to the mouse reference genome (mm10) using HISAT2 (v2.1.0), and gene expression was quantified using featureCounts (v2.0.1). Differential expression analysis was performed using DESeq2 (v1.30.1), with differentially expressed genes defined by |log_2_ fold change| > 1 and an adjusted *P* value < 0.05. KEGG pathway enrichment analysis was performed using a hypergeometric test implemented with the R function phyper. The resulting *P* values were adjusted for multiple testing using the Benjamini–Hochberg method, and pathways with an adjusted *P* value ≤ 0.05 were considered significantly enriched. Preranked GSEA was performed using predefined KEGG gene sets, with genes ranked according to their log_2_ fold-change values. Heatmaps were generated from Z-score-normalized FPKM values using Euclidean distance and complete-linkage hierarchical clustering. The RNA-seq data have been deposited into NCBI Sequence Read Archive (BioProject accession: PRJNA1271935).

### SDS-PAGE and western blotting

Cellular or tissue specimens were homogenized in RIPA lysis buffer (Beyotime, P0013D) in an ice bath for 30 min and subsequently subjected to high-speed centrifugation (12,000*g*, 15 min, 4 °C) to isolate protein-enriched supernatants. Cell membrane and cytoplasmic proteins were extracted using plasma membrane protein and cell component separation kit (Invent, SM-005). Protein concentrations were quantified using a bicinchoninic acid (BCA) assay, and samples were denatured by boiling in 5 × SDS loading buffer. Proteins were separated by SDS-PAGE and transferred onto polyvinylidene fluoride (PVDF) membranes. The membranes were incubated overnight at 4 °C with primary antibodies. Antibody details are provided in S6 Table. After washing, the membranes were incubated with HRP-coupled secondary antibodies, and protein bands were detected by enhanced chemiluminescence detection.

### Enzyme-linked immunosorbent assay

Colonic tissue samples (5 mg) were collected and homogenized in 200 μL of ice-cold PBS using a tissue homogenizer. The supernatants were carefully collected after centrifugation at 6,500*g* for 15 min at 4 °C. Cytokine concentrations, including IL-6, IL-1β and TNF-α, were quantified using commercially available ELISA kits (Mouse IL-6, BioLegend, 431304; Mouse IL-1β, BioLegend, 432604; Mouse TNF-α, BioLegend, 430904) according to the manufacturer’s protocols. Absorbance was measured using a microplate reader, and cytokine levels were determined based on standard curves generated for each assay.

### Structural modeling

Potential binding interactions among the TIMD4, MO25, STRAD, LKB1 proteins were examined using AlphaFold 3-based structural modeling. The amino acid sequences of TIMD4 (UniProtKB: Q96H15), MO25 (UniProtKB: Q9Y376), STRAD (UniProtKB: Q7RTN6) and LKB1 (UniProtKB: Q15831) were downloaded from the UniProt database (https://www.uniprot.org/), and AlphaFold 3 (https://alphafoldserver.com/about) was used for the prediction of protein molecular interactions and further selection of optimal protein structure models [50]. The optimal protein structure obtained was subsequently analyzed for amino acid residue interactions using Visual Studio Code software. Finally, the results were visualized by PyMOL software.

### Multicolor fluorescence analysis of human UC colon biopsy specimens

Human colonic tissue specimens were obtained from the Pathology Department of Qilu Hospital, comprising 4 non-colitis patients and 7 UC patients without age and gender preference. The UC cohort was further stratified based on the Ulcerative Colitis Endoscopic Severity Index (UCEIS) into 4 mild cases (UCEIS scores 2–4) and 3 moderately severe cases (UCEIS scores 5–6), with detailed clinical scores documented in S7 Table. Prior to specimen collection, written informed consent was obtained from all participants after comprehensive explanation of the study procedures. Multicolor immunofluorescence of biopsies was performed using the Opal Polaris 7 Color IHC Detection Kits (Akoya, OP-000003) according to the manufacturer’s instructions. Antibody information is detailed in S8 Table.

### Preparation of plasmids- and peptides-loaded nanoparticles

Plasmids/peptides-NPs were synthesized via DNA or peptide loading onto Hydroxyapatite (HAp) nanorods, followed by chemical coordination-mediated self-assembly of phenolic ligands and metal ions. Briefly, HAp NPs (1 mg) were dispersed in 1 mL of nuclease-free ddH_2_O via brief ultrasonication. Subsequently, 10 μL of plasmid P14 solution (Sequence: HA-eGFP-GGAGGG-GTGCAGCATGGAAGGGAAGACGAAGACGGCCTTTTTACCCTCTAA, 1 μg/μL, Tsingke Biotechnology) or T4_(365–378)_ peptide (Sequence: VQHGREDEDGLFTL, 0.1 mg, Dangang Biology) was introduced into the HAp suspension. After thorough mixing, the mixture was incubated at 4 °C for 5 min to enable HAp-plasmids/peptides interaction, yielding plasmids/peptides-HAp NPs. For Metal-Phenolic Network (MPN) coating, 3 μL of 24 mM tannic acid (TA, Sigma-Aldrich in Shanghai, China, 262897) and 3 μL of 60 mM ZnCl_2_ (Tianjin HEOWNS Biochemical Technology Co.) were added to the plasmids/peptides-HAp suspension, followed by mixing and incubation at 4 °C for 5 min. 3 μL of 40 mg/mL D-Mannose (Macklin in Shanghai, China, 103301-73-1) was added to the mixture, followed by incubation for 8 h. The resulting nanoparticles were washed three times with nuclease-free ddH_2_O and centrifuged (6,000 rpm, 5 min) to obtain P14/T4_(365–378)_-NPs.

### Characterization of plasmids-NPs

The morphology of the nanoparticles was analyzed using a transmission electron microscope (Hitachi, HT-7700, Japan). FTIR spectra were recorded using a FTIR spectrometer (Bruker Alpha-II, Germany) with a spectral resolution of 4 cm^−1^.

### Transfection efficiency of plasmids-NPs

The BMDMs were seeded in 6-well plates at a density of 2 × 10^6^ cells per well for 12 h, and incubated with 0.1 mg/mL plasmids-NPs. After 72 h, the cells were washed three times with PBS, and the transfection efficiency was assessed using fluorescence microscope.

### Statistical analysis

Data analysis and graphical representation were performed using GraphPad Prism (GraphPad Software, San Diego, CA, USA). All data are presented as mean ± SEM. Before conducting statistical comparisons, the Shapiro–Wilk test was first used to evaluate the normality of the data distribution. Based on the normality test results, parametric tests were applied to data that followed a normal distribution: the unpaired Student’s *t* test was employed to assess differences between two groups. When comparing more than two groups, one-way and two-way analysis of variance (ANOVA) were used, followed by post-hoc tests for intergroup comparisons. For data that did not follow a normal distribution, corresponding non-parametric tests (Mann–Whitney U test) were adopted. Survival curves were compared using the log-rank (Mantel–Cox) test. Statistical significance was reported as * *p* < 0.05, ** *p* < 0.01, *** *p* < 0.001 and ns, no significance.

## Supporting information

S1 Fig Identification of DSS-induced colitis. **(A)** Experimental protocol for DSS-induced colitis: WT mice received 3% DSS in drinking water for 7 days followed by 2-day recovery. **(B, C)** Gross morphology and statistics of colons from DSS-induced colitis and control mice. **(D)** Body weight change was assessed daily (*n* ≥ 3). **(E)** H&E staining of colonic tissues from DSS-induced colitis mice and control mice. → Inflammation, ☆ Edema, the black dotted lines highlight the crypt (Scale bars, 100 µm). All data are presented as mean ± SEM (*n* ≥ 3). The unpaired Student’s *t* test was employed to assess differences between two groups. Statistical significance was reported as * *p* < 0.05, ** *p* < 0.01. The data underlying this figure can be found in S1 Data.
(TIF)

S2 Fig *Timd4* deficiency aggravates DSS‑induced acute colitis in a 2% DSS model. **(A)** Experimental design: 6–8‑week‑old female C57BL/6J mice (WT and *Timd4* KO) were administered 2% DSS in drinking water for 7 days to induce acute colitis, followed by 2 days of regular drinking water. **(B)** Body weight changes were assessed daily. Statistics on the rate of weight loss on day 9. **(C)** DAI scores were calculated based on body weight loss, stool consistency and rectal bleeding. **(D)** Representative images and **(E)** quantitative analysis of colonic length. **(F)** Representative H&E staining images of colonic tissues. → Inflammation, ☆ Edema, the black dotted lines highlight the crypt. **(G)** Representative PAS staining and AB staining images of colonic tissue sections. **(H, I)** Representative immunofluorescence staining images of Mucin2, Claudin, Occludin and ZO‑1. Scale bar: 100 μm. **(J)** Histological scores based on H&E staining. **(K)** Statistical analysis of mucus depletion scores based on PAS and AB staining. **(L, M)** Quantification of mean fluorescence intensity for Mucin2, Claudin, Occludin and ZO‑1. Data are presented as mean ± SEM (*n* = 5). Statistical significance was assessed using two‑tailed unpaired Student’s *t*‑test for comparisons between two groups. * *p* < 0.05, ** *p* < 0.01. The data underlying this figure can be found in S1 Data.
(TIF)

S3 Fig *Timd4* deficiency aggravates DSS‑induced acute colitis in male mice. **(A)** Experimental design: 6–8‑week‑old male C57BL/6J mice (WT and *Timd4* KO) were administered 3% DSS in drinking water for 7 days to induce acute colitis, followed by 2 days of regular drinking water. **(B)** Body weight changes were assessed daily. Statistics on the rate of body weight loss on day 5–9. **(C)** DAI scores were calculated based on body weight loss, stool consistency and rectal bleeding. **(D)** Representative images and **(E)** quantitative analysis of colonic length. **(F)** ELISA measurement of pro-inflammatory cytokines (IL-6, IL-1β, TNF-α) in colonic tissue homogenates. **(G)** Representative H&E staining images of colonic tissues. → Inflammation, ☆ Edema, the black dotted lines highlight the crypt. **(H)** Representative PAS staining and AB staining images of colonic tissue sections. **(I, J)** Representative immunofluorescence staining images of Mucin2, Claudin, Occludin and ZO‑1. Scale bar: 100 μm. **(K)** Statistical analysis of histopathological scores. **(L)** Statistical analysis of mucus depletion scores based on PAS and AB staining. **(M, N)** Quantification of mean fluorescence intensity for Mucin2, Claudin, Occludin and ZO‑1. Data are presented as mean ± SEM (*n* = 4). Statistical significance was assessed using two‑tailed unpaired Student’s *t*‑test for comparisons between two groups. * *p* < 0.05, ** *p* < 0.01, *** *p* < 0.001. The data underlying this figure can be found in S1 Data.
(TIF)

S4 Fig The colonic morphology and histopathology of WT and *Timd4* KO mice under physiological state. **(A, B)** Colonic morphology of WT and *Timd4* KO mice in the healthy state. **(C)** Body weight change was assessed daily. **(D)** Representative H&E staining images of colonic tissues. **(E)** Representative images of PAS staining images and AB staining (Scale bars: 100 μm). **(F, G)** Immunofluorescence images of colonic tissue sections with **(F)** Mucin2, **(G)** Claudin, Occludin and ZO-1. Scale bars: 100 μm. Data are presented as mean ± SEM (*n* = 4). The unpaired Student’s *t* test was employed to assess differences between two groups. Statistical significance was reported as ns, no significance. The data underlying this figure can be found in S1 Data.
(TIF)

S5 Fig Flow gating scheme for evaluation of immune cells from colonic lamina propria. Flow gating scheme of **(A)** T cells and B cells and **(B)** Dendritic cells (DCs) and Monocyte-derived mononuclear phagocytes.
(TIF)

S6 Fig The expression of TIMD4 in immune cells in the DSS-induced colitis model. Flow cytometry analysis of the proportion of TIMD4^+^ cells and the mean fluorescence intensity of TIMD4 on different immune cell populations in colonic tissues under steady-state and colitic conditions. The changes of **(A, B)** dendritic cells, **(C, D)** T lymphocytes, **(E, F)** B lymphocytes were assessed. Data are presented as mean ± SEM (*n* = 4). The unpaired Student’s *t* test was employed to assess differences between two groups. Statistical significance was reported as ns, no significance. The data underlying this figure can be found in S1 Data.
(TIF)

S7 Fig KEGG pathway analysis of RNA-seq data. Resident macrophages from WT and *Timd4* KO mice were collected and subjected to RNA sequencing. KEGG pathway enrichment analysis was performed on the differentially expressed genes.
(TIF)

S8 Fig *Timd4* deficiency increases the expression of chemokines. **(A)** PEMs were obtained from the peritoneal cavity of mice. **(B)** BMDMs were obtained from bone marrow mononuclear cells induced by M-CSF in vitro. RNA was extracted after macrophages were stimulated for 24 h with LPS (100 ng/mL). Detection of chemokine expression by RT-qPCR. Data are presented as mean ± SEM (*n* = 3). Two-way ANOVA was employed to assess differences between multiple groups. Statistical significance was reported as * *p* < 0.05, ** *p* < 0.01, *** *p* < 0.001 and ns, no significance. The data underlying this figure can be found in S1 Data.
(TIF)

S9 Fig *Timd4* deficiency reduces CD206^+^ cell proportion and anti‑inflammatory gene expression in colonic tissues. **(A)** Representative flow cytometric plots of CD206^+^ cells gated on CD45^+^CD11b^+^CX3CR1^+^ cells. **(B)** Flow cytometric quantification of CD206⁺ cells. **(C)** mRNA levels of *Il10*, *Tgfb1* and *Arg1* in colonic tissues measured by RT-qPCR. Data are presented as mean ± SEM (*n* ≥ 3). The unpaired Student’s *t* test was employed to assess differences between two groups. Statistical significance was reported as * *p* < 0.05, ** *p* < 0.01. The data underlying this figure can be found in S1 Data.
(TIF)

S10 Fig TIMD4 inhibits NF-κB activation through the LKB1-AMPK axis. **(A)** GSEA analysis of RNA‑seq data from resident macrophages of WT and *Timd4* KO mice, showing enrichment of the NF‑κB signaling pathway in *Timd4* KO cells. **(B)** Representative immunoblots of phosphorylated P65 (p‑P65) and total P65 in colonic tissues from WT and *Timd4* KO mice. **(C)** Quantification of p‑P65/total P65 ratio. **(D)** Representative immunoblots of p‑LKB1, p‑AMPK and p‑P65 in WT and *Timd4* KO PEMs stimulated with increasing concentrations of LPS (0, 10, 50, 100 ng/mL) for 30 min. **(E)** Representative immunoblots of p‑LKB1, p‑AMPK and p‑P65 in WT and *Timd4* KO PEMs treated with or without the P65 inhibitor QNZ (10 μM, S4902, 1 h) prior to LPS stimulation (100 ng/mL, 30 min). **(F)** Representative immunoblots of p‑LKB1, p‑AMPK and p‑P65 in WT and *Timd4* KO PEMs treated with or without the AMPK inhibitor Compound C (10 μM, BML-275, 1 h) prior to LPS stimulation (100 ng/mL, 30 min). **(G–I)** Quantification of p‑LKB1, p‑AMPK and p‑P65 levels corresponding to the LPS dose‑response experiment shown in **(D)**. **(J–L)** Quantification of p‑LKB1, p‑AMPK and p‑P65 levels corresponding to the QNZ treatment experiment shown in **(E)**. **(M–O)** Quantification of p‑LKB1, p‑AMPK and p‑P65 levels corresponding to the Compound C treatment experiment shown in **(F)**. **(P)** Representative immunoblots of total LKB1, p‑LKB1, p‑AMPK and p‑P65 in WT and *Timd4* KO PEMs transfected with control siRNA or *Stk11* siRNA, followed by LPS stimulation (100 ng/mL, 30 min). **(Q–S)** Quantification of p‑LKB1, p‑AMPK and p‑P65 levels corresponding to the *Stk11* knockdown experiment shown in **(P)**. Data are presented as mean ± SEM (*n* = 3). The unpaired Student’s *t* test and two-way ANOVA were employed to assess differences between two and multiple groups. Statistical significance was reported as * *p* < 0.05, ** *p* < 0.01, *** *p* < 0.001 and ns, no significance. The data underlying this figure can be found in S1 Data.
(TIF)

S11 Fig TIMD4 regulates chemokine expression through the LKB1-AMPK-NF-κB axis. BMDMs from WT and *Timd4* KO mice were subjected to the indicated treatments, and mRNA expression levels of the chemokines *Ccl2*, *Ccl3*, *Ccl5*, *Ccl7* and *Ccl8* were quantified by RT-qPCR. **(A)** Effect of NF‑κB inhibition. Cells were pretreated with the NF‑κB inhibitor QNZ (EVP4593, 10 μM) for 1 h, followed by LPS stimulation (100 ng/mL) for 24 h. **(B)** Effect of AMPK inhibition. Cells were pretreated with the AMPK inhibitor Compound C (BML‑275, 10 μM) for 1 h, followed by LPS stimulation (100 ng/mL) for 24 h. **(C)** Effect of *Stk11* knockdown. Cells were transfected with *Stk11*‑specific siRNA or control siRNA for 24 h, then stimulated with LPS (100 ng/mL) for 24 h. Data are presented as mean ± SEM (*n* = 3). Two-way ANOVA was employed to assess differences between multiple groups. Statistical significance was reported as * *p* < 0.05, ** *p* < 0.01, *** *p* < 0.001 and ns, no significance. The data underlying this figure can be found in S1 Data.
(TIF)

S12 Fig The interaction of different domains of TIMD4 with the LKB1 complex. **(A)** Co-IP assays were performed using anti‑LKB1 antibody in HEK293T cells transfected with control vector or TIMD4 Δcyto. Representative Western blotting images of LKB1, MO25 and STRAD are shown. **(B)** Quantification of MO25 and STRAD protein levels in the co‑IP relative to LKB1. **(C)** Co-IP assays were performed using anti‑LKB1 antibody in HEK293T cells transfected with Vector or sTIMD4. Representative Western blotting images of LKB1, MO25 and STRAD. **(D)** Quantification of MO25 and STRAD protein levels in the IP relative to LKB1. **(E)** Co-IP assays were performed using anti‑LKB1 antibody in HEK293T cells transfected with Vector or P14. Representative Western blotting images of LKB1, MO25 and STRAD. **(F)** Quantification of MO25 and STRAD protein levels in the IP relative to LKB1. Data are presented as mean ± SEM (*n* = 3). The unpaired Student’s *t* test was employed to assess differences between two groups. Statistical significance was reported as * *p* < 0.05 and ns, no significance. The data underlying this figure can be found in S1 Data.
(TIF)

S13 Fig TIMD4 reduces LKB1 ubiquitination and proteasomal degradation. **(A)** Western blotting image of TIMD4 and LKB1 at 0, 6, 12, 18 and 24 h in HEK293T cells transfected with Vector or TIMD4 after treatment with CHX (0.5 μg/μL, Selleck, S7418). **(B)** Quantification of LKB1 degradation kinetics. Relative degradation rate was calculated as the ratio of LKB1 protein expression at each time point to the expression level at 0 h. **(C)** Western blotting image of ubiquitination of LKB1 in HEK293T cells transfected with Vector or TIMD4 after treatment with MG132 (20 μM, Beyotime, C1748) for 6 h. Data are presented as mean ± SEM (*n* = 3). The unpaired student’s *t* test was employed to assess differences between two groups. Statistical significance was reported as * *p* < 0.05 and ** *p* < 0.01. The data underlying this figure can be found in S1 Data.
(TIF)

S14 Fig The delivery of P14-NPs to macrophages alleviates colitis in *Timd4* KO mice. **(A)** The expression of chemokines (*Ccl2, Ccl3, Ccl5, Ccl7, Ccl8*) was detected by RT-qPCR in mouse colonic tissues after DSS treatment. **(B)** Colonic tissue homogenates were taken and the supernatants were assayed for IL-6, IL-1β and TNF-α levels by ELISA. **(C)** H&E staining to observe inflammatory cell infiltration and tissue damage. → Inflammation, ☆ Edema, the black dotted lines highlight the crypt. **(D)** Representative PAS and AB staining images of colonic sections. **(E, F)** Immunofluorescence images of colonic tissue sections with **(E)** Mucin2, **(F)** Claudin, Occludin and ZO-1 (Red). Scale bars: 100 μm. **(G)** Statistical analysis of histopathological scores. **(H)** Statistical analysis of mucus depletion scores based on PAS and AB staining. **(I, J)** Statistics on immunofluorescence intensity of (I) Mucin2, (J) Claudin, Occludin and ZO-1. Data are presented as mean ± SEM (*n* = 3). One-way ANOVA was employed to assess differences between multiple groups. Statistical significance was reported as * *p* < 0.05, ** *p* < 0.01, *** *p* < 0.001. The data underlying this figure can be found in S1 Data.
(TIF)

S15 Fig The delivery of P14-NPs to intestinal macrophages alleviates colitis in WT mice. **(A)** Daily monitoring of body weight changes. **(B)** Body weight, stool consistency and rectal bleeding were scored daily and summated to calculate the DAI scores (*n* = 4). **(C, D)** Quantitative analysis of colonic length on day 9 (*n* = 4). **(E, F)** Western blotting analysis of total P65 and p-P65 in colonic lysates. **(G)** RT-qPCR quantification of chemokine expression (*Ccl2, Ccl3, Ccl5, Ccl7, Ccl8*) in colonic tissues. **(H)** ELISA quantification of pro-inflammatory cytokines (IL-6, IL-1β, TNF-α) in colonic tissue homogenates (*n* = 4). **(I)** Representative H&E staining images of colonic tissues. → Inflammation, ☆ Edema, the black dotted lines highlight the crypt. **(J)** Representative PAS staining images and AB staining images. **(K, L)** Representative immunofluorescence staining images of Mucin2, Claudin, Occludin and ZO-1. Scale bars: 100 μm. **(M)** Statistical analysis of histopathological scores. **(N)** Statistical analysis of mucus depletion scores based on PAS and AB staining. **(O, P)** Statistics on immunofluorescence intensity of **(O)** Mucin2, **(P)** Claudin, Occludin and ZO-1. Data are presented as mean ± SEM (*n* = 4). One-way ANOVA was employed to assess differences between multiple groups. Statistical significance was reported as * *p* < 0.05, ** *p* < 0.01, *** *p* < 0.001. The data underlying this figure can be found in S1 Data.
(TIF)

S16 Fig T4_(365–378)_-NPs exhibit therapeutic efficacy comparable to CCR2 antagonist in acute DSS-induced colitis. **(A)** Representative H&E staining images of colonic tissues. → Inflammation, ☆ Edema, the black dotted lines highlight the crypt. **(B)** Representative PAS staining images and AB staining images. **(C, D)** Representative immunofluorescence staining images of Mucin2, Claudin, Occludin and ZO-1. Scale bars: 100 μm. **(E)** Statistical analysis of histopathological scores. **(F)** Statistical analysis of mucus depletion scores based on PAS and AB staining. **(G, H)** Quantification of mean fluorescence intensity of Mucin2, Claudin, Occludin and ZO-1. Data are presented as mean ± SEM (*n* = 5). One-way ANOVA was employed to assess differences between multiple groups. Statistical significance was reported as * *p* < 0.05, ** *p* < 0.01, *** *p* < 0.001 and ns, no significance. The data underlying this figure can be found in S1 Data.
(TIF)

S17 Fig Prophylactic administration of T4_(365–378)_-NPs ameliorates DSS‑induced acute colitis in mice. **(A)** Representative H&E staining images of colonic tissues. → Inflammation, ☆ Edema, the black dotted lines highlight the crypt. **(B)** Representative PAS staining images and AB staining images. **(C, D)** Representative immunofluorescence staining images of Mucin2, Claudin, Occludin and ZO-1. Scale bars: 100 μm. **(E)** Statistical analysis of histopathological scores. **(F)** Statistical analysis of mucus depletion scores based on PAS and AB staining. **(G, H)** Quantification of mean fluorescence intensity of Mucin2, Claudin, Occludin and ZO-1. Data are presented as mean ± SEM (*n* = 5). One-way ANOVA was employed to assess differences between multiple groups. Statistical significance was reported as * *p* < 0.05, ** *p* < 0.01, *** *p* < 0.001. The data underlying this figure can be found in S1 Data.
(TIF)

S18 Fig Combination treatment with T4_(365‑378)_-NPs and anti‑α4β7 antibody during the active phase of DSS-induced colitis. **(A)** Experimental protocol. Female C57BL/6J mice (6–8 weeks old) were randomly assigned to six groups: Water group (normal drinking water for 9 days); DSS group (3% DSS for 7 days, followed by regular drinking water for 2 days); anti‑α4β7 group (clone DATK32, intraperitoneal injection, 50 μg, on days 4 and 7); Isotype control IgG group (rat IgG2a, intraperitoneal injection, 50 μg, on days 4 and 7); T4_(365‑378)_-NPs group (intrarectal administration, 0.1 mg, days 4 and 7); Combination group (anti‑α4β7 + T4_(365‑378)_-NPs according to the above regimens). **(B)** Body weight changes were assessed daily. Statistics on the rate of body weight loss on day 9. **(C)** Representative images and **(D)** quantitative analysis of colonic length. **(E)** Body weight, stool consistency and rectal bleeding were scored and summated to calculate the DAI scores. **(F)** Representative H&E staining images of colonic tissues. → Inflammation, ☆ Edema, the black dotted lines highlight the crypt. **(G)** Representative PAS staining images and AB staining images. **(H, I)** Representative immunofluorescence staining images of Mucin2, Claudin, Occludin and ZO-1. Scale bars: 100 μm. **(J)** Statistical analysis of histopathological scores. **(K)** Statistical analysis of mucus depletion scores based on PAS and AB staining. **(L, M)** Quantification of mean fluorescence intensity of Mucin2, Claudin, Occludin and ZO-1. Data are presented as mean ± SEM (*n* = 5). One-way ANOVA was employed to assess differences between multiple groups. Statistical significance was reported as * *p* < 0.05, ** *p* < 0.01, *** *p* < 0.001 and ns, no significance. The data underlying this figure can be found in S1 Data.
(TIF)

S1 Table DAI standardized scoring system. (XLSX)

S2 Table Detailed information of flow cytometry antibodies. (XLSX)

S3 Table Intestinal histopathology score. (XLSX)

S4 Table Detailed information of IHC and IF antibodies. (XLSX)

S5 Table Primer Sequences. (XLSX)

S6 Table Detailed information of Western blotting antibodies. (XLSX)

S7 Table UCEIS score of the patient biopsy specimens. (XLSX)

S8 Table Detailed information of multicolor immunofluorescence antibodies. (XLSX)

S1 Raw Images Uncropped original images. (PDF)

S1 Data Source data underlying Figures and Supplementary Figures. (XLSX)

## Abbreviations

AMPK: **Adenosine 5’-monophosphate (AMP)-**activated protein kinase
BCA: bicinchoninic acid
BMDMs: bone marrow-derived macrophages
CD: Crohn’s disease
CLs: clodronate liposomes
DAI: disease activity index
DCs: Dendritic cells
DSS: dextran sulfate sodium
FBS: fetal bovine serum
FTIR: Fourier-transform infrared
GEO: Gene Expression Omnibus
GO: Gene Ontology
GSEA: Gene Set Enrichment Analysis
IBD: Inflammatory bowel disease
KEGG: Kyoto Encyclopedia of Genes and Genomes
LKB1: liver kinase B1
MPN: Metal-Phenolic Network
PEMs: peritoneal macrophages
TUNEL: Transferase deoxynucleotidyl transferase-mediated dUTP Nick-End Labeling
UC: ulcerative colitis
WCL: whole-cell lysate
