# Supplementary material for: A macrophage-targeted TIMD4 Peptide-Nanoparticle attenuates colitis symptoms and monocyte recruitment in a mouse model
Source: PLoS Biol. 2026 Oct 21;24(10):e3004056. doi: 10.1371/journal.pbio.3004056 (PMC13686490; doi:10.1371/journal.pbio.3004056)

Fig. 1C

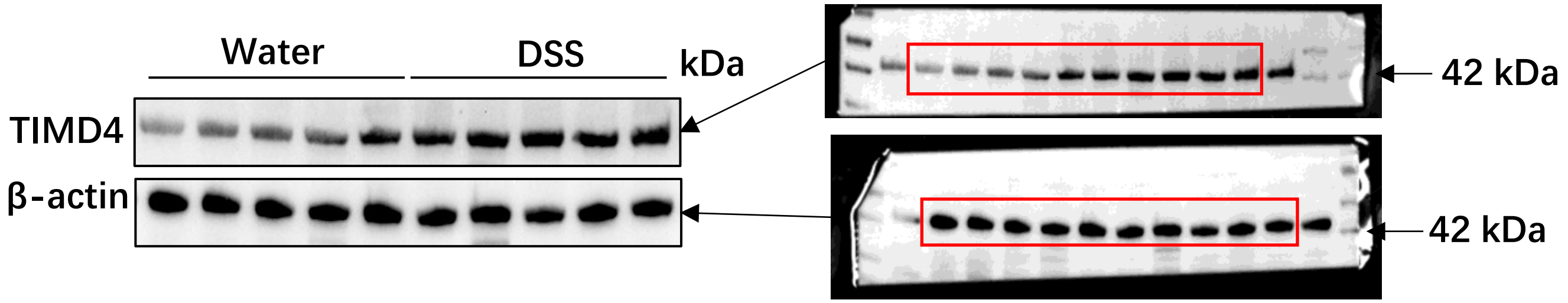
Water
DSS
kDa
TIMD4
β-actin
42 kDa
42 kDa

Fig. 1J

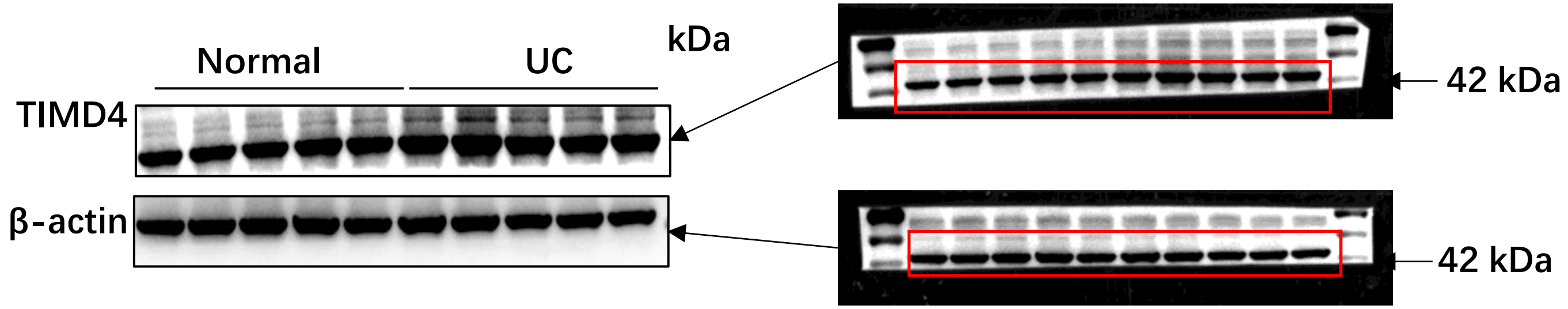
Normal
UC
kDa
TIMD4
β-actin
42 kDa
42 kDa

Fig. 5A

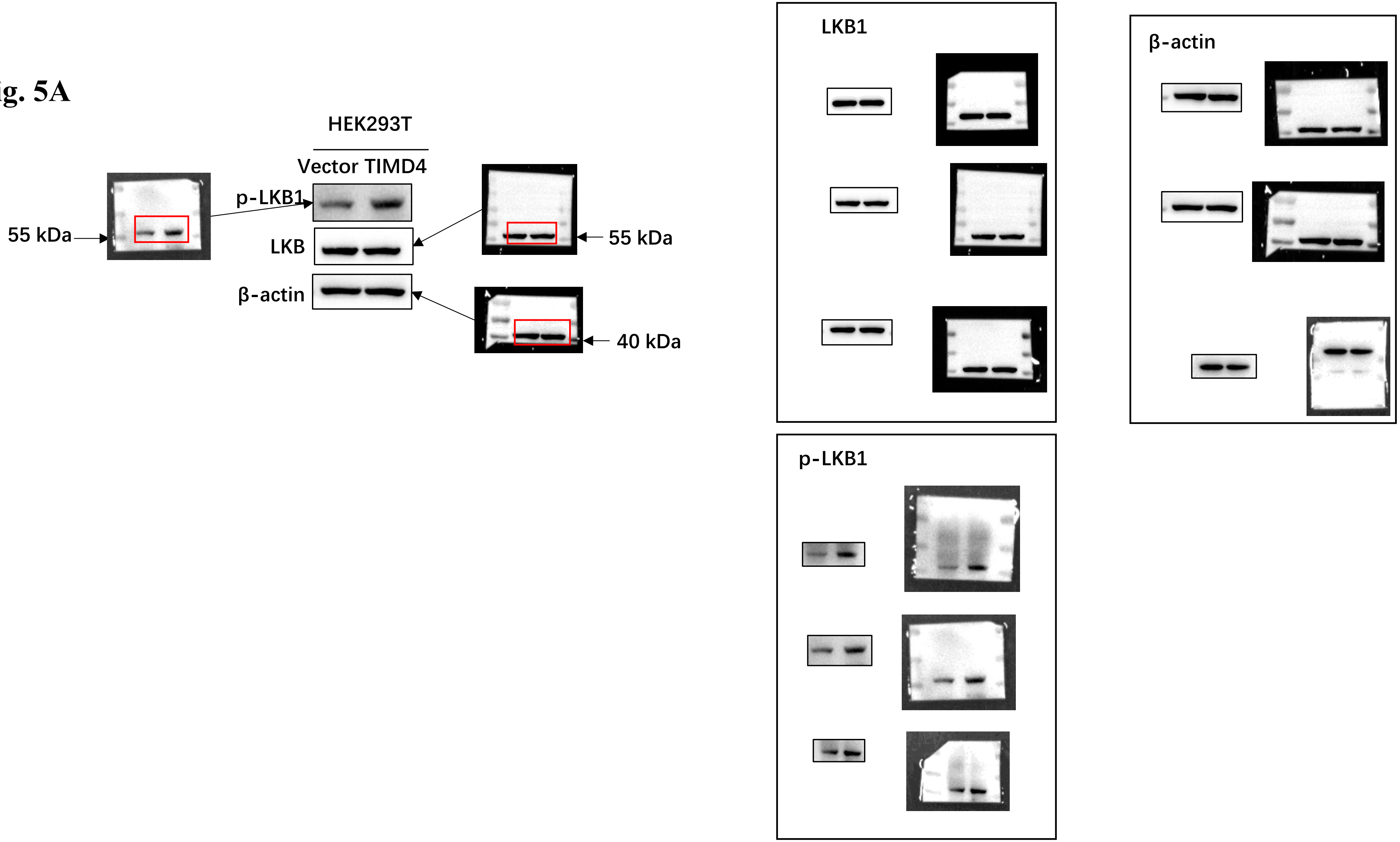

HEK293T
Vector TIMD4
p-LKB1
LKB
β-actin
55 kDa
55 kDa
40 kDa
LKB1
β-actin
p-LKB1

Fig. 5C

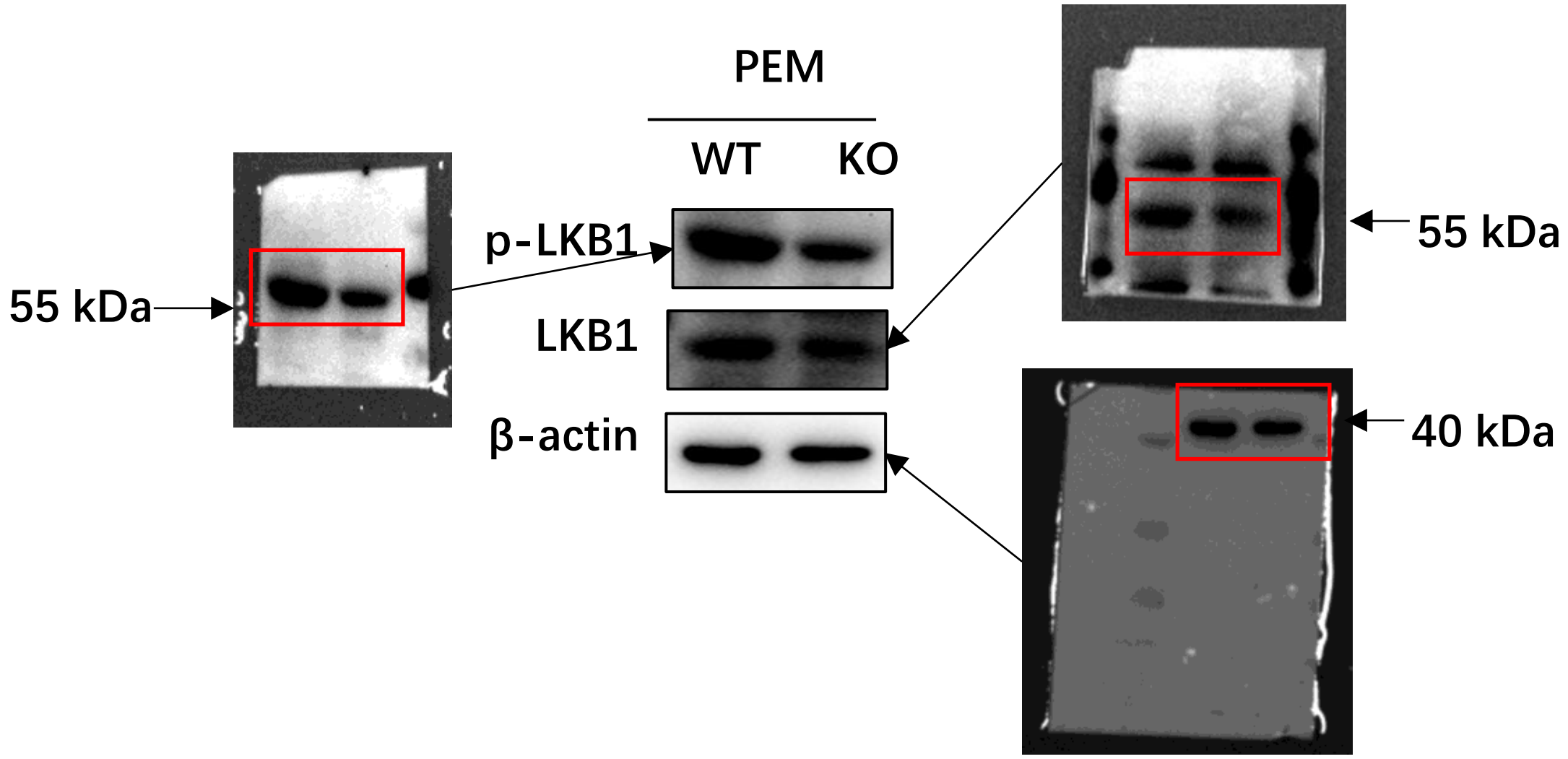
PEM
WT
KO
55 kDa
p-LKB1
LKB1
β-actin
55 kDa
40 kDa

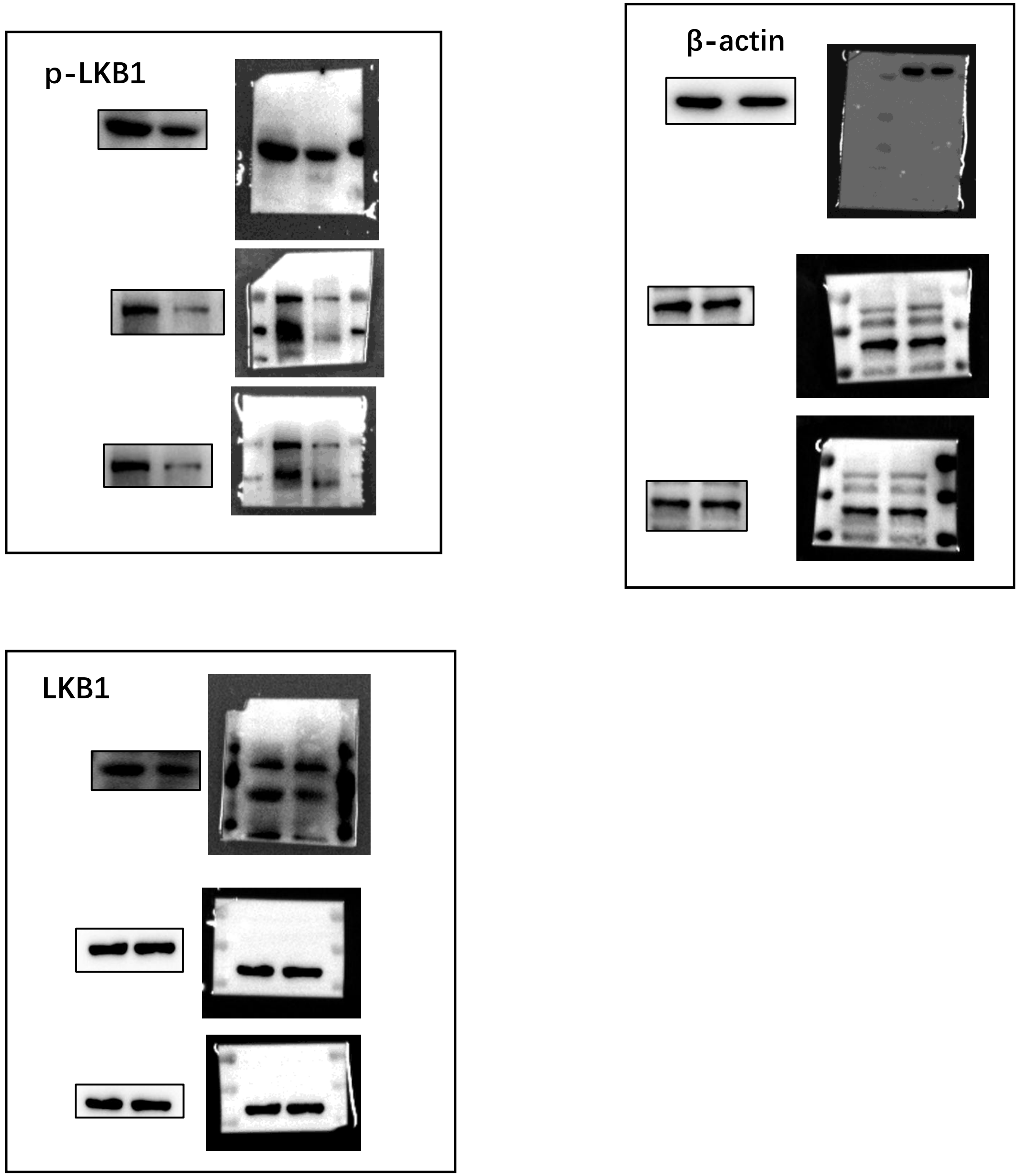
p-LKB1
β-actin
LKB1

Fig. 5F

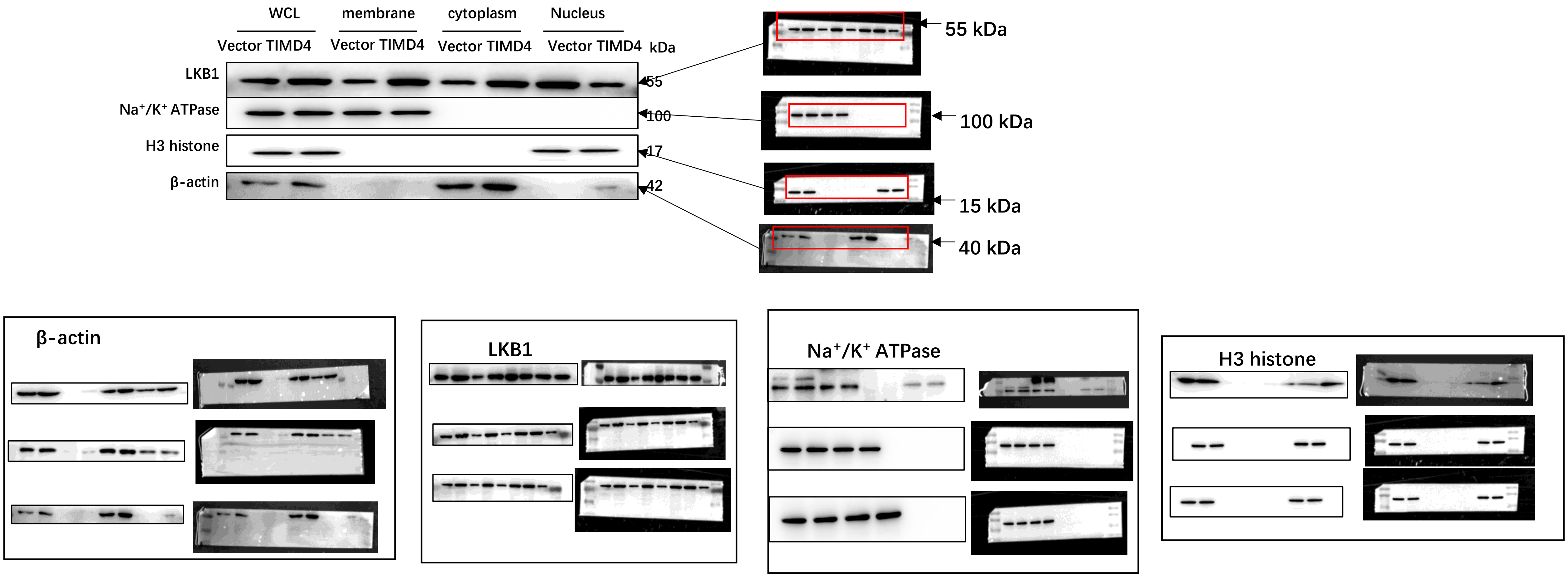
WCL
membrane
cytoplasm
Nucleus
Vector TIMD4
Vector TIMD4
Vector TIMD4
Vector TIMD4
kDa
LKB1
Na+/K+ ATPase
H3 histone
β-actin
55
100
17
42
55 kDa
100 kDa
15 kDa
40 kDa
β-actin
LKB1
Na+/K+ ATPase
H3 histone

Fig. 5L

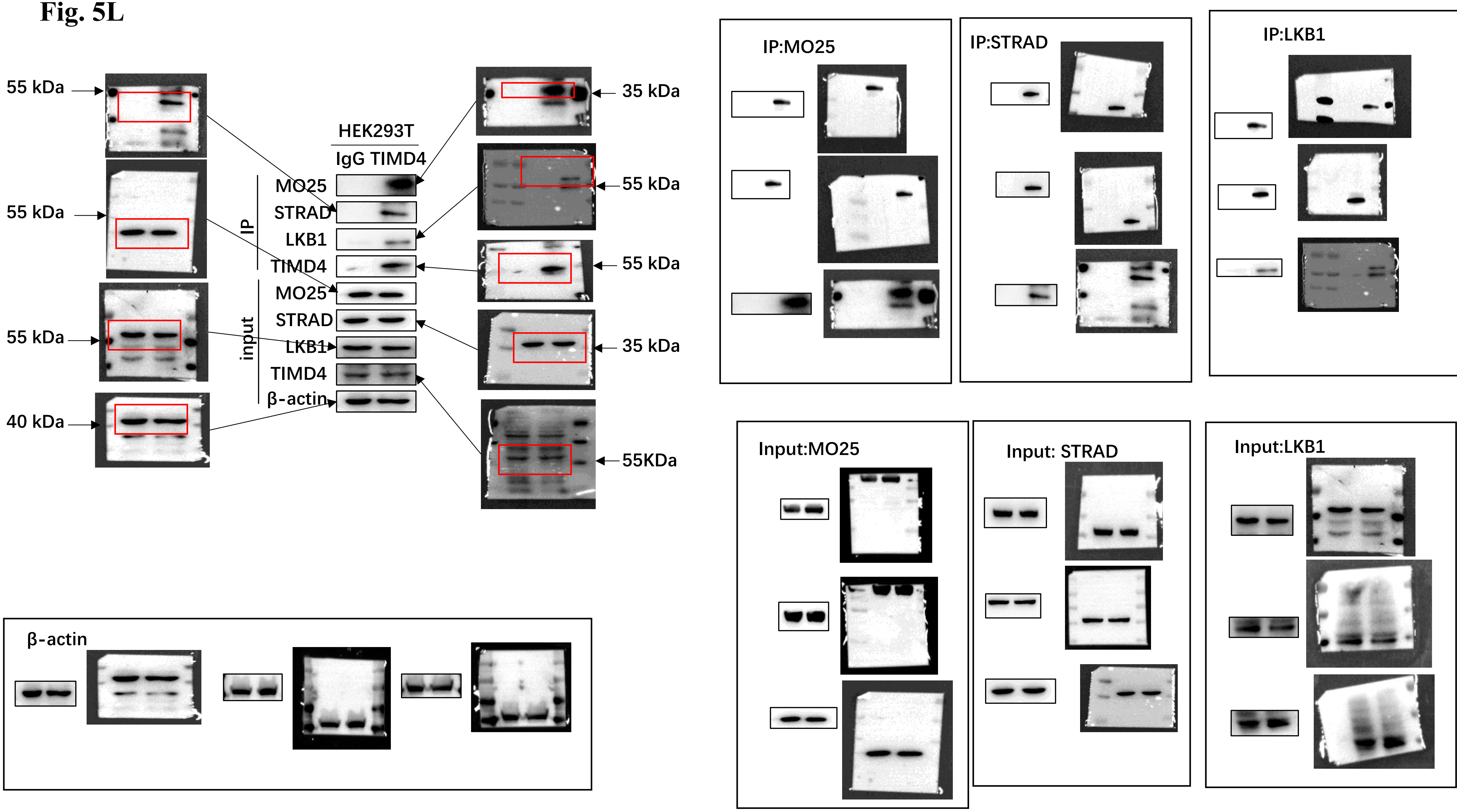
55 kDa
55 kDa
55 kDa
40 kDa
HEK293T
IgG TIMD4
IP
MO25
STRAD
LKB1
TIMD4
input
MO25
STRAD
LKB1
TIMD4
β-actin
35 kDa
55 kDa
55 kDa
35 kDa
55KDa
β-actin
IP:MO25
IP:STRAD
IP:LKB1
Input:MO25
Input: STRAD
Input:LKB1

Fig. 5M

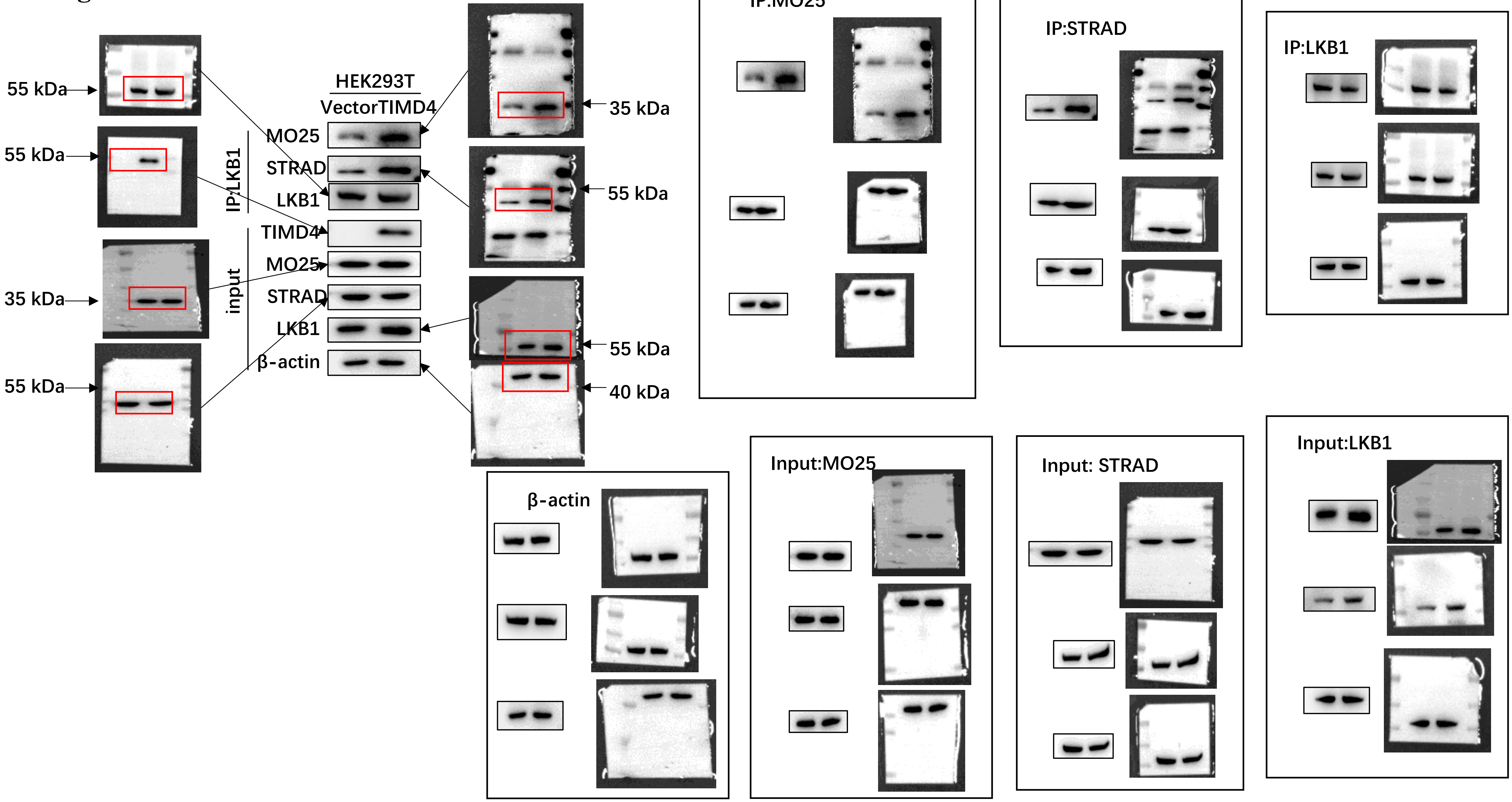

55 kDa
55 kDa
35 kDa
55 kDa
HEK293T
Vector TIMD4
IP:LKB1
MO25
STRAD
LKB1
input
TIMD4
MO25
STRAD
LKB1
β-actin
35 kDa
55 kDa
55 kDa
40 kDa
IP:MO25
IP:STRAD
IP:LKB1
β-actin
Input:MO25
Input: STRAD
Input:LKB1

Fig. 5O

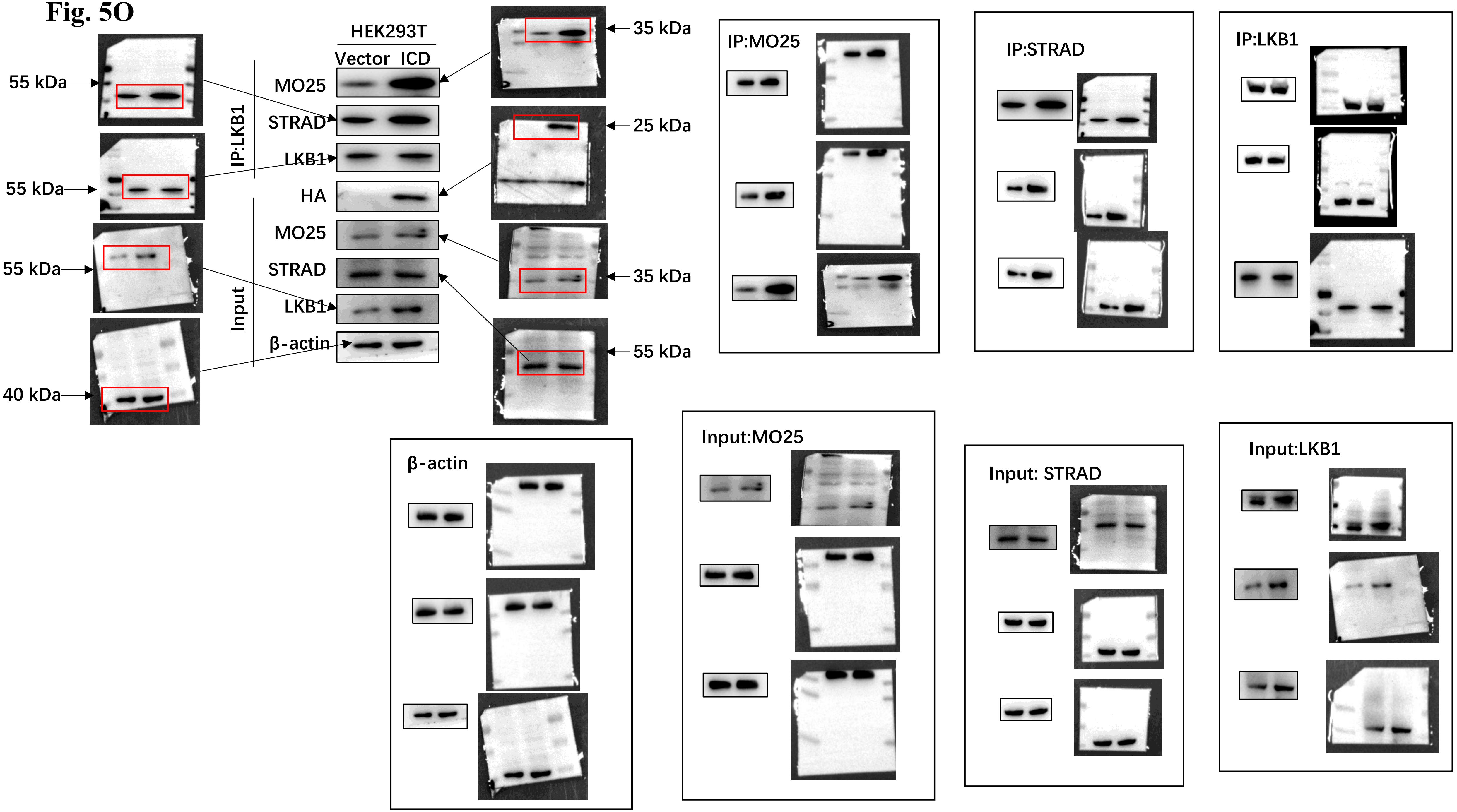

HEK293T
Vector ICD
IP:LKB1
MO25
STRAD
LKB1
HA
Input
MO25
STRAD
LKB1
β-actin
55 kDa
55 kDa
55 kDa
40 kDa
35 kDa
25 kDa
35 kDa
55 kDa
IP:MO25
IP:STRAD
IP:LKB1
β-actin
Input:MO25
Input: STRAD
Input:LKB1

Fig. 5Q

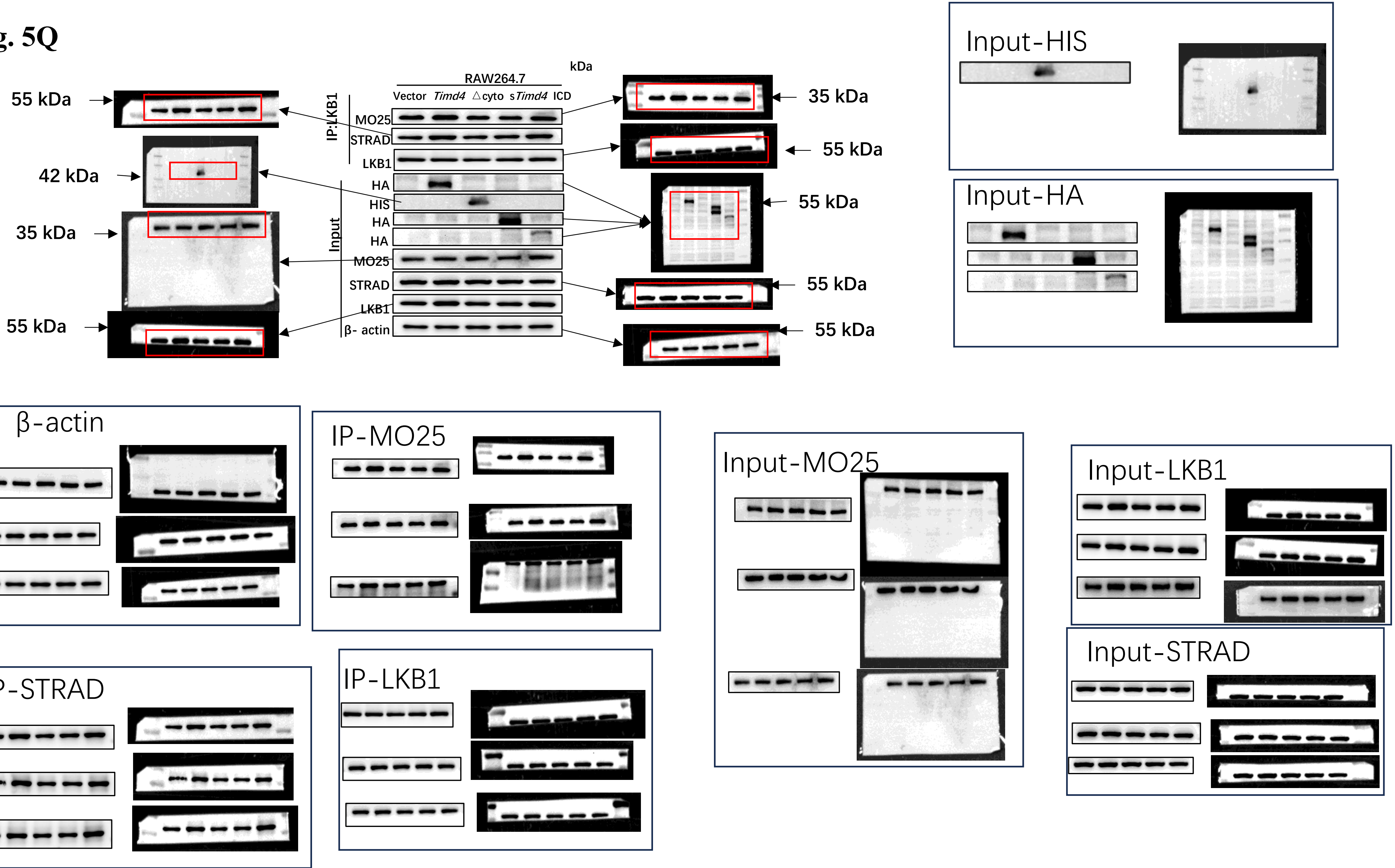

RAW264.7
kDa
Vector
Timd4
△cyto
sTimd4
ICD
IP:LKB1
MO25
STRAD
LKB1
Input
HA
HIS
HA
HA
MO25
STRAD
LKB1
β- actin
55 kDa
42 kDa
35 kDa
55 kDa
35 kDa
55 kDa
55 kDa
55 kDa
55 kDa
Input-HIS
Input-HA
β-actin
IP-MO25
Input-MO25
Input-LKB1
Input-STRAD
IP-STRAD
IP-LKB1

Fig. 6E

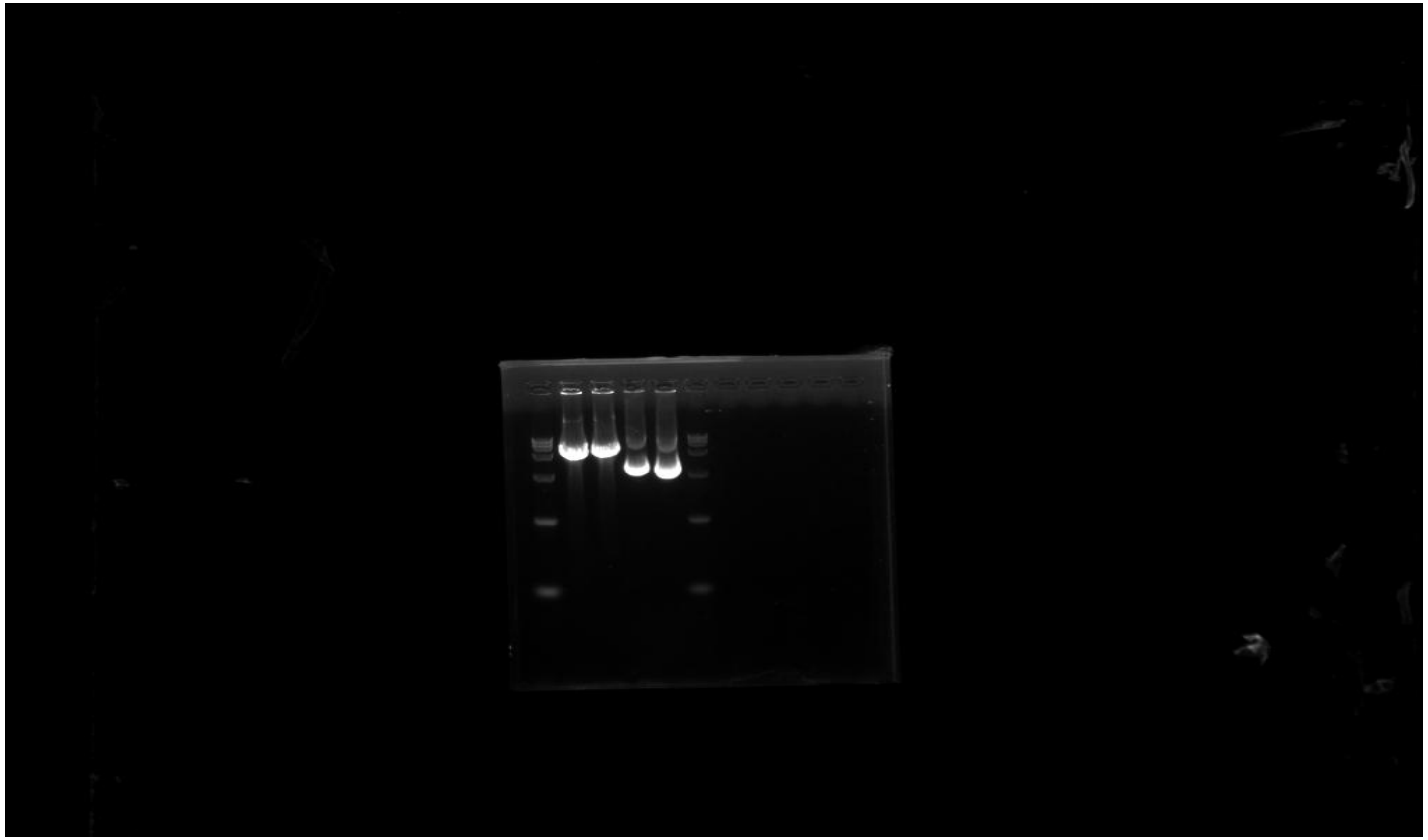

Fig. 6G

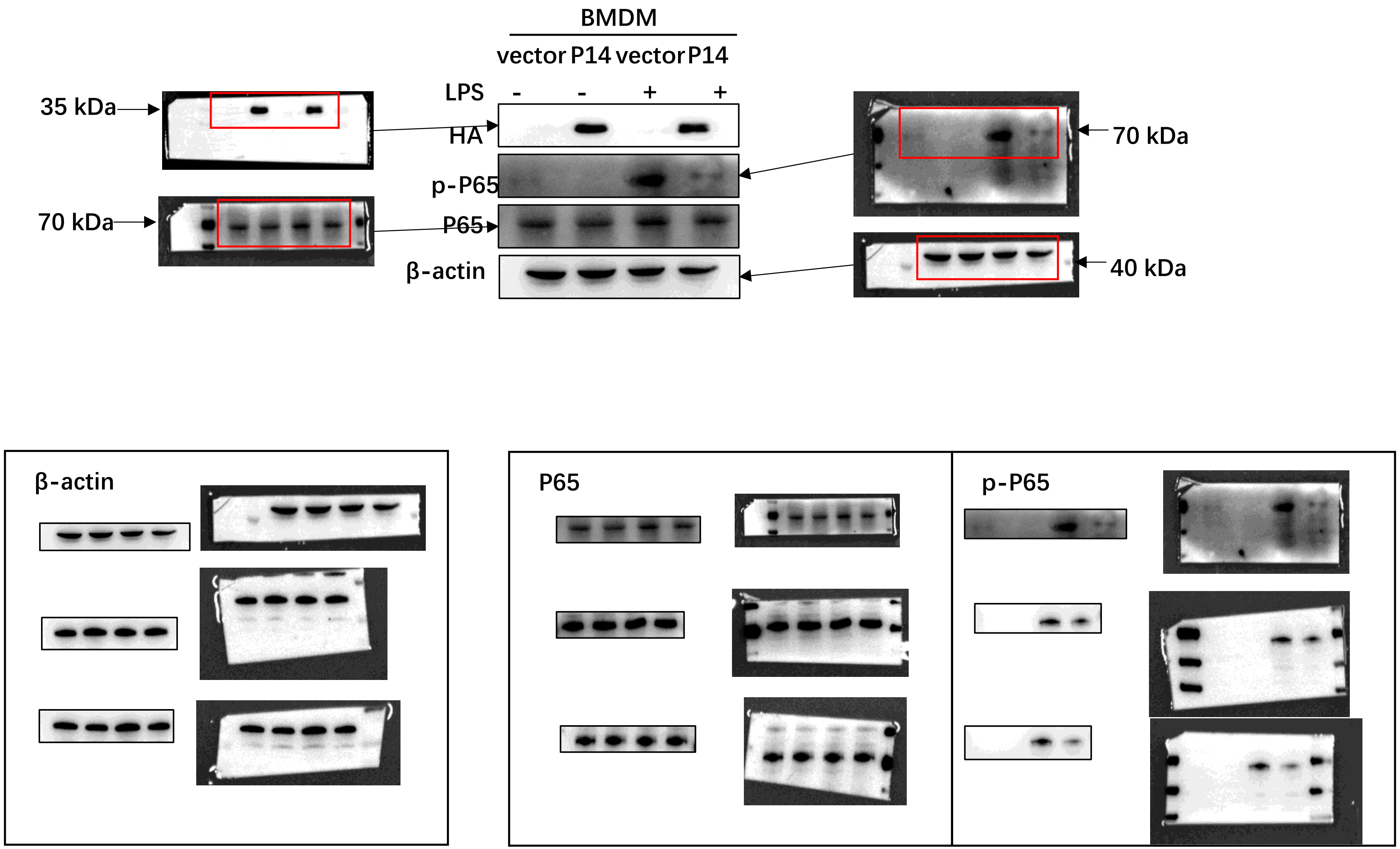

BMDM
vector P14 vector P14
LPS - - + +
HA
p-P65
P65
β-actin
35 kDa
70 kDa
70 kDa
40 kDa
β-actin
P65
p-P65

Fig. 6K

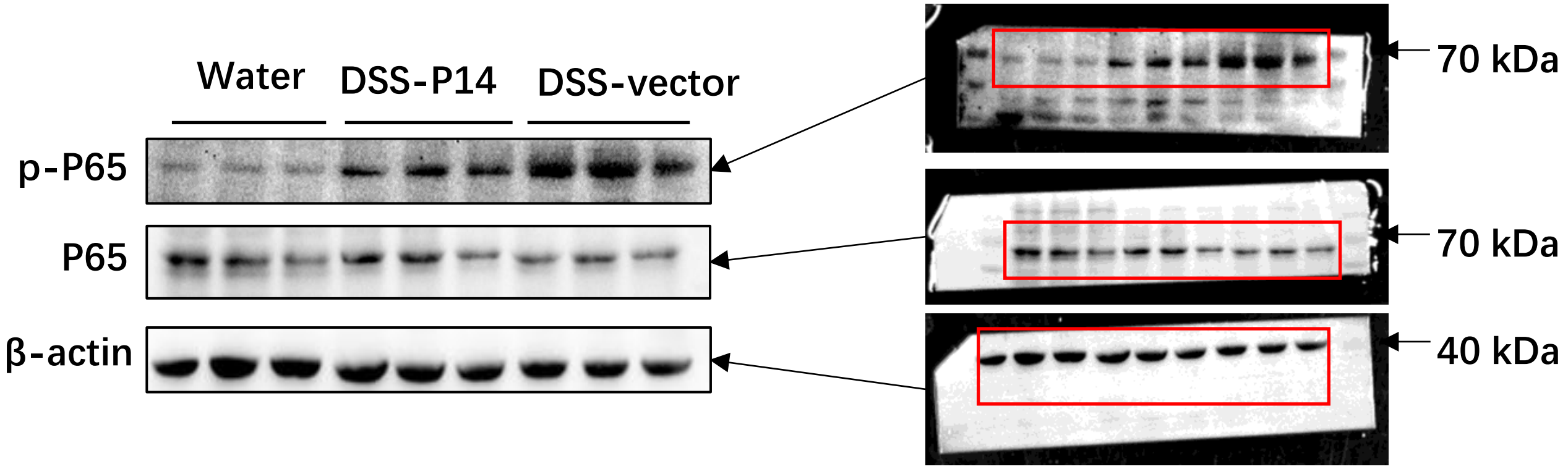

Fig. 7F

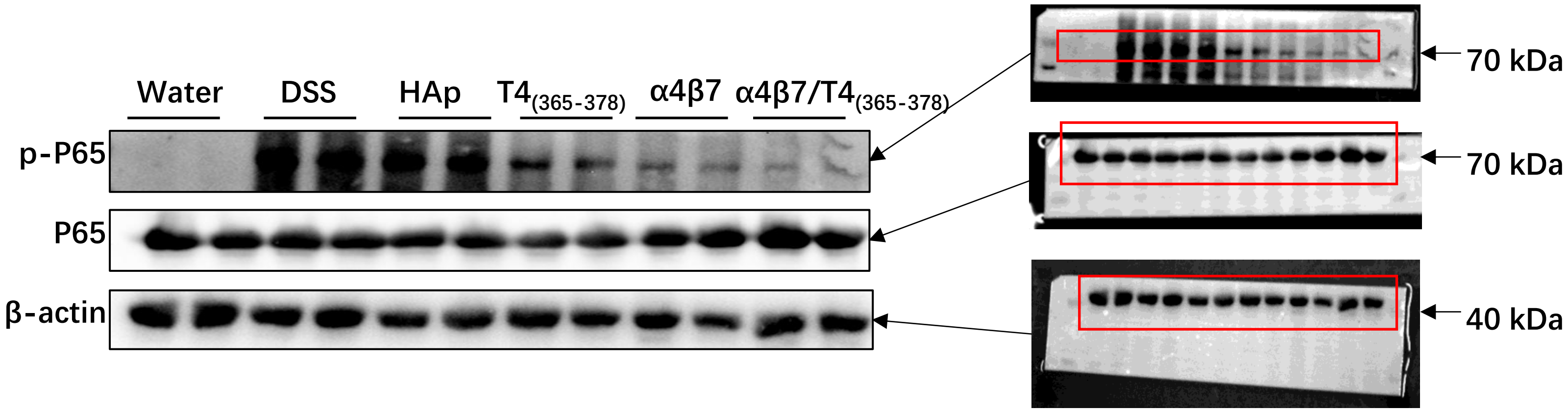
Water
DSS
HAp
T4(365-378)
α4β7
α4β7/T4(365-378)
p-P65
P65
β-actin
70 kDa
70 kDa
40 kDa

Fig. S10B

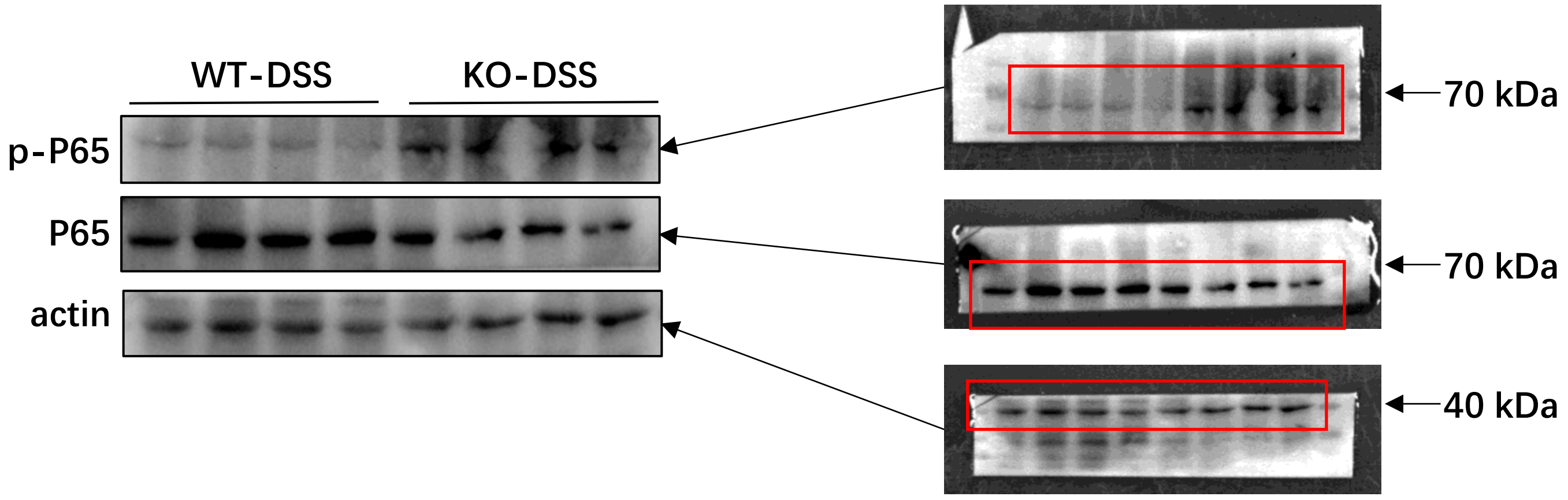

Fig. S10D

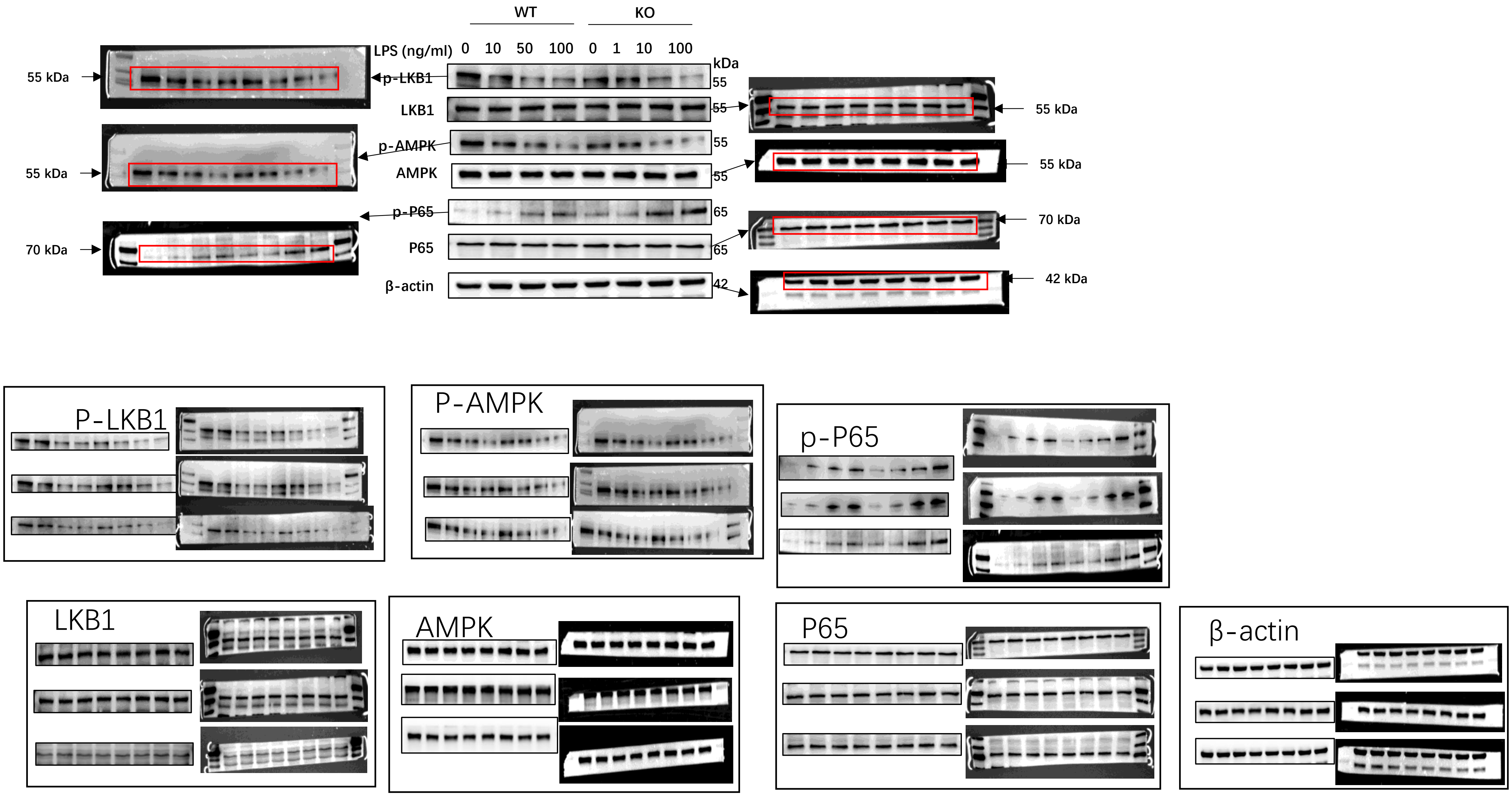
WT
KO
LPS (ng/ml) 0 10 50 100 0 1 10 100
kDa
p-LKB1
LKB1
p-AMPK
AMPK
p-P65
P65
β-actin
55
55
55
55
65
65
42
55 kDa
55 kDa
70 kDa
55 kDa
55 kDa
70 kDa
42 kDa
P-LKB1
P-AMPK
p-P65
LKB1
AMPK
P65
β-actin

Fig. S10E

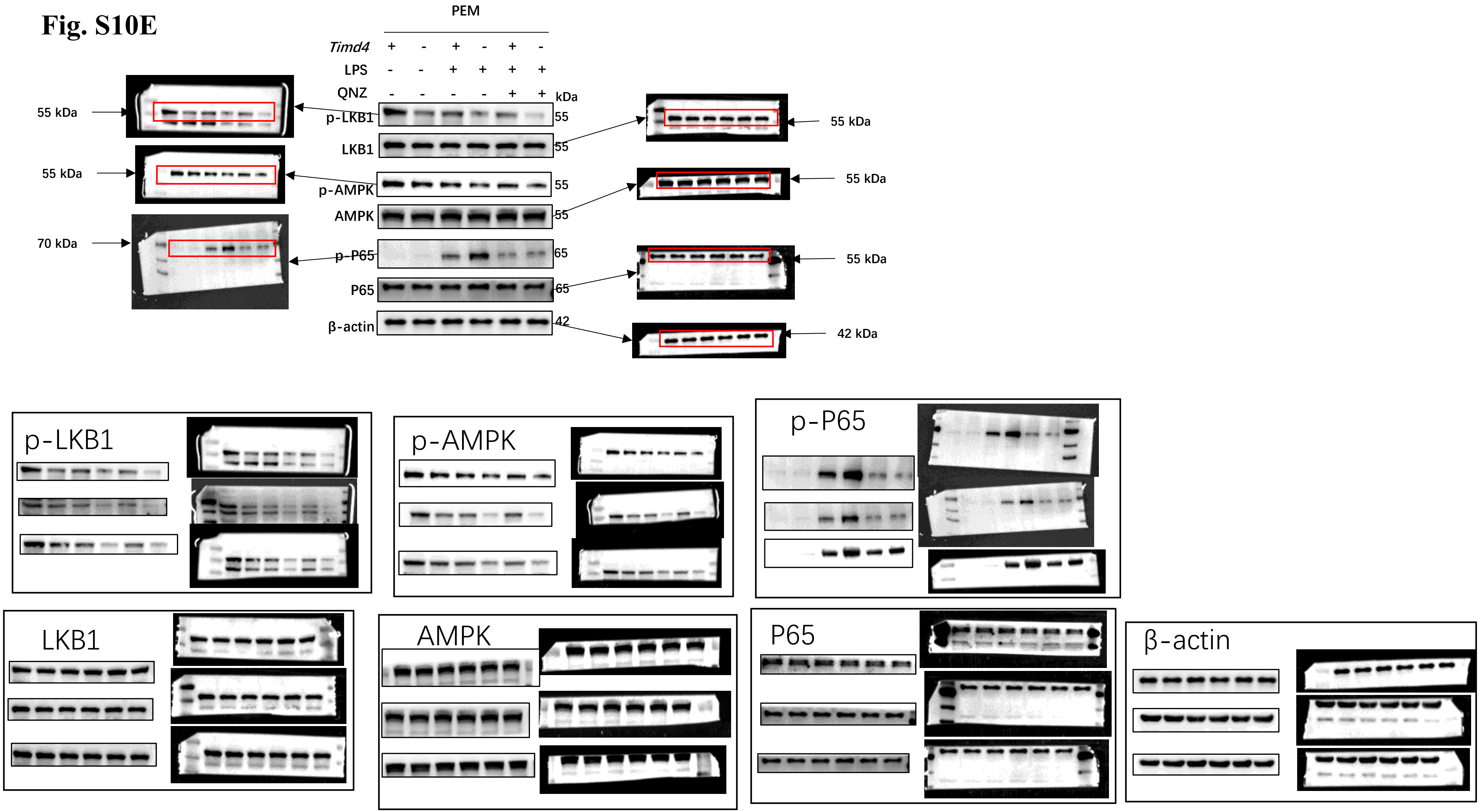
PEM
Timd4 + - + - + -
LPS - - + + + +
QNZ - - - - + +
kDa
p-LKB1 55
LKB1 55
p-AMPK 55
AMPK 55
p-P65 65
P65 65
β-actin 42
55 kDa
55 kDa
70 kDa
55 kDa
55 kDa
55 kDa
42 kDa
p-LKB1
p-AMPK
p-P65
LKB1
AMPK
P65
β-actin

Fig. S10F

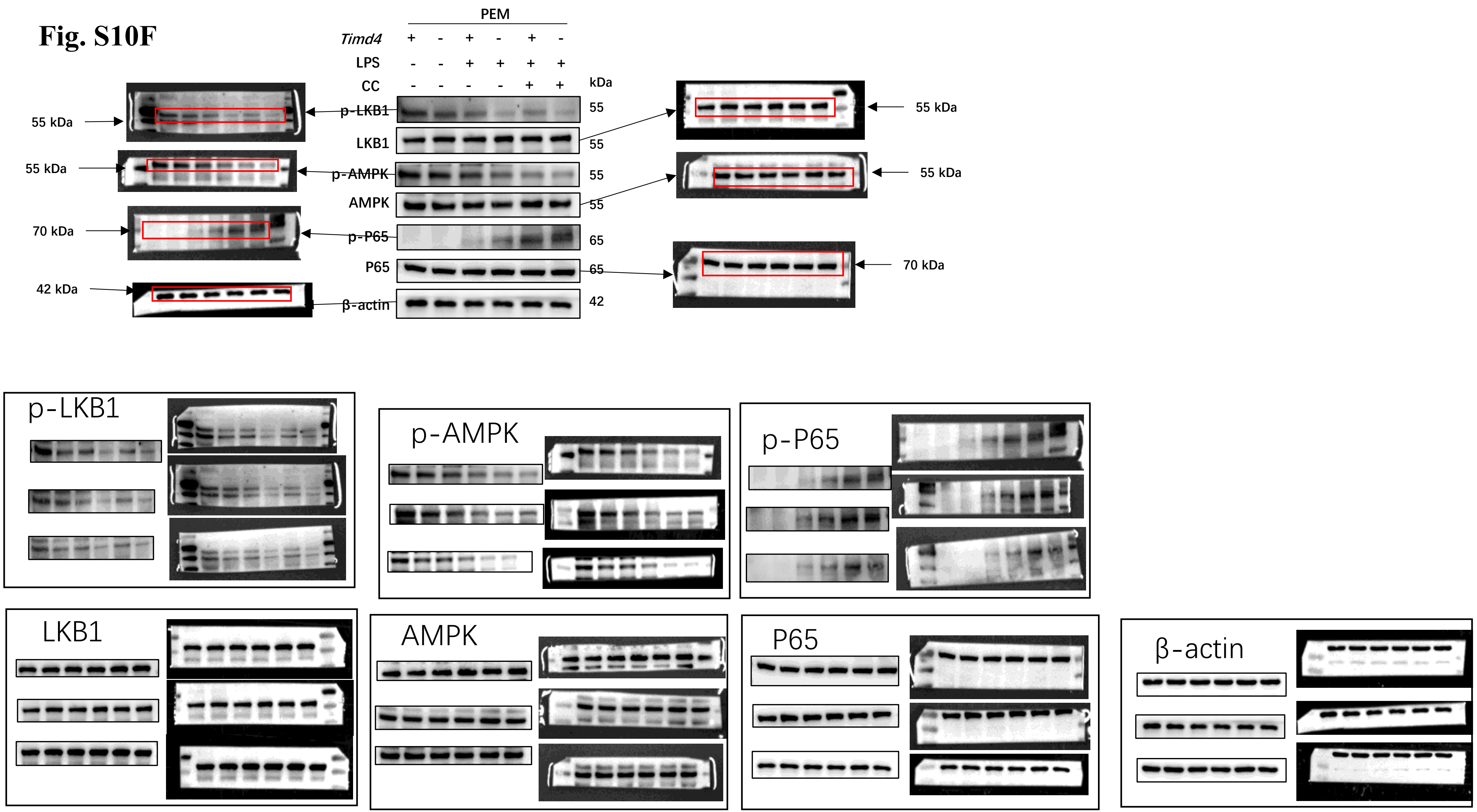
PEM
Timd4 + - + - + -
LPS - - + + + +
CC - - - - + +
kDa
p-LKB1 55
LKB1 55
p-AMPK 55
AMPK 55
p-P65 65
P65 65
β-actin 42
55 kDa
55 kDa
70 kDa
42 kDa
55 kDa
55 kDa
70 kDa
p-LKB1
p-AMPK
p-P65
LKB1
AMPK
P65
β-actin

Fig. S10P

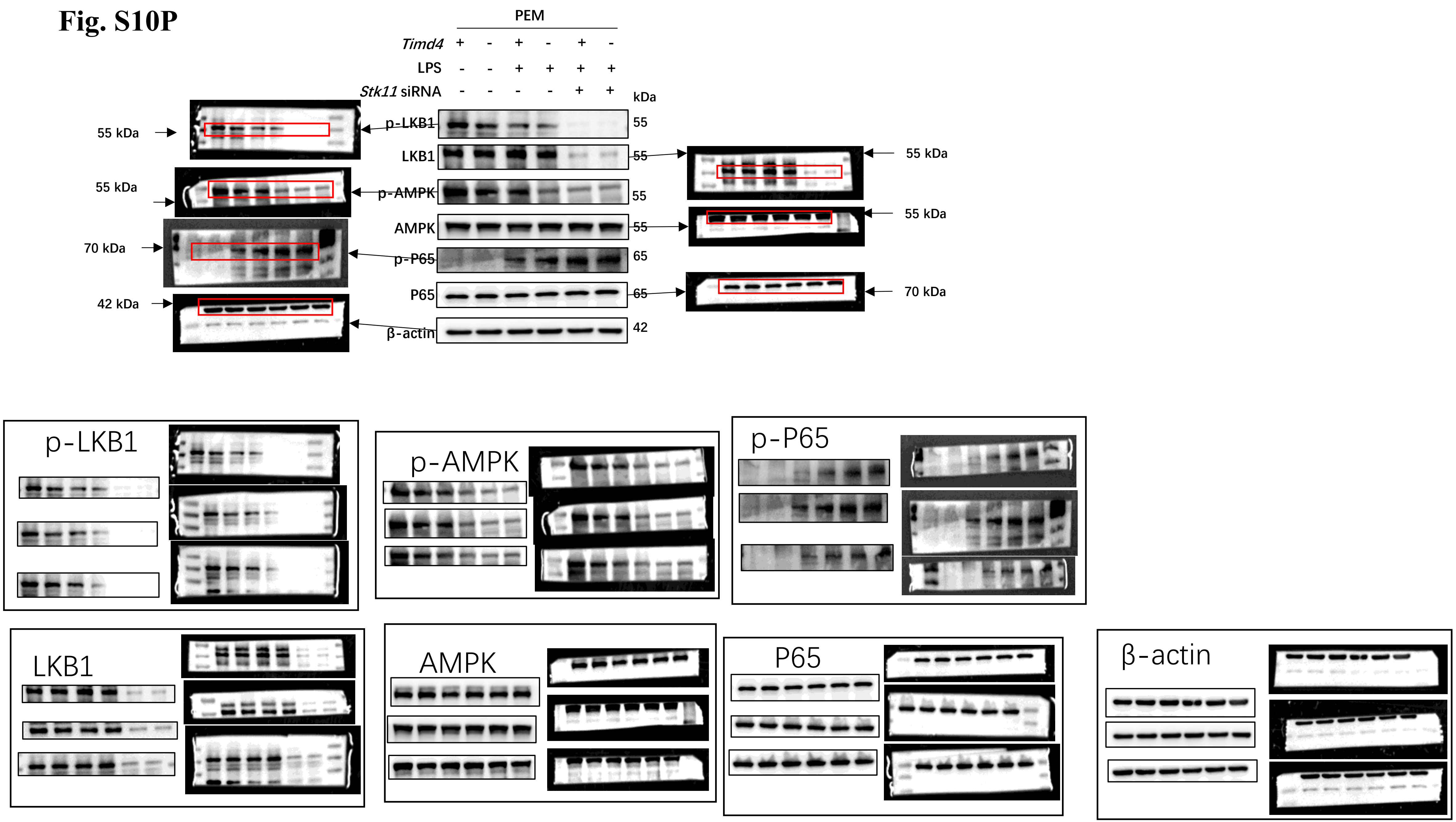
PEM
Timd4 + - + - + -
LPS - - + + + +
Stk11 siRNA - - - - + +
kDa
p-LKB1 55
LKB1 55
p-AMPK 55
AMPK 55
p-P65 65
P65 65
β-actin 42
55 kDa
55 kDa
70 kDa
42 kDa
55 kDa
55 kDa
70 kDa
p-LKB1
p-AMPK
p-P65
LKB1
AMPK
P65
β-actin

Fig. S12A

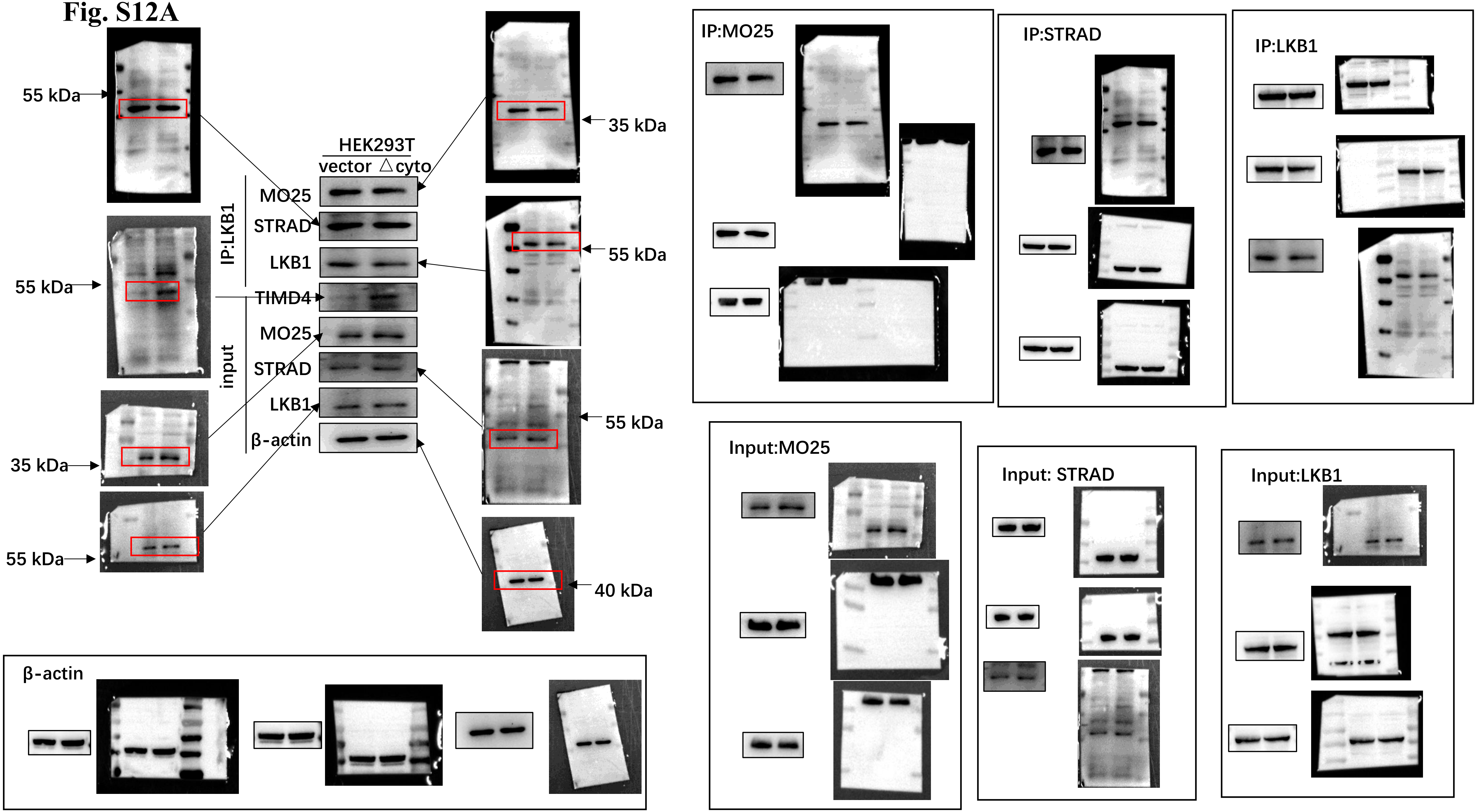
HEK293T
vector
△cyto
IP:LKB1
MO25
STRAD
LKB1
TIMD4
input
MO25
STRAD
LKB1
β-actin
55 kDa
55 kDa
35 kDa
55 kDa
35 kDa
55 kDa
55 kDa
40 kDa
β-actin
IP:MO25
IP:STRAD
IP:LKB1
Input:MO25
Input: STRAD
Input:LKB1

Fig. S12C

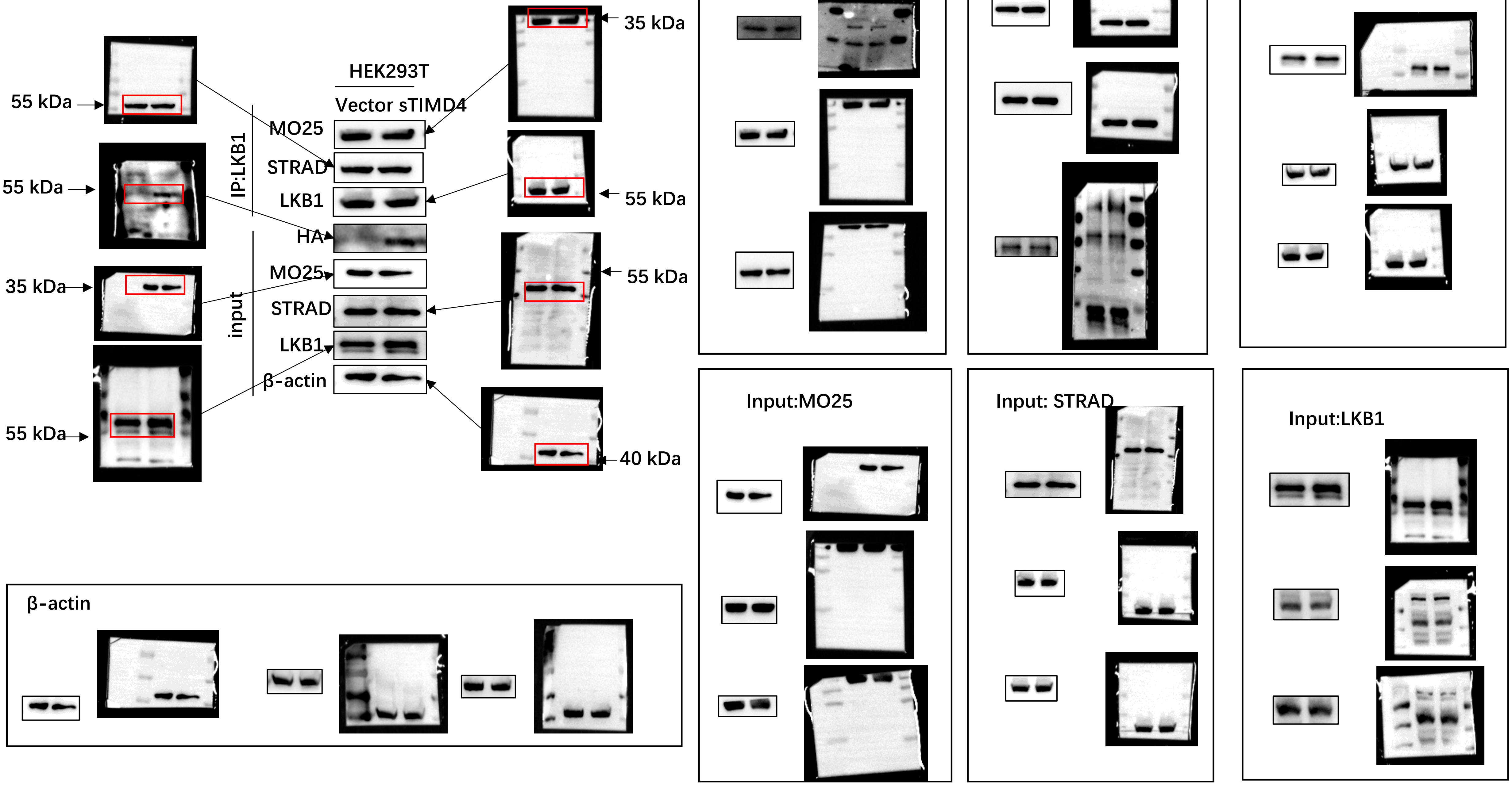

HEK293T
Vector sTIMD4
IP:LKB1
MO25
STRAD
LKB1
HA
input
MO25
STRAD
LKB1
β-actin
55 kDa
55 kDa
35 kDa
55 kDa
35 kDa
55 kDa
55 kDa
40 kDa
IP:MO25
IP:STRAD
IP:LKB1
Input:MO25
Input: STRAD
Input:LKB1
β-actin

Fig. S12E

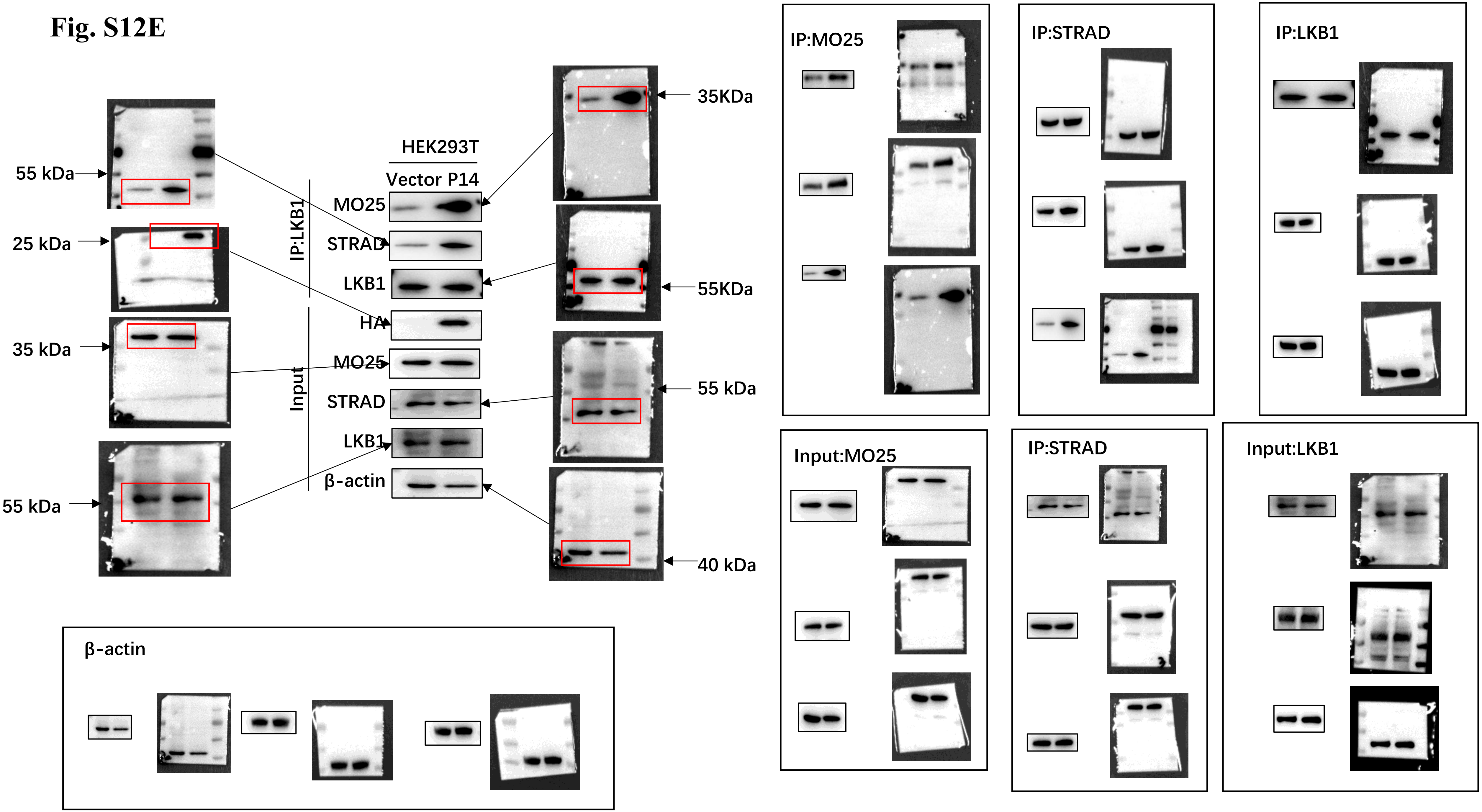

55 kDa
25 kDa
35 kDa
55 kDa
HEK293T
Vector P14
IP:LKB1
MO25
STRAD
LKB1
Input
HA
MO25
STRAD
LKB1
β-actin
35KDa
55KDa
55 kDa
40 kDa
IP:MO25
IP:STRAD
IP:LKB1
Input:MO25
IP:STRAD
Input:LKB1
β-actin

Fig. S13A

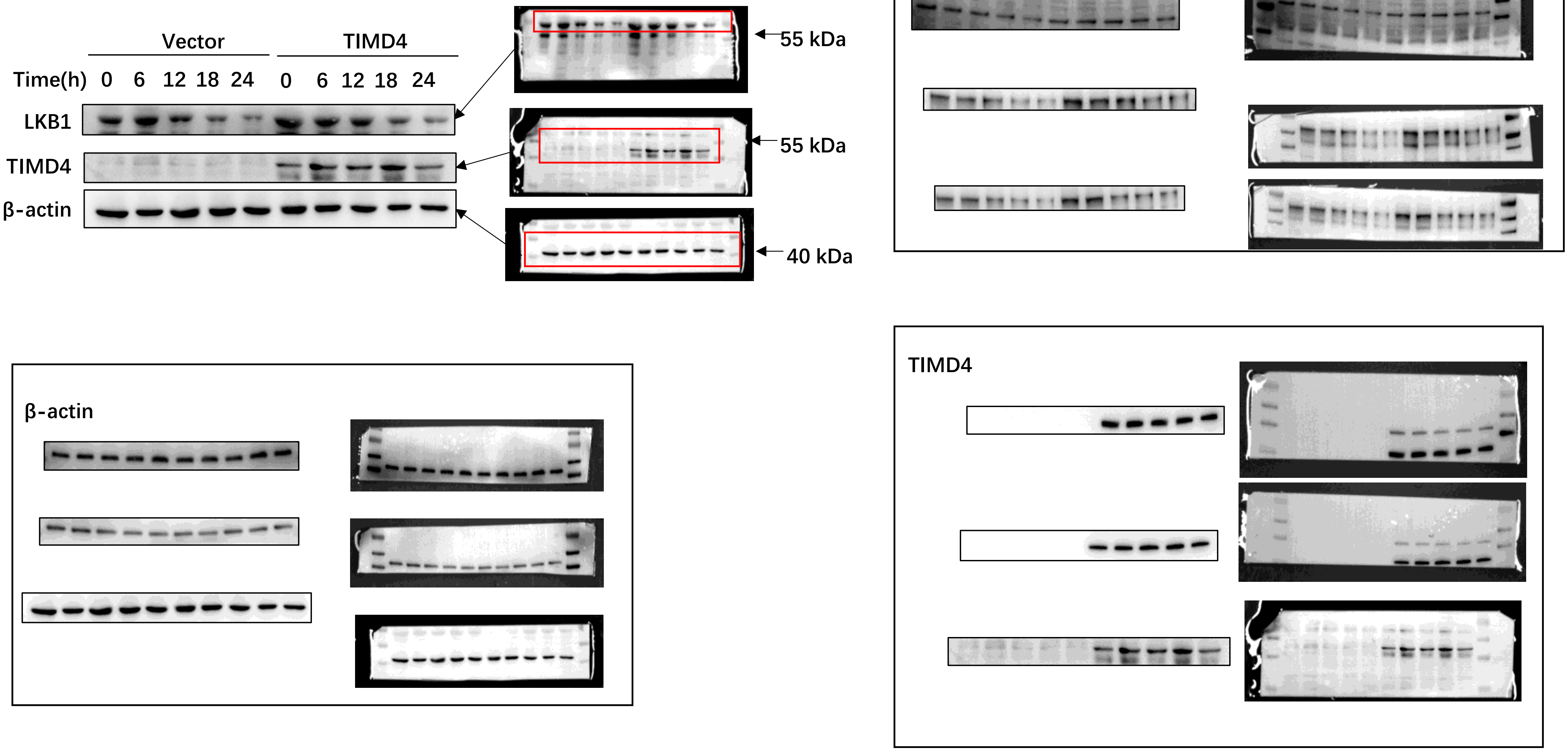

Vector
TIMD4
Time(h) 0 6 12 18 24 0 6 12 18 24
LKB1
TIMD4
β-actin
55 kDa
55 kDa
40 kDa
LKB1
β-actin
TIMD4

Fig. S13C

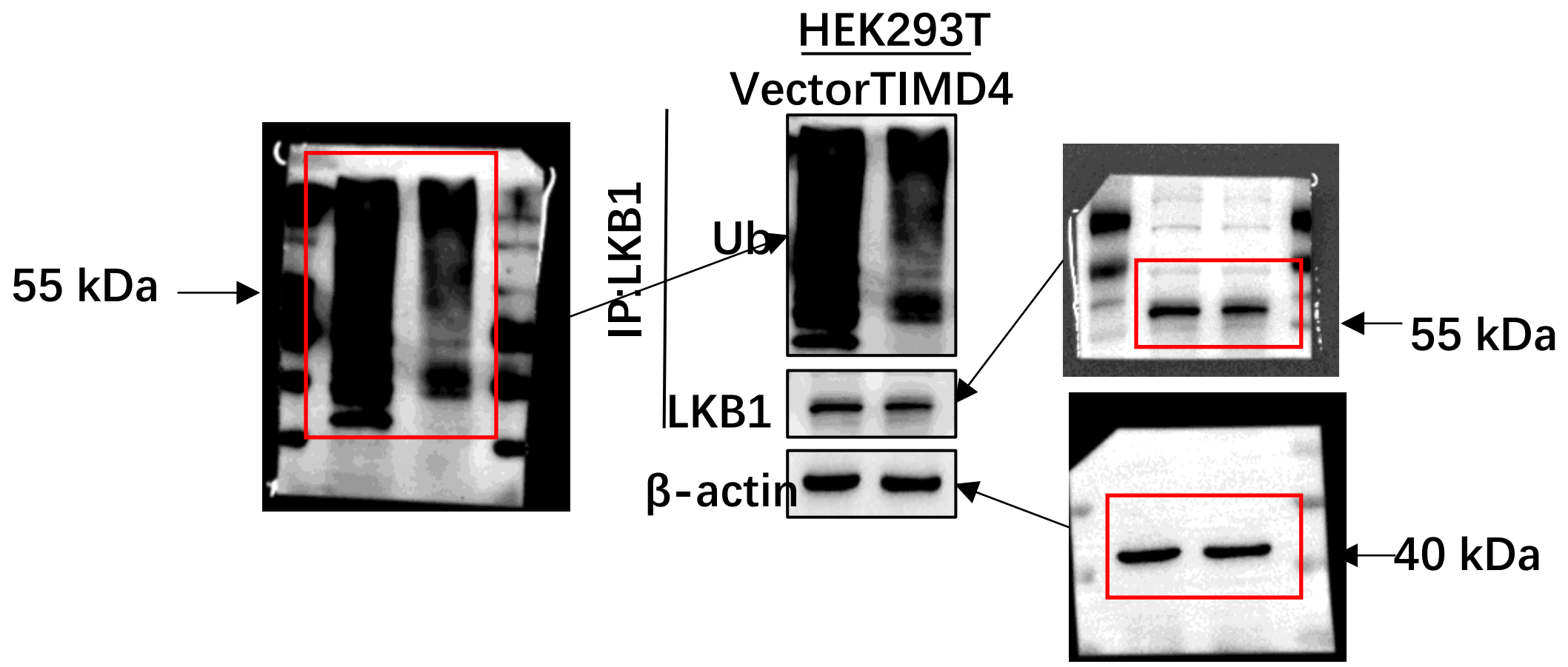

HEK293T
Vector TIMD4
IP:LKB1
Ub
LKB1
β-actin
55 kDa
55 kDa
40 kDa

Fig. S15E

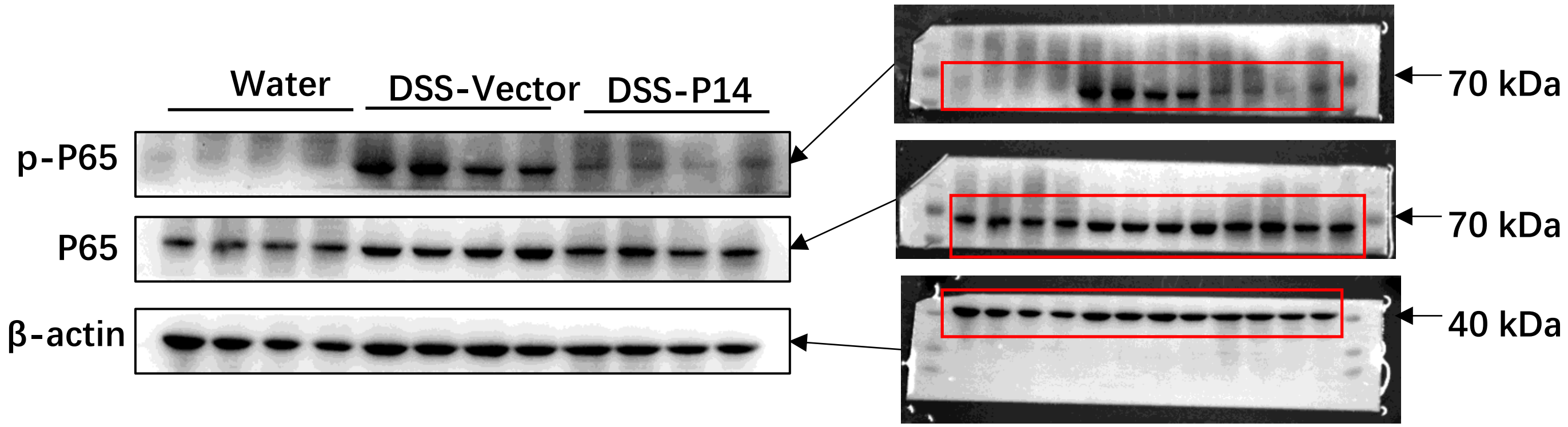

Supplement: Supplementary file 1 [file pbio.3004056.s027.pdf]
