# Supplementary material for: A macrophage-targeted TIMD4 Peptide-Nanoparticle attenuates colitis symptoms and monocyte recruitment in a mouse model
Source: PLoS Biol. 2026 Oct 21;24(10):e3004056. doi: 10.1371/journal.pbio.3004056 (PMC13686490; doi:10.1371/journal.pbio.3004056)
Supplement: Supplementary file 2 [file pbio.3004056.s031.docx]

We appreciate the insightful suggestions and comments raised by the editor and reviewers. We have given point-by-point responses to each comment and carefully revised the manuscript according to the reviewers’ comments.

**Reviewer #1:**

**This study fails to provide compelling evidence to support the definitive statement in the title or the mechanism proposed in the summary diagram.**

**Response:** We sincerely thank you for your careful review. To more fully support the statement in the title and the mechanism presented in the summary diagram, we have added further clarifications based on your comments, and provide detailed point-by-point responses below. We greatly appreciate your guidance, which will help us improve the manuscript greatly.

**1. The introduction and discussion consistently confuses mouse and human monocyte/macrophage biology. The species needs to clear at every point. For example, Ly6C is a marker for monocyte subsets only in mouse and F4/80 is not used as a marker in humans. The authors should employ the convention for naming genes and proteins. Timd4 for gene/TIMD4 for protein in rodents; TIMD4 for gene/mRNA/TIMD4 for protein in humans.**

**Response:** We thank the reviewer for pointing out the confusion of mouse and human species specificity in our use of monocyte/macrophage markers. We have re-examined the entire manuscript (including the Introduction, Results, and Discussion sections) and carefully checked and corrected all involved markers. Here we clarify the monocyte/macrophage subset markers used in our study and specify as belows:

(1) Mouse model (Fig. 2, Fig. 3): We used the DSS-induced mouse colitis model. To detect the co-localization of TIMD4 with macrophages, we performed co-staining of TIMD4 with CD68 (a macrophage marker shared by both mouse and human). For the analysis of macrophage proportions in the mouse colonic tissues, we used CD11b and F4/80 to label macrophages, and defined the CD11b⁺CX3CR1^int^Ly6Cʰⁱ cell subset as monocyte-derived macrophages. The use of these markers is consistent with mouse specificity.

(2) We have revised the nomenclature strictly according to the guidelines. Human samples (immunofluorescence analysis): We used only the human-specific marker CD68 to label monocytes/macrophages, and did not use Ly6C or F4/80. In the revised manuscript, *Timd4* is used for mouse gene/mRNA symbol, and *TIMD4* is used to represent human gene/mRNA symbol. TIMD4 is used for protein symbol.

All relevant modifications have been highlighted in red in the revised manuscript. We thank the reviewer again for their rigorous and constructive comments.

**2. They should also use accepted gene names/symbols (Stk11, Cap39, Strada/b)**

**Response:** We thank the reviewer for this rigorous and insightful comment. We fully agree that *Stk11*, *Cab39* and *Strada/b* are the approved gene symbols. However, given that the central focus of this study is the impact of TIMD4 on the structural stability of the LKB1‑STRAD‑MO25 protein complex, we have chosen to directly adopt the widely accepted protein-level designation “LKB1‑STRAD‑MO25” throughout the manuscript. Notably, this protein-level nomenclature has also been widely adopted in many studies describing the same ternary complex [1-3]. We once again thank the reviewer for the careful and rigorous evaluation.

**References:**

[1] Zeqiraj E, Filippi BM, Deak M, Alessi DR, van Aalten DM. Structure of the LKB1-STRAD-MO25 complex reveals an allosteric mechanism of kinase activation. Science. 2009;326(5960):1707-11. doi: 10.1126/science.1178377.PMID: 19892943.

[2] Alessi DR, Sakamoto K, Bayascas JR. LKB1-dependent signaling pathways. Annu Rev Biochem. 2006; 75:137-63. doi: 10.1146/annurev.biochem.75.103004.142702. PMID: 16756488.

[3] Fan G, Jin J, Wang J, Xu J, Ge W, Liu S, Tang L, Reziya W, Yang X, Li J, Ng LG, Di L, Zheng Y, Lan F, Chen S, Zhang Y, Wang X, Gao D, Liu X, Sun H, Liu Y, Li T, Cao Y, Tao Z, Liu W, Wang H. Cancer stem cells orchestrate immune evasion through extracellular vesicle-mediated non-canonical signaling pathways. Cancer Cell. 2026;44(6):1160-1178.e8. doi: 10.1016/j.ccell.2026.04.004. PMID: 42102811.

**3. TIMD4 has been proposed as a marker for long-lived resident intestinal macrophages in mice (see also PMID: 29789388) but** **immunolocalisation has been unconvincing and *Timd4* mRNA is very low in bulk isolated intestinal macrophages (****PMID: 33031383) or in total mRNA in mouse or human colon. The highest expression is in spleen, lymph node, bone marrow and liver (where TIMD4 is a Kupffer cell marker).**

**Response:** We thank the reviewer for the valuable comments. Regarding the reliability of TIMD4 as a marker for long-lived resident intestinal macrophages in mice, we provide the following response based on the literature.

Intestinal macrophages can be clearly divided into distinct subsets. Previous studies [4, 5] have classified adult intestinal macrophages into three subsets based on CD4 and TIMD4 expression: TIMD4⁻CD4⁺, TIMD4⁻CD4⁻, and TIMD4⁺CD4⁺. Among them, TIMD4⁺CD4⁺ macrophages are derived from fetal monocytes, represent the earliest macrophage population in the intestine, and persist into adulthood. The TIMD4⁻CD4⁺ and TIMD4⁻CD4⁻ populations originate from monocytes. The TIMD4⁺CD4⁺ subset thus serves as a marker for long-lived resident intestinal macrophages [6], and TIMD4 is a key marker that distinguishes fetal-derived, long-lived resident macrophages from short-lived macrophages derived from adult monocytes.

We agree that the highest expression is in spleen, lymph node, bone marrow and liver (where TIMD4 is a Kupffer cell marker). Regarding the reviewer’s concern that *Timd4* mRNA expression is very low in bulk-isolated intestinal macrophages or total colon mRNA, this is consistent with the relatively small proportion of the TIMD4⁺ subset within the total macrophage pool. In bulk sequencing, the presence of TIMD4⁻ macrophages dilute the signal, but this does not negate the reliable expression of TIMD4 in the specific subset. Indeed, when intestinal macrophages are enriched by TIMD4-based sorting, robust *Timd4* expression is readily detected [5]: “Surprisingly, despite the fact that TIMD4 was used as a marker to sort intestinal macrophages, the *Timd4* gene was not part of a significant co-expression cluster; it was highly expressed in the sorted intestinal macrophages but idiosyncratically in several other tissue macrophage populations.”

**References:**

[4] Shaw TN, Houston SA, Wemyss K, Bridgeman HM, Barbera TA, Zangerle-Murray T, Strangward P, Ridley AJL, Wang P, Tamoutounour S, Allen JE, Konkel JE, Grainger JR. Tissue-resident macrophages in the intestine are long lived and defined by Tim-4 and CD4 expression. J Exp Med. 2018 ;215(6):1507-1518. doi: 10.1084/jem.20180019. PMID: 29789388.

[5] Chiaranunt P, Burrows K, Ngai L, Mortha A. Isolation of mononuclear phagocytes from the mouse gut. Methods Enzymol. 2020; 632:67-90. doi: 10.1016/bs.mie.2019.10.004. PMID: 32000915.

[6] Jayaraman V, Prise IE, Kästele V, Tamburrano S, Wemyss K, Bridgeman HM, Daw RH, Strangward P, Sanderson A, Cruickshank SM, Konkel JE, Chew C, Yao C, Anderson CJ, Priller J, McColl B, Munro DAD, Martens L, Scott CL, Guilliams M, Adamson AD, Grainger JR, Shaw TN. CD163 and Tim-4 identify resident intestinal macrophages that are spatially regulated by TGF-β. J Exp Med. 2026;223(5):e20240801. doi: 10.1084/jem.20240801. PMID: 42012484.

**4. The rationale for this study is based upon the claim that TIMD4 is over-expressed in inflamed sites in ulcerative colitis. The IF images and quantitation in Figure 1A based upon the Santacruz antibody are unconvincing. The staining in the inflamed colon does not look cellular; colocalisation of a macrophage marker is needed and validation that the antibody binds TIMD4 exclusively on a Western blot.**

**Response:** Thank the reviewer for the valuable comments. We fully agree with the reviewer’s concerns and have made the following revisions accordingly:

(1) Regarding the immunofluorescence images in Fig. 1A and the quantification results based on the Santa Cruz antibody, we acknowledge that the original images were suboptimal and failed to clearly demonstrate the cellular localization of TIMD4. In the revised manuscript, we have replaced them with more representative images (revised Fig. 1A). The new images show that TIMD4-positive signals are predominantly distributed in a ring-like pattern around DAPI-labeled nuclei, consistent with a typical membrane/cytoplasmic localization pattern rather than non-specific staining.

(2) For the statistical analysis of TIMD4-positive cell proportions, we employed the TissueFAXS Spectra system combined with TissueFAXS Cytometry, a flow-cytometry-like multi-level quantification technique. To precisely exclude potential interference from “non-cellular TIMD4 staining” as the reviewer rightly pointed out, we set a spatial threshold during data analysis. Specifically, using the DAPI-labeled nuclear boundary as a reference, we defined an annular region extending from 0.32 μm inward to 0.63 μm outward from the nuclear boundary (i.e., a net effective radius of –0.32 μm to +0.63 μm). Only TIMD4 fluorescence signals localized within this annular region were counted as positive expression for a given cell, while signals beyond this range were excluded from quantification. By applying this threshold, we effectively eliminated non-cellular background interference, ensuring that the statistical results accurately reflect cell-associated TIMD4 expression.

(3) We appreciate the reviewer’s suggestion to examine the co-localization of TIMD4 with macrophage markers. In fact, we have performed this experiment, and the corresponding results are presented in revised Fig. 3L. Based on the overall logical structure of the manuscript, Fig. 1 primarily focuses on validating the importance of TIMD4 in the pathogenesis of colitis (including expression differences and clinical relevance). The co-localization of TIMD4 with macrophages and related functional experiments are systematically arranged in revised Fig. 2A and 3L. To avoid redundancy and maintain a smooth narrative flow, these co-localization data were not included in Fig. 1. We appreciate the reviewer’s suggestion. A description has been added to the main text: “Immunofluorescence staining revealed co-localization of CD68 and TIMD4 (revised Fig. 2A).”

(4) To validate the specificity of the antibody in Western blotting, we collected colonic tissue samples from 5 healthy controls and 5 ulcerative colitis (UC) patients and performed Western blotting analysis (revised Fig. 1C). The results showed that the antibody recognized a single specific band at the expected molecular weight (~55 kDa), with no detectable non-specific bands, confirming its specific binding to TIMD4 protein. Semi-quantitative analysis further revealed that TIMD4 expression levels in colonic tissues from UC patients were significantly higher than those in healthy controls (revised Fig. 1D, *p* < 0.01). These results provide protein-level evidence supporting the specificity of the antibody and also validate the upregulation of TIMD4 expression in UC.

We sincerely appreciate the insightful suggestions, which have been instrumental in strengthening the rigor and clarity of our revised manuscript. Thank you again for your time and expertise.

**Revised Fig. 1. *Timd4* deficiency aggravates DSS-induced colitis in mice.**

(A) Immunofluorescence staining images of colonic biopsy specimens from UC patients and healthy controls (normal: n = 4, UC: n = 7; scale bar: 20 μm). (B) Statistical analysis of TIMD4 protein expression in immunofluorescence image shown in A. (C, D) Western blotting image and statistical analysis of the relative expression level of TIMD4 expression in human colonic biopsy tissues (n = 5).

**5. The claim is supported by analysis of an older NCBI-GEO microarray data which shows relative rather than absolute expression. Notably, GSE47905 appears unrelated. The author should analyse data in PMID: 34450030 (bulk RNA-seq data GSE150961, single-cell RNA-seq data GSE150516).**

**Response:** We thank the reviewer for pointing out the error that “GSE47905 appears unrelated to this study.” We sincerely apologize for this mistake, which was indeed a clerical error on our part. The database accession number has been corrected to GSE47908, and the corresponding revision has been made in revised Fig. 1E of the revised manuscript.

Additionally, we acknowledge the reviewer’s second important concern: GDS3268, GDS35776 and GSE47908 are all RNA-seq datasets that present relative expression levels rather than absolute expression values. To address this limitation and further validate the expression changes of *TIMD4* at the single-cell level, we followed the reviewer’s suggestion and performed additional analyses on multiple publicly available single-cell RNA-seq datasets, including GSE150516, GSE144469, GSE162335, GSE231993, GSE242086, GSE253006 and GSE266616. For each dataset, we quantified the proportion of *TIMD4*-positive cells in patients with colitis versus healthy controls. A pooled analysis revealed that the proportion of *TIMD4*-positive cells in the colonic tissues of patients with colitis was significantly higher than that in healthy controls (revised Fig. 1F, *p* < 0.05).

Furthermore, we analyzed an additional independent dataset, GSE150961, which contains human colonic tissue samples collected before treatment and after 52 weeks of therapeutic follow-up. The results showed that *TIMD4* expression levels in the colonic tissues were significantly reduced after treatment compared to the untreated condition (revised Fig. 1G, *p* < 0.01). These findings further support the conclusion that TIMD4 is upregulated in the inflammatory microenvironment of colitis, suggesting that it may serve as a marker associated with disease activity or therapeutic response.

We sincerely thank the reviewer for the valuable and insightful comments, which have greatly contributed to strengthening the rigor and clarity of our revised manuscript.

**Revised Fig. 1. *Timd4* deficiency aggravates DSS-induced colitis in mice.**

(E) The data from GEO database of GDS3268/35776 and GSE47908 were collected to compare *TIMD4* mRNA expression level between colonic tissues from UC patients and normal colonic tissues (Normal colon: n = 69, UC inflamed colon: n = 63) (Normal colon: n = 15, UC inflamed colon: n = 45). (F) Validation of *TIMD4* expression changes at the single-cell level. Additional analyses of multiple publicly available single-cell RNA-seq datasets (GSE150516, GSE144469, GSE162335, GSE231993, GSE242086, GSE253006 and GSE266616) were performed (Healthy: n = 59, Colitis: n = 47). (G) Analysis of *TIMD4* expression in an RNA-seq dataset (GSE150961). Human colonic tissue samples were collected before treatment and after 52 weeks of therapeutic follow-up (Baseline: n = 331, 52wk FU: n = 87).

**6. As far as I can see TIMD4 was not identified as a disease-associated gene. The evidence that TIMD4 is increased in mouse DSS colitis Fig 1D, E, F, G is also unconvincing; the IHC quantification method is not described and the IHC result appears incompatible with the Western blot data.**

**Response:** We sincerely thank the reviewer for the valuable comments. Regarding your concerns that “TIMD4 has not been identified as a disease-associated gene” and the data on TIMD4 expression in the DSS colitis model, we provide the following point-by-point responses:

(1) Regarding the association between TIMD4 and disease

Although *TIMD4* has not been formally classified as a disease-associated gene, multiple studies have demonstrated a close relationship between TIMD4 and inflammatory bowel disease (IBD). At the genetic level, genome-wide association studies (GWAS) and Mendelian randomization analyses have identified *TIMD4* as a candidate gene for predicting IBD, and increased TIMD4 expression significantly elevates the risk of developing Crohn’s disease (CD) [7]. At the protein level, several studies have shown that TIMD4 expression is significantly upregulated in both UC patients and murine colitis models, and is positively correlated with disease activity and severity [8, 9]. Although these findings suggest that TIMD4 may participate in IBD progression, the underlying mechanisms remain unclear, which is precisely the scientific question that the present study aims to address.

(2) Regarding the IHC images and quantification methods

The IHC images and Western blotting data originally presented in Fig. 1D–G (revised Fig. 1H–K) were insufficiently representative, with suboptimal contrast of positive signals. In the revised manuscript, we have made the following improvements:

- Replacement of representative images: We have replaced them with typical images showing clearer staining and cleaner backgrounds (revised Fig. 1H, 1J). The IHC images clearly demonstrate a marked increase in TIMD4-positive cells in the colonic tissues of the DSS group, mainly distributed in areas of inflammatory cell infiltration. The Western blotting images also clearly show elevated TIMD4 expression in the DSS group.
- Supplementation of quantification methods: Systematic quantitative analysis was performed using ImageJ software. Specifically, the “Color Deconvolution” function (with the “H DAB” preset) was used to separate the DAB-positive signal from the hematoxylin counterstain, yielding a grayscale image representing the DAB signal. A unified positive threshold was then applied using the “Threshold” function to convert the positive signal into a binary image (white = positive, black = background). The “Analyze Particles” module was used to automatically count positive cells in each field, with appropriate size and circularity parameters. The same procedure was repeated for the channel representing cells (e.g., DAPI-stained nuclei) to obtain the total cell counts. The positivity rate was calculated as: TIMD4-positive cell rate = (number of positive cells / total number of cells) × 100 %. This quantification method has been detailed in the “Histopathological, immunohistochemical (IHC) and immunofluorescence (IF) analyses” section of the “Supplementary Materials and Methods”, and the statistical approach is described in the legend of revised Fig. 1.

(3) Regarding the apparent “incompatibility” between the IHC and Western blotting results, we have carefully re-examined the original data and confirmed that both methods show upregulated TIMD4 expression in DSS-induced colitis. The “incompatibility” may arise from the following reasons:

- Different detection principles: IHC detects the tissue localization and semi-quantitative level of TIMD4 protein, reflecting local expression intensity in specific cells; whereas Western blotting detects the total protein level in whole-layer colonic tissue lysates, which includes mixed signals from all cell types (including stromal cells, immune cells, epithelial cells, etc.).
- Differences in expression background: In colitis, the upregulation of TIMD4 may occur predominantly in infiltrating macrophages or tissue-resident macrophages. IHC can highlight the strong positivity in these cellular clusters, while Western blotting reflects the overall increase at the tissue level. Both methods are fully consistent in trend (both showing higher expression in the DSS group than in the control group), but the absolute magnitude of change or visual appearance cannot be directly compared across the two techniques.

All above revisions have been highlighted in red in the revised manuscript for the reviewer’s convenience. We once again thank the reviewer for the rigorous and insightful comments.

**References:**

[7] Zheng X, Zhang C, Zubair M, Pan J, Chen T, Yin J, Liu P, Luo P, Shen W, Chu Y. Decoding the Molecular Landscape of Inflammatory Bowel Disease: A Mendelian Randomization and Machine Learning Analysis. J Gastroenterol Hepatol. 2025;40(12):2867-2878. doi: 10.1111/jgh.70085. PMID: 41030199.

[8] Xue G, Hua L, Liu D, Zhong M, Chen Y, Zhou B, Xie Y, Li J. Tim-4 expressing monocytes as a novel indicator to assess disease activity and severity of ulcerative colitis. Life Sci. 2021; 269:119077. doi: 10.1016/j.lfs.2021.119077. PMID: 33465392.

[9] Liu Y, Wang Y, Wang X, Zhu L, Zhao C, Zhu J, Wang X. Analysis of Tim-4 expression in ulcerative colitis and its association with inflammation-related immune infiltration. Immunol Lett. 2026; 280:107173. doi: 10.1016/j.imlet.2026.107173. PMID: 41916431.

**Revised Fig. 1. *Timd4* deficiency aggravates DSS-induced colitis in mice.**

(H) Representative immunohistochemical staining images of colonic specimens obtained from DSS-induced colitis mice and controls, and the arrows indicate TIMD4^+^ cells. Scale bar: 50 μm. (I) Quantitative analysis of TIMD4^+^ cells in colonic tissue (n = 4). (J, K) Western blotting image and statistical analysis of the relative expression level of TIMD4 expression in colonic tissues from control mice or DSS-induced colitis mice (n = 5).

**7. In any case the model makes no sense. A role for TIMD4 as a sensor of apoptotic cells does not require that it be over-expressed.**

**Response:** We fully agree with the reviewer’s viewpoint that TIMD4, as an apoptotic cell sensor, does not depend on elevated expression levels for its function in recognizing apoptotic cells. However, it is worth noting that the functional role of TIMD4 extends well beyond that of an apoptotic cell sensor. A growing body of evidence indicates that TIMD4 also functions as a crucial molecule in regulating macrophage polarization, antiviral innate immunity, and cholesterol metabolism [10–16]. Moreover, in the tumor microenvironment, TIMD4⁺ macrophages also impair antitumor CD8⁺ T cell immunity [17].

In this context, we wish to clarify that the core objective of the present study is not to demonstrate that “TIMD4 upregulation is a prerequisite for its sensor function” but rather to investigate whether TIMD4 expression is altered in the pathological setting of colitis, and whether such alterations are associated with disease severity or the functional status of specific cell types (e.g., macrophages). Our data show that TIMD4 expression is upregulated in DSS-induced colitis. This observation is not contradictory to the basal functions of TIMD4; instead, it suggests that TIMD4 may be further induced in the inflammatory microenvironment to enhance apoptotic cell clearance or to participate in immune regulation. Indeed, increased TIMD4 expression has been widely reported in various inflammatory diseases: beyond the significant upregulation of TIMD4 in ulcerative colitis [18], elevated *Timd4* transcript levels have also been observed in CD301b⁺ macrophages in periodontitis [15]. These independent pieces of evidence from distinct inflammatory disease models collectively indicate that TIMD4 upregulation is a common phenomenon in inflammatory microenvironments, further supporting that the expression changes observed in our colitis model have a broad pathological basis. We sincerely thank the reviewer once again for the insightful and constructive feedback, which has significantly strengthened the rigor and clarity of our revised manuscript.

**References:**

[10] Wang Y, Wang Y, Ding L, Ren X, Wang B, Wang L, Zhao S, Yue X, Wu Z, Li C, Liang X, Ma C, Gao L. Tim-4 reprograms cholesterol metabolism to suppress antiviral innate immunity by disturbing the Insig1-SCAP interaction in macrophages. Cell Rep. 2022 ;41(9):111738. doi: 10.1016/j.celrep.2022.111738. PMID: 36450259.

[11] Liu W, Bai F, Wang H, Liang Y, Du X, Liu C, Cai D, Peng J, Zhong G, Liang X, Ma C, Gao L. Tim-4 Inhibits NLRP3 Inflammasome via the LKB1/AMPKα Pathway in Macrophages. J Immunol. 2019 ;203(4):990-1000. doi: 10.4049/jimmunol.1900117. PMID: 31263038.

[12] Xu LY, Qi JN, Liu X, Ma HX, Yuan W, Zhao PQ, Liang XH, Xu Y, Wang HX, Xu XY, Wang W, Ma CH, Gao LF. Tim-4 Inhibits NO Generation by Murine Macrophages. PLoS One. 2015; 10(4): e0124771. doi: 10.1371/journal.pone.0124771. PMID: 25905790

[13] Ding L, Liang Y, Wang Y, Tong Z, Liu W, Tan S, Zhang J, Wang Y, Wu Z, Liang X, Ma C, Gao L. T-cell immunoglobulin- and mucin-domain-containing molecule-4 maintains adipose tissue homeostasis by orchestrating M2 macrophage polarization via nuclear factor kappa B pathway. Immunology. 2023;168(1):49-62. doi: 10.1111/imm.13555. PMID: 35908188.

[14] Liu W, Xu L, Liang X, Liu X, Zhao Y, Ma C, Gao L. Tim-4 in Health and Disease: Friend or Foe? Front Immunol. 2020; 11:537. doi: 10.3389/fimmu.2020.00537. PMID: 32300343

[15] Wang Z, Zeng H, Wang C, Wang J, Zhang J, Qu S, Han Y, Yang L, Ni Y, Peng W, Liu H, Tang H, Zhao Q, Zhang Y. Tim4 deficiency reduces CD301b^+^ macrophage and aggravates periodontitis bone loss. Int J Oral Sci. 2024;16(1):20. doi: 10.1038/s41368-023-00270-z. PMID: 38418808.

[16] Wang X, Zhang Z, Zheng W, Zhang B, Chu C, Chen J, Liu C, Xu K, Yu Z, Guo Q, Zhao S, Chen X, Bai F, Wang B, Li X, Liu W. Macrophage Tim-4 protects against deep vein thrombosis by binding CK2β to suppress inflammatory responses. Front Immunol. 2025; 16:1634230. doi: 10.3389/fimmu.2025.1634230. PMID: 41064000.

[17] Chow A, Schad S, Green MD, Hellmann MD, Allaj V, Ceglia N, Zago G, Shah NS, Sharma SK, Mattar M, Chan J, Rizvi H, Zhong H, Liu C, Bykov Y, Zamarin D, Shi H, Budhu S, Wohlhieter C, Uddin F, Gupta A, Khodos I, Waninger JJ, Qin A, Markowitz GJ, Mittal V, Balachandran V, Durham JN, Le DT, Zou W, Shah SP, McPherson A, Panageas K, Lewis JS, Perry JSA, de Stanchina E, Sen T, Poirier JT, Wolchok JD, Rudin CM, Merghoub T. Tim-4+ cavity-resident macrophages impair anti-tumor CD8+ T cell immunity. Cancer Cell. 2021;39(7):973-988.e9. doi: 10.1016/j.ccell.2021.05.006. PMID: 34115989.

[18] Liu Y, Wang Y, Wang X, Zhu L, Zhao C, Zhu J, Wang X. Analysis of Tim-4 expression in ulcerative colitis and its association with inflammation-related immune infiltration. Immunol Lett. 2026; 280:107173. doi: 10.1016/j.imlet.2026.107173. PMID: 41916431.

**8. Methods state that "C57BL/6J wild-type (WT) mice were obtained from Vital River Laboratory Animal Technology. Timd4 knockout (KO) mice were provided by RIKEN BioResource Research Center (RBRC04895) [39]. Female mice (6-8 weeks old) were randomly divided into experimental and control groups". This is insufficient information. Ref [39] is not the original reference to the knockout. Precise genetic background and possible role of intestinal microbiome (PMID: 31004013) need to be considered. The comparison between WT and KO in a colitis model is really only valid if the WT controls are litter mates of the Timd4ko mice.**

**Response:** We sincerely thank the reviewer for the valuable comment. We agree that reference (Miyanishi et al., 2007, Nature, Identification of Tim4 as a phosphatidylserine receptor) only reported the receptor function of TIMD4, and is not the original literature describing the construction of this *Timd4* knockout (KO) mouse strain. After further literature verification, we confirmed that the construction and preliminary characterization of this knockout mouse (RBRC04895) was reported in detail by Yamazaki et. al. [19], and the genetic background of this strain is C57BL/6J. We have updated the citations in the Methods section of the revised manuscript, by adding this original construction literature, while retaining the original reference [20] as the primary citation for the functional characterization of TIMD4. In addition, we have explicitly indicated that this mouse strain is provided by the RIKEN BioResource Research Center, and have added the strain number RBRC04895 and its genetic background information in the Methods section. Thank you again for your rigorous comment.

Both the *Timd4* KO mice and C57BL/6J wild-type (WT) mice used in this study are on the C57BL/6J genetic background, and we have added a clear description of this genetic background information in the Methods section of the revised manuscript. It should be specifically noted that in the colitis experiments using *Timd4* KO and WT mice (revised Fig.1, Fig.2 and Fig.4), we used littermate KO mice and their littermate WT controls to ensure consistent genetic background between groups. For the subsequent peptide treatment experiment, the mice were obtained from Vital River Laboratory Animal Technology. We have explicitly clarified the source of mice used in the two sets of experiments in the Methods section of the revised manuscript to avoid confusion. Although we did not directly detect the microbiome in this study, the adoption of littermate controls in our experimental design has maximally controlled potential confounding caused by microbiome differences, and the relevant explanation has also been added to the Discussion section of the revised manuscript. Thank you again for the reviewer’s valuable comment.

**References:**

[19] Miyanishi M, Segawa K, Nagata S. Synergistic effect of Tim4 and MFG-E8 null mutations on the development of autoimmunity. Int Immunol. 2012; 24(9):551-9. doi: 10.1093/intimm/dxs064. PMID: 22723547.

[20] Miyanishi M, Tada K, Koike M, Uchiyama Y, Kitamura T, Nagata S. Identification of Tim4 as a phosphatidylserine receptor. Nature. 2007; 450(7168):435-9. doi: 10.1038/nature06307. PMID: 17960135.

**9. It is not clear why the DSS colitis model was examined only in juvenile (6-8 week) female mice. There is a substantial literature on male-female differences in the DSS colitis model. The apparent mild exacerbation in the Timd4ko mice could well be female-specific. Since the disease is more common and more severe in males this needs to be examined.**

**Response:** We sincerely appreciate the reviewer’s valuable comment. Regarding the selection of mouse age and sex, we initially used 6–8-week-old female mice for experiments. The main reason is that mice of this age range exhibit more pronounced weight loss in the DSS-induced acute colitis model, which provides a larger phenotypic window and facilitates more sensitive detection of phenotypic differences caused by gene knockout, which was consistent with the references [21]. We thank the reviewer for reminding us of the importance of considering sex factors in ulcerative colitis research. We agree the reviewer’s point that ulcerative colitis is indeed more common and severe in males, and verification of this factor is critical for this study.

The original rationale for selecting female mice is as follows: androgen signaling in male mice can regulate inflammatory responses, thereby introducing additional confounding variables into the experimental model. Multiple published studies [22, 23] have shown that female mice usually exhibit more pronounced or severe inflammatory phenotypes in classical models such as *Il10*^-/-^ spontaneous colitis and T cell-induced colitis. Therefore, to ensure model stability and focus on the core scientific question of this study, we chose female mice for the main experiments.

Nevertheless, we fully agree that whether the genetic phenotype exhibits sex specificity is key to evaluating its general applicability. To directly address and validate the reviewer’s concern, we specifically performed additional verification experiments: we used 6–8-week-old male C57BL/6J mice to establish a 3 % DSS-induced acute colitis model and examined the effect of *Timd4* deficiency on colitis progression (revised Fig. S4A). The results showed that, consistent with previous literature, the overall extent of weight loss in DSS-treated male mice was milder than that in female mice. However, under the same induction conditions, *Timd4* KO male mice exhibited a statistically significant difference in body weight compared with WT male mice starting from day 5 of modeling (revised Fig. S4B), accompanied by significantly elevated DAI scores (revised Fig. S4C), significantly shortened colon length (revised Fig. S4D, E) and significantly elevated pro-inflammatory cytokine levels (revised Fig. S4F). More importantly, histopathological analysis clearly demonstrated that *Timd4* deficiency significantly aggravated colonic tissue damage in male mice (revised Fig. S4G, K); PAS/AB staining and Mucin2 staining scores were also significantly reduced, as well as the expression of intestinal barrier proteins Claudin, Occludin and ZO-1 was significantly downregulated (revised Fig. S4H–J, L–N).

In summary, combining data from two independent experiments in female and male mice, we confirmed that the phenotype of *Timd4* deficiency exacerbating colitis is not sex-specific, and its pro-inflammatory function has been validated in mouse models of both sexes. We once again thank the reviewer for these insightful comments. These valuable suggestions prompted us to conduct more comprehensive verification and significantly strengthened the reliability of the conclusions of this study.

**References**

[21] Huaji Jiang, Yingchao Xie, Zhiqiang Hu et.al. VANGL2 alleviates inflammatory bowel disease by recruiting the ubiquitin ligase MARCH8 to limit NLRP3 inflammasome activation through OPTN-mediated selective autophagy. PLOS Biology. 2025;23(e3002961). oi.org/10.1371/journal.pbio.3002961

[22] Maite CB, Roy M, Emilie V. The Effect of Sex-Specific Differences on IL-10^-/-^ Mouse Colitis Phenotype and Microbiota. Int J Mol Sci. 2023;24(12):10364. doi: 10.3390/ijms241210364. PMID: 37373511.

[23] Maltz RM, Marte-Ortiz P, McClinchie MG, Hilt ME, Bailey MT. T Cell-Induced Colitis Is Exacerbated by Prolonged Stress: A Comparison in Male and Female Mice. Biomedicines. 2024; 12(1):214. doi: 10.3390/biomedicines12010214. PMID: 38255320.

**Revised Fig. S4. *Timd4* deficiency aggravates DSS‑induced acute colitis in male mice.**

(A) Experimental design: 6–8‑week‑old male C57BL/6J mice (WT and *Timd4* KO) were administered 3 % DSS in drinking water for 7 days to induce acute colitis, followed by 2 days of normal water. (B) Body weight changes were assessed daily. Statistics on the rate of weight loss on the day 5–9. (C) DAI scores were calculated based on body weight loss, stool consistency and rectal bleeding. (D) Representative images and (E) quantitative analysis of colon length. (F) ELISA measurement of pro-inflammatory cytokines (IL-6, IL-1β, TNF-α) in colonic homogenates. (G) Representative H&E staining images of colonic tissues. → Inflammation, ☆ Edema, the black dotted lines highlight the crypt. (H) Representative PAS staining and Alcian Blue (AB) staining images of colonic sections. (I, J) Representative immunofluorescence staining images of Mucin2, Claudin, Occludin and ZO‑1. Scale bar: 100 μm. (K) Histological scores based on H&E staining. (L) Histological scores based on PAS/AB staining. (M, N) Quantification of mean fluorescence intensity for Mucin2, Claudin, Occludin and ZO‑1. Data are presented as mean ± SEM (n = 4). Statistical significance was assessed using two‑tailed unpaired Student’s *t*‑test for comparisons between two groups. * *p* < 0.05, ** *p* < 0.01, *** *p* < 0.001.

**10. The authors attribute the impact of the Timd4ko on the DSS model to cell autonomous differences in recruited monocytes. The DSS model involves a massive granulocytosis and neutrophil infiltration. Given the major sites of expression of Timd4 in spleen, marrow and liver, indirect effects on granulocytes are equally plausible. What is the effect of the KO on blood cell homeostasis?**

**Response:** We sincerely thank the reviewer for this valuable comment. We have demonstrated in this study that TIMD4 plays a key role in regulating monocyte recruitment. Meanwhile, considering that neutrophils, T cells and other immune cells also play important roles in the pathogenesis of DSS-induced colitis, we further examined the expression levels of chemokines *Cxcl1*, *Cxcl2* *Cxcl10* and *Ccl20*, which are associated with neutrophil and T cell recruitment (revised Fig. S8). The results showed that *Timd4* deficiency not only upregulated the expression of monocyte chemokines but also enhanced the expression of chemokines related to neutrophils and T cells. These data suggest that the regulatory function of TIMD4 is not limited to inhibiting monocyte recruitment, but may also be broadly involved in suppressing the infiltration of multiple types of immune cells into the inflamed colonic tissues.

To directly investigate the effect of *Timd4* KO on blood cell homeostasis, we collected peripheral blood samples from WT and *Timd4* KO mice at the acute inflammatory phase of DSS-induced colitis (day 9 after DSS induction) and performed complete blood count analysis. The results are logically consistent with our above inference: although no statistically significant difference was observed between the WT and *Timd4* KO groups, we detected a consistent downward trend in the absolute counts of leukocytes, lymphocytes, monocytes and neutrophils, as well as the relative proportions of monocytes and neutrophils in the peripheral blood of *Timd4* KO mice (Fig. Ⅰ A-G). These results suggest that enhanced local chemokine signaling in the colon after *Timd4* deficiency promotes the recruitment of more immune cells from the peripheral blood to the inflamed tissues, which may lead to a relative reduction in the counts of these cells in peripheral blood. This finding is consistent with our previous observation that *Timd4* KO mice develop more severe colonic inflammation.

In addition, in the peripheral blood of *Timd4* KO mice, RBC, HGB and HCT were elevated, while RDW was decreased (Fig. Ⅰ H–J), indicating peripheral erythrocytosis. This phenotype is closely related to the role of TIMD4 in mediating the clearance of senescent red blood cells [24–26]. After *Timd4* deletion, senescent red blood cells cannot be effectively cleared, which may inhibit the generation of new red blood cells through negative feedback regulation, resulting in a relatively homogeneous population of red blood cells in the circulation with reduced size variability, thereby decreasing RDW.

**References:**

[24] Willekens FL, Werre JM, Kruijt JK, Roerdinkholder-Stoelwinder B, Groenen-Döpp YA, van den Bos AG, Bosman GJ, van Berkel TJ. Liver Kupffer cells rapidly remove red blood cell-derived vesicles from the circulation by scavenger receptors. Blood. 2005;105(5):2141-5. doi: 10.1182/blood-2004-04-1578. PMID: 15550489.

[25] Terpstra V, van Berkel TJ. Scavenger receptors on liver Kupffer cells mediate the in vivo uptake of oxidatively damaged red blood cells in mice. Blood. 2000;95(6):2157-63. PMID: 10706889.

[26] Neri S, Swinkels DW, Matlung HL, van Bruggen R. Novel concepts in red blood cell clearance. Curr Opin Hematol. 2021;28(6):438-444. doi: 10.1097/MOH.0000000000000679. PMID: 34494977.

**Fig.** **Ⅰ. Impact of *Timd4* deficiency on peripheral blood cell counts during DSS-induced colitis.**

Peripheral blood samples were collected from WT and *Timd4* KO mice at the acute inflammatory phase (day 9 after DSS induction) and subjected to complete blood count analysis. (A) Absolute counts of leukocytes. (B, C) Absolute counts and relative percentage of lymphocytes. (D, E) Absolute counts and relative percentage of monocytes. (F, G) Absolute counts and relative percentage of granulocyte. (H) Absolute counts of red blood cells (RBC). (I) Hemoglobin concentration (HGB, g/L); (J) Hematocrit (HCT, %); (K) Mean corpuscular volume (MCV, fL); (L) Mean corpuscular hemoglobin (MCH, pg); (M) Mean corpuscular hemoglobin concentration (MCHC, g/L); (N) Red blood cell distribution width (RDW, %). Data are presented as mean ± SEM (n = 3). The unpaired student’s *t*-test was employed to assess differences between two and multiple groups. Statistical significance was reported as * *p* < 0.05, ** *p* < 0.01, *** *p* < 0.001 and ns, no significance.

**11. There is no detectable expression of Timd4 mRNA in mouse bone marrow-derived macrophages in published data (see e.g. data on BioGPS.org, also PMID: 34134993). This being the case, it is not clear why BMDM from Timd4ko mice would be different from WT. The authors appear to demonstrate TIMD4 protein in BMDM and its loss in the KO in Figure S8. There is insufficient detail provided on how the BMDM were generated. What cell concentration, substratum, where they harvested and replated? If so, how? TIMD4 is expressed by resident macrophage in bone marrow. I wonder if the authors have maintained these cells in their cultures. In any case, they need to show surface TIMD4 on their BMDM by FACS.**

**Response:** We sincerely thank the reviewer for this valuable comment. We apologize for the confusion caused by the lack of detailed protocols for bone marrow-derived macrophages (BMDM) generation, including seeding density and other experimental details, in our original manuscript. We now provide the full detailed protocol for BMDM preparation below.

BMDMs were isolated from the tibia and femur of WT and *Timd4* KO mice. Briefly, mice were euthanized by CO₂ inhalation and immersed in 75 % ethanol for 3 minutes for disinfection. After placing and securing the mice on a clean bench, the skin and muscle tissues attached to the tibia and fibula were carefully removed using autoclaved scissors and forceps, keeping the bone ends intact. The intact bones were then soaked in 75 % ethanol for another 3 minutes, followed by immersion in PBS for 3 minutes to remove residual ethanol. After cutting the bone ends to expose the marrow cavity, PBS was flushed through using a 5 mL syringe to harvest bone marrow cells. The collected cell suspension was centrifuged at 1200 rpm for 6 minutes. Following removal of the supernatant, red blood cells were lysed with RBC lysis buffer on ice for 8 minutes. Cell pellets were then collected by centrifugation. The cells were resuspended in DMEM complete medium supplemented with 10 % FBS, 1 % penicillin-streptomycin, and 100 ng/mL M-CSF. After counting, the cells were seeded at a density of 3 × 10⁶ cells per 6 cm dish. After 3 days of culture at 37 °C with 5 % CO₂, fresh DMEM complete medium containing 100 ng/mL M-CSF was added, and the cells were cultured for another 2 days to obtain mature BMDMs.

As the reviewer correctly pointed out, the expression level of TIMD4 in BMDMs is indeed lower than that in PEMs. However, previous studies have confirmed that TIMD4 is expressed in BMDMs [27, 28]. As suggested, we detected TIMD4 expression by flow cytometry and RT-qPCR. Our results clearly showed that TIMD4 expression levels in WT BMDMs were significantly higher than those in *Timd4* KO BMDMs (Fig. Ⅱ). Nevertheless, we fully understand the reviewer’s concern and have accordingly performed additional experiments in PEMs. Specifically, we examined the expression of monocyte chemokines in PEMs (revised Fig. S8A) and the phosphorylation levels of LKB1, AMPK, and NF-κB in PEMs (revised Fig. S10). The results obtained from PEMs were consistent with those from BMDMs. Therefore, the use of BMDMs in our experiments is justified.

We thank the reviewer again for the constructive comments, which have helped us improve the rigor and clarity of our work.

**References:**

[27] Zeng L, Wang Y, Huang Y, Yang W, Zhou P, Wan Y, Tao K, Li R. IRG1/itaconate enhances efferocytosis by activating Nrf2-TIM4 signaling pathway to alleviate con A induced autoimmune liver injury. Cell Commun Signal. 2025; 23(1):63. doi: 10.1186/s12964-025-02075-5. PMID: 39910615.

[28] Han Y, Hu J, Pan J, Song X, Zhou Y, Zhang J, Yang Y, Shi X, Yang J, Sun M. LPS exposure alleviates multiple tissues damage by facilitating macrophage efferocytosis. Int Immunopharmacol. 2024; 135:112283. doi: 10.1016/j.intimp.2024.112283. PMID: 38772299.

**Fig. Ⅱ. Validation of TIMD4 expression in WT and *Timd4* KO BMDMs.**

TIMD4 expression levels were assessed in WT and *Timd4* KO BMDMs using flow cytometry and Reverse transcription quantitative real-time PCR (RT-qPCR). (A) Representative flow cytometry histograms showing TIMD4 expression profiles in WT (blue) and *Timd4* KO (red) BMDMs. (B) Statistical analysis of flow cytometry data (left panel: percentage of TIMD4-positive cells; right panel: mean fluorescence intensity, MFI). (C) RT-qPCR analysis showing significantly higher relative *Timd4* mRNA expression levels in WT BMDMs than in *Timd4* KO BMDMs. Data are presented as mean ± SEM (n = 3). Unpaired student’s *t*-test statistical was employed to assess differences between two groups. Statistical significance was reported as *** *p* < 0.001.

**Revised Fig. S8. *Timd4* deficiency increases the expression of chemokines.**

(A) PEMs were obtained from the peritoneal cavity of mice. RNA extracted after macrophages stimulated for 24 h with LPS (100 ng/mL). Detection of chemokine expression by RT-qPCR. Data are presented as mean ± SEM (n = 3). Two-way ANOVA was employed to assess differences between two and multiple groups. Statistical significance was reported as * *p* < 0.05, ** *p* < 0.01, *** *p* < 0.001 and ns, no significance.

**Revised Fig. S10. TIMD4 inhibits NF-κB activation through the LKB1-AMPK axis.**

(A) GSEA analysis of RNA‑seq data from resident macrophages of WT and *Timd4* KO mice, showing enrichment of the NF‑κB signaling pathway in *Timd*4 KO cells. (B) Representative immunoblots of phosphorylated P65 (p‑P65) and total P65 in colonic tissues from WT and *Timd4* KO mice. (C) Quantification of p‑P65/total P65 ratio. (D) Representative immunoblots of p‑LKB1, p‑AMPK and p‑P65 in WT and *Timd4* KO PEMs stimulated with increasing concentrations of LPS (0, 10, 50, 100 ng/mL) for 30 min. (E) Representative immunoblots of p‑LKB1, p‑AMPK and p‑P65 in WT and *Timd4* KO PEMs treated with or without the P65 inhibitor QNZ (10 μM, BML-275, 1 h) prior to LPS stimulation (100 ng/mL, 30 min). (F) Representative immunoblots of p‑LKB1, p‑AMPK and p‑P65 in WT and *Timd4* KO PEMs treated with or without the AMPK inhibitor Compound C (BML-275, 10 μM, 1 h) prior to LPS stimulation (100 ng/mL, 30 min). (G–I) Quantification of p‑LKB1, p‑AMPK and p‑P65 levels corresponding to the LPS dose‑response experiment shown in (D). (J–L) Quantification of p‑LKB1, p‑AMPK and p‑P65 levels corresponding to the QNZ treatment experiment shown in (E). (M–O) Quantification of p‑LKB1, p‑AMPK and p‑P65 levels corresponding to the Compound C treatment experiment shown in (F). (P) Representative immunoblots of total LKB1, p‑LKB1, p‑AMPK and p‑P65 in WT and *Timd4* KO PEMs transfected with control siRNA or LKB1 siRNA, followed by LPS stimulation (100 ng/mL, 30 min). Q–S) Quantification of p‑LKB1, p‑AMPK and p‑P65 levels corresponding to the LKB1 knockdown experiment shown in (P). Data are presented as mean ± SEM (n = 3). The unpaired student’s *t*-test and two-way ANOVA were employed to assess differences between two and multiple groups. Statistical significance was reported as * *p* < 0.05, ** *p* < 0.01, *** *p* < 0.001 and ns, no significance.

**12. There is a massive jump of logic with minimal data support to invoke the STK11/CAP39/STRAD pathway in TIMD4 signalling. It seems to rely on the data in Fig S8 apparently showing the effect of AMPKinase inhibitor and LKB1(STK11) knockdown. There is no indication provided of the reproducibility of the Western blots in this Figure. It is well known that LPS promotes dephosphorylation of AMPKinase and inhibitors increase the LPS response. It is not clear why they show only the LPS-stimulated state in the Western blots. There is no dose response, no control for the knockdown. The fact that the effects of the inhibitor of AMPkinase are not additive with the apparent effect of the Timd4 KO does not necessarily imply that Timd4 acts via AMPKinase. Subsequent analysis in Figure 5 relies almost entirely on over-expression and co-IP localisation in HEK293 cells. There is no compelling evidence that this pathway is engaged in macrophages.**

**Response:** We sincerely thank the reviewer for the valuable comment. Regarding the concern about insufficient evidence for the involvement of the LKB1-AMPK pathway in TIMD4 signaling, we have conducted a series of validation experiments. Our detailed response is as follows:

In this study, we initially found through RNA sequencing of macrophages and immunoblotting of colonic tissues that TIMD4 inhibits NF-κB activation (revised Fig. S10A-C). Building on our previous findings that TIMD4 promotes AMPK activation via LKB1 [29], together with published evidence demonstrating that AMPK signaling can suppress NF-κB-driven inflammatory responses through its downstream effectors such as SIRT1, PGC-1α, p53 and forkhead box O (FoxO) factors [30, 31], we proposed the hypothesis that “TIMD4 regulates NF-κB activation through the LKB1-AMPK axis.” To validate this hypothesis, we performed the following additional experiments.

(1) LPS dose-response experiments: PEMs from WT and *Timd4* KO mice were stimulated with increasing concentrations of LPS (0, 10, 50, 100 ng/mL) for 30 minutes. Cell lysates were collected and analyzed for p-LKB1, p-AMPK and p-P65 levels. The results demonstrated that as LPS concentration increased, p-LKB1 and p-AMPK levels gradually and significantly decreased, while p-P65 levels gradually and significantly increased. Importantly, at all LPS concentrations, WT PEMs exhibited significantly higher levels of p-LKB1 and p-AMPK and significantly lower levels of p-P65, compared to *Timd4* KO PEMs, establishing a clear dose-response relationship (revised Fig. S10 D, G-I).

(2) Hierarchical pathway validation experiments: To clarify the regulatory relationships within this pathway, we performed treatments with the P65 inhibitor QNZ, the AMPK inhibitor Compound C and siRNA-mediated knockdown of LKB1. The results are summarized as follows:

- P65 inhibitor QNZ treatment: Inhibition of P65 by QNZ abolished the difference in P65 phosphorylation levels between WT and *Timd4* KO PEMs, while LKB1 and AMPK phosphorylation levels remained unaffected (revised Fig. S10 E, J–L). Consistently, the difference in chemokine expression between WT and *Timd4* KO groups was also completely eliminated, mirroring the changes in p-P65 levels. These results indicate that TIMD4’s regulation of chemokine expression is dependent on P65 activation status (revised Fig. S11 A).
- AMPK inhibitor Compound C treatment: Inhibition of AMPK by Compound C eliminated the difference in AMPK phosphorylation levels between WT and *Timd4* KO PEMs. Concomitantly, P65 phosphorylation levels increased and the difference between the two groups disappeared (revised Fig. S10 F, M-O). At the chemokine expression level, the differences between WT and *Timd4* KO groups were also abolished. These findings further support the conclusion that TIMD4 regulates NF-κB downstream chemokine expression via AMPK signaling (revised Fig. S11 B).
- LKB1 siRNA knockdown: Knockdown of LKB1 by siRNA reduced total LKB1 protein levels and eliminated the difference in p-LKB1 phosphorylation between WT and *Timd4* KO groups. Meanwhile, AMPK phosphorylation levels decreased and P65 phosphorylation levels increased (revised Fig. S10 P–S). Chemokine expression analysis revealed that the differences between WT and *Timd4* KO groups were also eliminated, consistent with the changes observed in p-LKB1, p-AMPK and p-P65 immunoblotting results (revised Fig. S11 C).

Taken together, these results validate the hierarchical role of the LKB1-AMPK-P65 signaling axis in TIMD4-mediated regulation of monocyte chemokine expression. All supplementary experiments have now been completed, and the corresponding results will be updated in the main text and supplementary materials. Regarding reproducibility, all newly performed Western blotting experiments were independently repeated three times, and the results were highly reproducible. We have clearly indicated the experimental replication information in the revised figure legends.

(3) Additional experiments addressing revised Fig. 5 concerns: To address the reviewer’s concern that the analysis in revised Fig. 5 relied almost entirely on HEK293 cell overexpression and IP localization, lacking direct evidence of pathway activation in macrophages, we conducted additional experiments. We overexpressed various *Timd4* truncation mutants in the mouse monocyte cell line RAW264.7 and performed IP assays (Revised Fig. 5Q, R). The results demonstrated that the intracellular domain (ICD) of TIMD4 plays a critical role in promoting the formation of the LKB1-MO25-STRAD complex in RAW264.7 cells. These findings further confirm that this pathway is conserved and functional in macrophages.

We once again sincerely thank the reviewer for the valuable time and professional insights. Your constructive comments have significantly improved the rigor and persuasiveness of this work.

**References:**

[29] Liu W, Bai F, Wang H, Liang Y, Du X, Liu C, Cai D, Peng J, Zhong G, Liang X, Ma C, Gao L. Tim-4 Inhibits NLRP3 Inflammasome via the LKB1/AMPKα Pathway in Macrophages. J Immunol. 2019; 203(4):990-1000. doi: 10.4049/jimmunol.1900117. PMID: 31263038.

[30] Xu Y, Bai L, Yang X, Huang J, Wang J, Wu X, Shi J. Recent advances in anti-inflammation via AMPK activation. Heliyon. 2024; 10(13):e33670. doi: 10.1016/j.heliyon.2024.e33670. PMID: 39040381.

[31] Salminen A, Hyttinen JM, Kaarniranta K. AMP-activated protein kinase inhibits NF-κB signaling and inflammation: impact on healthspan and lifespan. J Mol Med (Berl). 2011;89(7):667-76. doi: 10.1007/s00109-011-0748-0. PMID: 21431325.

 **Revised Fig. S10. TIMD4 inhibits NF-κB activation through the LKB1-AMPK axis.**

(A) GSEA analysis of RNA‑seq data from resident macrophages of WT and *Timd4* KO mice, showing enrichment of the NF‑κB signaling pathway in *Timd4* KO cells. (B) Representative immunoblots of phosphorylated P65 (p‑P65) and total P65 in colonic tissues from WT and *Timd4* KO mice. (C) Quantification of p‑P65/total P65 ratio. (D) Representative immunoblots of p‑LKB1, p‑AMPK and p‑P65 in WT and *Timd4* KO PEMs stimulated with increasing concentrations of LPS (0, 10, 50, 100 ng/mL) for 30 min. (E) Representative immunoblots of p‑LKB1, p‑AMPK and p‑P65 in WT and *Timd4* KO PEMs treated with or without the P65 inhibitor QNZ (10 μM, BML-275, 1 h) prior to LPS stimulation (100 ng/mL, 30 min). (F) Representative immunoblots of p‑LKB1, p‑AMPK and p‑P65 in WT and *Timd4* KO PEMs treated with or without the AMPK inhibitor Compound C (BML-275, 10 μM, 1 h) prior to LPS stimulation (100 ng/mL, 30 min). (G–I) Quantification of p‑LKB1, p‑AMPK and p‑P65 levels corresponding to the LPS dose‑response experiment shown in (D). (J–L) Quantification of p‑LKB1, p‑AMPK and p‑P65 levels corresponding to the QNZ treatment experiment shown in (E). (M–O) Quantification of p‑LKB1, p‑AMPK and p‑P65 levels corresponding to the Compound C treatment shown in (F). (P) Representative immunoblots of total LKB1, p‑LKB1, p‑AMPK and p‑P65 in WT and *Timd4* KO PEMs transfected with control siRNA or LKB1 siRNA, followed by LPS stimulation (100 ng/mL, 30 min). (Q–S) Quantification of p‑LKB1, p‑AMPK and p‑P65 levels corresponding to the LKB1 knockdown experiment shown in (P). Data are presented as mean ± SEM (n = 3). The unpaired student’s *t*-test and two-way ANOVA were employed to assess differences between two and multiple groups. Statistical significance was reported as * *p* < 0.05, ** *p* < 0.01, *** *p* < 0.001 and ns, no significance.

**Revised Fig. S11. TIMD4 regulates chemokine expression through the LKB1-AMPK-NF-κB axis.**

BMDMs from WT and *Timd4* KO mice were subjected to the indicated treatments, and mRNA expression levels of the chemokines *Ccl2*, *Ccl3*, *Ccl5*, *Ccl7* and *Ccl8* were quantified by RT-qPCR. (A) Effect of NF‑κB inhibition. Cells were pretreated with the NF‑κB inhibitor QNZ (EVP4593, 10 μM) for 1 h, followed by LPS stimulation (100 ng/mL) for 24 h. (B) Effect of AMPK inhibition. Cells were pretreated with the AMPK inhibitor Compound C (BML‑275, 10 μM) for 1 h, followed by LPS stimulation (100 ng/mL) for 24 h. (C) Effect of LKB1 knockdown. Cells were transfected with LKB1‑specific siRNA or control siRNA for 24 h, then stimulated with LPS (100 ng/mL) for 24 h. Data are presented as mean ± SEM (n = 3). Two-way ANOVA was employed to assess differences between multiple groups. Statistical significance was reported as * *p* < 0.05, ** *p* < 0.01, *** *p* < 0.001 and ns, no significance.

**Revised Fig. 5. TIMD4 promotes LKB1 phosphorylation by stabilizing the LKB1-MO25-STRAD complex.**

(Q, R) IP assays were performed using anti‑LKB1 antibody in RAW264.7 cells transfected with empty vector, *TIMD4*, Δcyto, s*TIMD4*, or ICD. Representative Western blotting images and quantification of MO25 and STRAD protein levels in the IP relative to LKB1 are shown. All data are presented as mean ± SEM (n = 3). One -way ANOVA was employed to assess differences between multiple groups. Statistical significance was reported as * *p* < 0.05, ** *p* < 0.01.

**13. A second jump in logic proposes "synergistic" effects of a TIMD4 peptide and Vedolizumab. The latter is a humanised antibody against a4/b7 integrin. The source of the antibody is not provided, there is no indication of a rationale for the dose, no control human Ig, and no evidence that the antibody reacts with mouse (which it does not PMID: 33847343.**

**Response:** We sincerely appreciate your valuable comments. We fully understand your concerns regarding antibody selection and experimental design rigor, and we sincerely apologize for any confusion caused by the lack of clarity in our original manuscript. We would like to address each of your key points as follows:

(1) Regarding the antibody name and species reactivity: The term “Vedolizumab” used in the original manuscript was an inaccurate description and has now been corrected. The antibody actually used in this study was the “Purified anti-mouse LPAM-1 (Integrin α4β7) Antibody” (BioLegend, clone DATK32). This antibody specifically recognizes mouse α4β7 integrin and is effective in our experimental system. The correction has been made in the revised manuscript. The antibody information has been included in the figure legend for revised Fig. 7 in the revised manuscript.

(2) Regarding the dosing rationale: The antibody dosing regimen (50 μg per mouse, three times per week) was based on a standardized protocol established for functional studies of this antibody [32]. To more rigorously evaluate the therapeutic efficacy of the combination of T4_(365-378)_-nanoparticles with anti-α4β7 antibody, we intentionally reduced the antibody dose to 50 % of the standard dose. This approach was designed to determine whether the combination treatment could still produce a significant synergistic effect under suboptimal dosing conditions, thereby providing a more stringent test of the combined therapeutic strategy.

(3) Regarding the control setting: We admit that the original manuscript lacked a description of the isotype control antibody. We sincerely apologize for this omission. Upon receiving your comment, we performed the additional control experiment. The supplementary data confirmed that the isotype control group did not produce effects comparable to those observed in the anti-α4β7 antibody group, thus verifying that the observed phenotypic changes resulted from specific blockade of α4β7 integrin. These data and the corresponding description have been incorporated into the revised manuscript.

We thank you for your thorough and professional review. Your comments are invaluable for improving the rigor and clarity of our study.

**References:**

[32] Sommer K, Heidbreder K, Kreiss L, Dedden M, Paap EM, Wiendl M, Becker E, Atreya R, Müller TM, Atreya I, Waldner M, Schürmann S, Friedrich O, Neurath MF, Zundler S. Anti-β7 integrin treatment impedes the recruitment on non-classical monocytes to the gut and delays macrophage-mediated intestinal wound healing. Clin Transl Med. 2023; 13(4):e1233. doi: 10.1002/ctm2.1233. PMID: 37029786.

**14. Descriptions of methods and the figure legends our uniformly entirely inadequate. To cite just three examples: Figure 3 includes an analysis of RNA-seq data focussed on chemokine gene expression. The full extent of the legend is: B, C) Resident macrophages from WT and *Timd4* KO mice were collected and subjected to RNA sequencing. B) Heat map analysis of chemokines. C) GSEA analysis of RNA-seq data. The methods state that: Mouse tissue-resident macrophages were stimulated with LPS (100 ng/mL) for 3 h. RNA-seq was carried out by the Beijing Genomics Institute. Precisely what cells, how purified, how sequenced, how quantified, full dataset…….**

**Response:** We appreciate the reviewer’s comment regarding the inadequacy of the method descriptions and figure legends. We fully acknowledge this concern, and in response to the specific criticism regarding Fig. 3, we have comprehensively revised and expanded in “Supplementary Materials”.

(1) Cell source and purification:

Mice were euthanized by CO₂ asphyxiation. Fresh tissues were collected from mice and digested with collagenase. The digested tissue suspension was filtered through a 70 μm cell strainer to obtain a single‑cell suspension. The cell pellet was then resuspended in MACS separation buffer (PBS containing 0.5 % BSA and 2 mM EDTA) for cell counting. The cells were thoroughly mixed with F4/80 antibody‑conjugated magnetic beads (Biolegend, 480170) and incubated on ice for 15 minutes. The cell suspension was subsequently placed in a magnetic cell separator (EasySep Magnet, 1800) to isolate the labeled cells. The retained cells were washed three times with separation buffer to obtain the F4/80⁺ macrophage fraction.

(2) Stimulation and RNA extraction: PEMs were stimulated with LPS (100 ng/mL) for 3 hours, and cell pellets were collected for total RNA extraction. Total RNA was extracted using TRIzol reagent (Invitrogen) and treated with DNase I to remove genomic DNA contamination. RNA integrity and purity were verified using an Agilent 2100 Bioanalyzer and NanoDrop spectrophotometer.

(3) Library construction, sequencing and bioinformatics analysis: Library construction and high-throughput sequencing were performed by the Beijing Genomics Institute (BGI) using the DNBSEQ platform, generating 150 bp paired-end reads. After removing low-quality reads and adapter sequences, clean reads were aligned to the mouse reference genome (mm10) using HISAT2 (v2.1.0). Gene expression quantification was performed using featureCounts (v2.0.1). Differential expression analysis was conducted using DESeq2 (v1.30.1), with significantly differentially expressed genes defined as |log₂(Fold Change)| > 1 and adjusted *p*-value (padj) < 0.05. Heatmaps were generated using Z-score-normalized FPKM values, with hierarchical clustering based on Euclidean distance and complete linkage method.

(4) Data availability: The complete RNA-seq raw data and related information have been deposited in the GEO database under the temporary submission ID (PRJNA1271935).

(5) Revised figure legends: The figure legends for revised Fig. 3 have been accordingly expanded. Specifically, the legends for Fig. 3B (heatmap) and Fig. 3C (GSEA analysis) now include detailed descriptions of analytical methods and key parameters.

The complete revised “RNA sequencing” section has been updated in the revised manuscript “Supplementary Materials and Methods”. We thank the reviewer again for this valuable comment, which has significantly improved the rigor and reproducibility of our methods description.

**15. Figure 5 shows a large series of Western blots. How often replicated? Quantified? Apparent the final panel, labelled: (L) PEMs from WT vs. *Timd4* KO mice, supports the statement (line 311) that "as predicted, *Timd4* promoted membrane localization of LKB1 (Fig. 5J-L). Why suddenly look at peritoneal Macs (PEMs) (which do express TIMD4) rather than BMDM? How were PEM isolated? Does the KO alter PEM phenotype? How are the two images different? Quantification?**

**Response:** We would like to thank the reviewer for the careful review and valuable comments on the Western blotting experiments presented in Fig. 5. Regarding your specific concerns about experimental reproducibility, quantitative analysis, cell type selection, PEM isolation method, impact of *Timd4* KO on macrophage phenotypes and image quantification. We address each point below and have made corresponding revisions in the revised manuscript.

(1) Western blotting replicates and quantitative analysis

All Western blotting experiments were independently repeated at least three times, and band intensities were quantified (Supplementary Materials). The quantitative results have been added (revised Fig. 5N, P and Fig. S12B, D). These panels display the gray value ratios of target proteins relative to the internal control (LKB1), along with the number of independent replicates and statistical analysis.

(2) Rationale for using PEMs instead of BMDMs

As the reviewer noted, we used mouse peritoneal resident macrophages (PEMs) rather than bone marrow-derived macrophages (BMDMs) in the original revised Fig. 5L. The reason is as follows: On one hand, PEMs are the commonly used as primary macrophages. On the other hand, TIMD4 expression is significantly higher in tissue-resident macrophages than in BMDMs, particularly in peritoneal macrophages where TIMD4 is highly expressed. This high expression facilitates the clear detection of phenotypic differences caused by *Timd4* deletion in protein localization experiments.

The detailed procedure for PEMs isolation and purification is as follows: C57BL/6J mice were euthanized by CO₂ asphyxiation, and 7–8 mL of ice-cold sterile PBS was injected into the peritoneal cavity. After gentle abdominal massage, the lavage fluid was collected and centrifuged at 1200 rpm for 8 minutes at 4 °C. The cell pellets were resuspended in DMEM medium containing 10 % fetal bovine serum, seeded into culture plates, and incubated at 37 °C with 5 % CO₂ for 4 h. Non-adherent cells were then removed by replacing with fresh medium, and the remaining adherent cells were purified mouse peritoneal resident macrophages.

(3) Effect of *Timd4* knockout on macrophage phenotypes

The *Timd4* deletion does not affect macrophage development or survival. However, our previous studies have demonstrated that *Timd4* deletion significantly affects macrophage polarization and cholesterol metabolism [33–36].

(4) Differences in fluorescence co-localization images and quantitative analysis

In this study, we found that TIMD4 promotes the phosphorylation of LKB1 and AMPK. To elucidate the molecular mechanism underlying this regulation, we conducted subsequent investigations. LKB1 is a constitutively active kinase; when not bound to STRAD/MO25, it is predominantly localized in the nucleus and cannot phosphorylate cytoplasmic AMPK. Its kinase activity depends on the formation of a stable LKB1-STRAD-MO25 heterotrimeric complex with the accessory proteins STRAD and MO25 [37, 38]. Based on this theoretical foundation, we first used AlphaFold 3 to predict the binding of TIMD4 to the LKB1-STRAD-MO25 complex, and verified this interaction through IP experiments, confirming that TIMD4 promotes the formation of the LKB1-STRAD-MO25 complex. Previous studies have shown that after LKB1 forms a complex with STRAD and MO25, it is retained in the cytoplasm, which is a prerequisite for LKB1-mediated AMPK activation [39]. Once in the cytoplasm, LKB1 is not uniformly distributed but is further anchored to specific membrane structures, primarily the plasma membrane and the endoplasmic reticulum/Golgi membrane. This membrane localization is critical for efficient AMPK activation [40]. At the molecular level, LKB1 undergoes farnesylation via its C-terminal CAAX motif, which is the core mechanism for its membrane anchoring. Varghese et al. demonstrated using LKB1 C433S/C433S knock-in mice that LKB1 lacking farnesylation exhibits significantly reduced membrane binding and severely impaired AMPK activation under both basal and various stimulated conditions [41]. This content has been added to the “Discussion” section of the revised manuscript.

Based on this background, we examined the subcellular distribution of LKB1 in the membrane, cytoplasm, and nucleus by immunoblotting. The results showed that TIMD4 overexpression reduced nuclear LKB1 localization, but significantly increased membrane localization (revised Fig. 5E, F). To further validate this conclusion visually, we examined LKB1 subcellular localization by immunofluorescence. Two sets of representative results were presented (revised Fig. 5H–K): (1) HEK293T cells overexpressing control plasmid or full-length TIMD4, detecting exogenous LKB1 membrane localization; (2) primary PEMs from WT versus *Timd4* KO mice, detecting endogenous LKB1 subcellular localization. These two sets of experiments were performed in different experimental systems (overexpression vs. endogenous systems) and different cell types (HEK293T vs. primary macrophages), and yielded consistent results supporting the conclusion that TIMD4 promotes LKB1 localization to the membrane.

For quantitative analysis of immunofluorescence results, we used ImageJ software. Regions of interest were defined by DAPI staining or cell outlines, and fluorescence intensities were measured in the membrane region, cytoplasmic region and nuclear region. The membrane/cytoplasm-to-nucleus fluorescence intensity ratio was calculated. Quantitative statistics showed that TIMD4 deletion resulted in a significantly reduced LKB1 membrane/cytoplasm-to-nucleus fluorescence ratio compared with control groups. These statistical analyses have been added to the revised manuscript (revised Fig. 5I and K).

We sincerely thank the reviewer again for the thorough and professional review. Your comments have been invaluable in improving the rigor and clarity of our study.

**References:**

[33] Wang Y, Wang Y, Ding L, Ren X, Wang B, Wang L, Zhao S, Yue X, Wu Z, Li C, Liang X, Ma C, Gao L. Tim-4 reprograms cholesterol metabolism to suppress antiviral innate immunity by disturbing the Insig1-SCAP interaction in macrophages. Cell Rep. 2022;41(9):111738. doi: 10.1016/j.celrep.2022.111738. PMID: 36450259.

[34] Liu W, Bai F, Wang H, Liang Y, Du X, Liu C, Cai D, Peng J, Zhong G, Liang X, Ma C, Gao L. Tim-4 Inhibits NLRP3 Inflammasome via the LKB1/AMPKα Pathway in Macrophages. J Immunol. 2019; 203(4):990-1000. doi: 10.4049/jimmunol.1900117. PMID: 31263038.

[35] Xu LY, Qi JN, Liu X, Ma HX, Yuan W, Zhao PQ, Liang XH, Xu Y, Wang HX, Xu XY, Wang W, Ma CH, Gao LF. Tim-4 Inhibits NO Generation by Murine Macrophages. PLoS One. 2015; 10(4):e0124771. doi: 10.1371/journal.pone.0124771. PMID: 25905790.

[36] Ding L, Liang Y, Wang Y, Tong Z, Liu W, Tan S, Zhang J, Wang Y, Wu Z, Liang X, Ma C, Gao L. T-cell immunoglobulin- and mucin-domain-containing molecule-4 maintains adipose tissue homeostasis by orchestrating M2 macrophage polarization via nuclear factor kappa B pathway. Immunology. 2023; 168(1):49-62. doi: 10.1111/imm.13555. PMID: 35908188.

[37] Boudeau J, Baas AF, Deak M, Morrice NA, Kieloch A, Schutkowski M, Prescott AR, Clevers HC, Alessi DR. MO25alpha/beta interact with STRADalpha/beta enhancing their ability to bind, activate and localize LKB1 in the cytoplasm. EMBO J. 2003; 22(19):5102-14. doi: 10.1093/emboj/cdg490. PMID: 14517248.

[38] Lizcano JM, Göransson O, Toth R, Deak M, Morrice NA, Boudeau J, Hawley SA, Udd L, Mäkelä TP, Hardie DG, Alessi DR. LKB1 is a master kinase that activates 13 kinases of the AMPK subfamily, including MARK/PAR-1. EMBO J. 2004; 23(4):833-43. doi: 10.1038/sj.emboj.7600110. PMID: 14976552.

[39] Hawley SA, Boudeau J, Reid JL, Mustard KJ, Udd L, Mäkelä TP, Alessi DR, Hardie DG. Complexes between the LKB1 tumor suppressor, STRAD alpha/beta and MO25 alpha/beta are upstream kinases in the AMP-activated protein kinase cascade. J Biol. 2003;2(4):28. doi: 10.1186/1475-4924-2-28. PMID: 14511394.

[40] Dogliotti G, Kullmann L, Dhumale P, Thiele C, Panichkina O, Mendl G, Houben R, Haferkamp S, Püschel AW, Krahn MP. Author Correction: Membrane-binding and activation of LKB1 by phosphatidic acid is essential for development and tumour suppression. Nat Commun. 2022;13(1):1283. doi: 10.1038/s41467-022-28923-3. PMID: 35260598.

[41] Houde VP, Ritorto MS, Gourlay R, Varghese J, Davies P, Shpiro N, Sakamoto K, Alessi DR. Investigation of LKB1 Ser431 phosphorylation and Cys433 farnesylation using mouse knockin analysis reveals an unexpected role of prenylation in regulating AMPK activity. Biochem J. 2014; 458(1):41-56. doi: 10.1042/BJ20131324. PMID: 24295069.

**Revised Fig. 5. TIMD4 promotes LKB1 phosphorylation by stabilizing the LKB1-MO25-STRAD complex.**

(E) Quantification of LKB1 protein levels in whole-cell lysate (WCL) and each subcellular fraction from three independent experiments. (Na, K-ATPase for membrane, β-actin for cytoplasm and H3 histone for nucleus). (F) Subcellular fractionation analysis of LKB1 protein levels in whole-cell lysate (WCL), membrane, cytoplasm and nuclear fractions of HEK293T cells. Western blotting images of LKB1 are shown. (G) Accurate structure prediction of biomolecular interactions with AlphaFold 3. TIMD4 (UniProtKB: Q96H15), MO25 (UniProtKB: Q96H15), STRAD (UniProtKB: Q7RTN6) and LKB1 (UniProtKB: Q15831). (H, I) Immunofluorescence imaging of LKB1 (green) localization in HEK293T cells transfected with empty vector *vs*. *TIMD4* (Scale bars: 10 μm). Quantification of the ratio of the sum of membrane and cytoplasmic fluorescence intensity to nuclear fluorescence intensity in HEK293T cells. (J, K) Immunofluorescence imaging of LKB1 (green) localization in WT and *Timd4* KO PEM (Scale bars: 5 μm). Quantification of the ratio of the sum of membrane and cytoplasmic fluorescence intensity to nuclear fluorescence intensity in RAW264.7 cells.

**Revised Fig. S12. The interaction of different domains of TIMD4 with the LKB1 complex.**

(A) IP assays were performed using anti‑LKB1 antibody in HEK293T cells transfected with pcDNA3.0 (Vector) or *TIMD4* cytosolic domain truncation (Δcyto). Representative Western blotting images of LKB1, MO25 and STRAD are shown. (B) Quantification of MO25 and STRAD protein levels in the IP relative to LKB1 in pcDNA3.0‑ and Δcyto‑expressing cells. (C) IP assays were performed using anti‑LKB1 antibody in HEK293T cells transfected with pUltra vector (control) or soluble *TIMD4* (s*TIMD4*). Representative Western blotting images of LKB1, MO25 and STRAD. (D) Quantification of MO25 and STRAD protein levels in the IP relative to LKB1 in vector‑ and s*TIMD4*‑expressing cells.

**16. For Figure S7, text states that "In addition, Timd4 deficiency also reduced the proportion of M2-type macrophages with tissue-repair functions (Fig. S7), which was consistent with that Timd4 promotes macrophages polarized to be M2 phenotype [21]. Figure S7 actually shows "Representative flow cytometric plots of CD206+ cells gated on CD45+CD11b+CX3CR1+ cells". Where did the cells come from? What is the rationale for the gating? Where is it shown? What tissue repair functions are demonstrated? In any case, the M2 concept is not widely accepted and CD206 is expressed by subsets of resident tissue macrophages.**

**Response:** We sincerely thank the reviewer for the detailed questions and valuable comments. We address each point below.

(1) Cell source

The cells used in this study were derived from mouse colonic tissues. Prior to flow cytometry analysis, colonic tissues were enzymatically digested (Collagenase IV / Dispase / DNase I) to prepare single-cell suspensions, followed by surface staining. This information has been clearly described in the “Flow Cytometry” section of the Materials and Methods.

(2) Gating strategy rationale and supporting literature

CD45 is a pan-leukocyte marker. By excluding CD45⁻cells (e.g., epithelial cells, fibroblasts), all intestinal hematopoietic-derived immune cells were included in the analysis. In flow cytometry analysis of the intestinal lamina propria, CD45⁺ serves as the most fundamental initial gating marker. CD11b (Integrin αM) is widely expressed on the surface of monocytes, macrophages and neutrophils. In the intestinal lamina propria, CD11b gating allows exclusion of lymphocytes (T cells and B cells), thereby restricting the analysis to myeloid cell populations. CX3CR1 is a chemokine receptor highly expressed on colon-resident macrophages and serves as a key marker for distinguishing macrophages from dendritic cells (DCs). Therefore, we used CD45⁺CD11b⁺CX3CR1⁺ gating to enrich colon-resident macrophage populations.

This gating strategy is supported by the following literature:

- Medina-Contreras O et.al. have demonstrated that CX3CR1 is expressed on CD11b⁺F4/80⁺ lamina propria-resident macrophages but not on CD11c⁺CD103⁺ dendritic cells. CX3CR1 knockout mice showed significantly reduced numbers and frequencies of intestinal lamina propria macrophages, indicating an indispensable role of CX3CR1 in maintaining lamina propria macrophage homeostasis [42].
- Bain CC et.al. have identified three distinct CD11b⁺ cell populations among CD45⁺ cells in CX3CR1^+/gfp^ mice: CX3CR1⁻, CX3CR1^int^ and CX3CR1^hi^. CX3CR1^hi^ cells exhibited typical resident macrophage characteristics, expressing high levels of F4/80 and MHCII but lacking CD103, SiglecF, or Ly6C. The CX3CR1^int^ population was highly heterogeneous, with four subsets identified based on MHCII, CD11c, Ly6C and F4/80 expression. Among these, the P1 subset expressed high levels of Ly6C but low levels of F4/80, and lacked MHCII, CD11c, or CD103, consistent with Ly6C^hi^ inflammatory monocytes [43].

(3) M2/CD206 and tissue repair

We fully agree that the reviewer correctly noted the controversy surrounding the M1/M2 classification paradigm. M1/M2 represents a simplified model for describing macrophage polarization; in reality, macrophages exhibit a continuous spectrum from pro-inflammatory to anti-inflammatory states and display high plasticity in response to microenvironmental signals. However, the term M2 remains widely used in the field to describe macrophage functional polarity. Nevertheless, directly equating the proportion of CD206⁺ cells to M2 cells in the original manuscript was indeed insufficiently rigorous. Accordingly, we have revised all related descriptions to CD206⁺ cells in the revised manuscript to avoid conceptual confusion.

Regarding the comment that tissue repair functional experiments were not shown, we also acknowledge this as a limitation of the previous version. The original Fig. S7 only provided flow cytometry phenotypic data without functional evidence. To address this, we supplemented the analysis by examining the expression of tissue repair-related factors *Il10*, *Arg1* and *Tgfb1*. The results showed that *Timd4* deletion significantly reduced the mRNA levels of *Il10*, *Arg1* and *Tgfb1* in colonic tissues compared to controls (revised Fig. S9C), suggesting that *Timd4* deficiency may impair tissue repair capacity. Furthermore, previous studies have demonstrated that CD206⁺ intestinal macrophages promote inflammatory mucosal repair in IBD [44]. IL-4-treated macrophages exhibited a CD206⁺CCL18⁺CD14^low/-^ phenotype and promoted epithelial wound repair through TGF-β secretion, while antagonizing IFN-γ-induced epithelial barrier dysfunction [45]. Taken together, the emerging evidence and our supplemental data suggest that the reduced proportion of CD206⁺ cells in the intestinal lamina propria caused by *Timd4* deletion likely compromises intestinal epithelial repair capacity.

We thank the reviewer for these valuable comments, which have significantly improved the rigor of our study.

**References:**

[41] Medina-Contreras O, Geem D, Laur O, Williams IR, Lira SA, Nusrat A, Parkos CA, Denning TL. CX3CR1 regulates intestinal macrophage homeostasis, bacterial translocation, and colitogenic Th17 responses in mice. J Clin Invest. 2011; 121(12):4787-95. doi: 10.1172/JCI59150. PMID: 22045567.

[43] Bain CC, Scott CL, Uronen-Hansson H, Gudjonsson S, Jansson O, Grip O, Guilliams M, Malissen B, Agace WW, Mowat AM. Resident and pro-inflammatory macrophages in the colon represent alternative context-dependent fates of the same Ly6Chi monocyte precursors. Mucosal Immunol. 2013; 6(3):498-510. doi: 10.1038/mi.2012.89. PMID: 22990622.

[44] Jia DJ, Wang QW, Hu YY, He JM, Ge QW, Qi YD, Chen LY, Zhang Y, Fan LN, Lin YF, Sun Y, Jiang Y, Wang L, Fang YF, He HQ, Pi XE, Liu W, Chen SJ, Wang LJ. Lactobacillus johnsonii alleviates colitis by TLR1/2-STAT3 mediated CD206+ macrophagesIL-10 activation. Gut Microbes. 2022 Jan-Dec;14(1):2145843. doi: 10.1080/19490976.2022.2145843. PMID: 36398889.

[45] Jayme TS, Leung G, Wang A, Workentine ML, Rajeev S, Shute A, Callejas BE, Mancini N, Beck PL, Panaccione R, McKay DM. Human interleukin-4-treated regulatory macrophages promote epithelial wound healing and reduce colitis in a mouse model. Sci Adv. 2020; 6(23): eaba4376. doi: 10.1126/sciadv.aba4376. PMID: 32548267.

**Revised Fig. S9. *Timd4* deficiency reduces CD206⁺ cell proportion and anti‑inflammatory gene expression in colonic tissues.**

(A) Representative flow cytometric plots of CD206^+^ cells gated on CD45^+^CD11b^+^CX3CR1^+^ cells. (B) Flow cytometric quantification of CD206⁺ cells. (C) mRNA levels of *Il10*, *Arg1* and *Tgfb1* in colonic tissues measured by RT-qPCR.

**Reviewer #2:**

**In this manuscript Liu et al present convincing evidence showing that Timd-signaling can ameliorate colitis in the DSS colitis model by reducing the traffic of macrophages to the gut. As such, it enhances the effect of concomitant Vedolizumab administration, another cell traffic inhibitor. However, the data presented stops well short of proving that such signaling would be effect in human disease. Thus, as mentioned at greater length in the specific comments, it is not clearly proven that this treatment approach would be effective in more chronic inflammation than is captured by the acute DSS colitis model since the latter are driven by dendritic cell function that is not inhibited by Timd signaling and signaling pathways that counter Timd signaling activity.**

**Response:** We sincerely thank the reviewer for the positive comments and constructive suggestions. We have carefully read and fully considered your remarks, particularly regarding the translational relevance of our findings to human disease and the limitations of the acute DSS colitis model. We have provided point-by-point responses to each of your specific comments below, and further discussed the implications and limitations of this study. We greatly appreciate your guidance, which will help us further improve the manuscript.

**Specific Comments**

**1. Figure 1A: Timd4 staining of UC tissue is not identified as to location. Is it mainly in the epithelial layer or in the lamina propria? Localization of staining in DSS colitis in Figs N, R-S is likewise unclear.**

**Response:** We thank the reviewer for the detailed questions regarding the staining localization in Fig. 1A and the original Fig. 1N, R–S. We fully understand your concerns about the clarity of tissue staining localization. Below, we provide explanations for the expected expression sites of these molecules and the actual staining patterns observed in our images.

(1) Regarding the localization of TIMD4 in UC tissue in revised Fig. 1A

We have reexamined the images and can confirm that TIMD4-positive signals are predominantly distributed in the mucosal lamina propria, exhibiting a stromal-like staining pattern, with no specific staining observed in the epithelial cell layer. This distribution pattern is entirely consistent with the known expression profile of TIMD4 as a macrophage marker. In the revised manuscript, we have replaced these images with newly acquired representative images.

(2) Regarding the localization of markers in the original Fig. 1N, R–S (Revised Fig. 1R, V)

The original figures 1N, R–S show TUNEL staining and immunofluorescence staining for Mucin2, Claudin, Occludin and ZO‑1, respectively. The tissue and subcellular localization of these molecules is well established. In response to the reviewer’s concern about "unclear localization," we provide below a point-by-point explanation of the expected expression sites and the actual presentation in our images, and we have provided higher-resolution images in the revised manuscript.

- Mucin2: Synthesized and secreted by goblet cells, Mucin2 is the major mucin constituting the intestinal mucus layer. In tissue sections, positive signals should be concentrated in the cytoplasm of goblet cells within the intestinal crypts and in the mucus layer covering the epithelial surface. Our images clearly show strong positive signals in the cytoplasm of goblet cells within the crypts, as well as a continuous mucus layer staining on the epithelial surface, with no abnormal distribution in the stroma or other layers.
- Claudin, Occludin and ZO‑1: These three proteins are core components of the tight junction complex and are specifically localized to the apical region of the lateral membrane of adjacent intestinal epithelial cells. In immunofluorescence staining, they exhibit a continuous linear or punctate pattern along the epithelial cell borders. Our staining results accurately reproduce this typical pattern: positive signals are strictly confined to the apical region of the lateral membrane, appearing as a clear reticular linear distribution in areas with intact intestinal epithelial structure, while signal disruption or attenuation is observed in areas of DSS-induced epithelial injury. This signal distribution exhibits significant site specificity and is fully distinguishable, with no ambiguity in localization.
- TUNEL: TUNEL staining labels apoptotic cells. In colonic tissues, apoptotic cells are primarily localized to the epithelial layer and the lamina propria. In our TUNEL images, the vast majority of positive signals are located in the intestinal epithelial layer and lamina propria, which is consistent with the pathological features of DSS-induced colitis, where intestinal epithelial cell apoptosis predominates.

We thank the reviewer for helping us improve the rigor and readability of our data presentation.

**Revised Fig. 1. *Timd4* deficiency aggravates DSS-induced colitis in mice.**

(A) Immunofluorescence staining images of colonic biopsy specimens from UC patients and healthy controls (normal: n = 4, UC: n = 7; scale bar: 20 μm). R) Cell apoptosis was detected by TUNEL staining, and the fluorescence intensity was quantified. V) Immunofluorescence images of colonic sections with Mucin2, Claudin, Occludin and ZO-1 (Red). Scale bars:100 μm.

**2. DSS colitis data in Figs 1 and 2 are somewhat different: difference in WT vs KO is not significant at 6 days in Figure 1 but is significant at this time point in Figure 2. In general, differences between WT and KO are not very impressive. Were differences seen at a lower DSS dose (such as 2% DSS)? It may be that at high doses of DSS, such as the 3% dose used, one is seeing an inordinately great effect of loss of TIMd4 signaling.**

**Response:** We thank the reviewer for the careful observation that the significance of the difference between groups on day 6 of the DSS colitis model appeared inconsistent between Fig. 1 and Fig. 2. We take this observation very seriously and have reexamined the raw data and analysis workflow for both experiments.

First, we reconfirm the statistical results shown in Fig. 1 (revised Fig. 1O): the difference in body weight change between WT and KO mice on day 6 of DSS induction did not reach statistical significance. However, a significant difference emerged on day 7 (*p* < 0.05), and this significance persisted on days 8 and 9. Therefore, considering the overall body weight change curve, the protective effect of TIMD4 is clear and reproducible in the DSS model.

Regarding the finding that a significant difference appeared as early as day 6 in Fig. 2G, we believe this likely reflects reasonable inter-batch variability. The results shown in revised Fig. 1O and Fig. 2G were derived from two independent animal experiments. Although genetic background, housing conditions, and DSS concentration were strictly controlled, it is difficult to completely eliminate subtle differences between batches, including initial body weight, baseline gut microbiota composition, and sensitivity to DSS. In fact, in the DSS colitis model, a temporal shift of 1–2 days in body weight loss curves between different batches within the same genotype is not uncommon. Importantly, the overall trend between WT and KO groups was entirely consistent across both experiments: KO mice consistently exhibited less body weight loss than WT mice, confirming the reproducibility of the protective effect conferred by *Timd4* deficiency against colitis.

We sincerely thank the reviewer for the valuable comments. The reviewer noted that the severe phenotype observed in *Timd4* KO mice under the high-dose (3 %) DSS model might not be significant under a lower DSS dose, and suggested that we perform an additional experiment using 2 % DSS. To fully address this concern, we conducted the 2 % DSS induction experiment as requested to validate the generalizability and reliability of our findings.

WT and *Timd4* KO mice were fed with 2 % DSS for 7 days, followed by 2 days of normal drinking water (revised Fig. S3A). This regimen was designed to induce a milder form of colitis, thereby allowing a clearer assessment of genotype-dependent differences. The results showed that even under the milder 2 % DSS challenge, *Timd4* KO mice still exhibited significantly more severe disease phenotypes compared to WT controls. On day 9, *Timd4* KO mice showed significantly greater body weight loss than WT mice (revised Fig. S3B). *Timd4* KO mice had significantly higher DAI scores than WT mice, indicating more severe clinical symptoms (revised Fig. S3C). Colonic length was significantly shorter in KO mice than in WT mice (revised Fig. S3D–E). Histopathological assessment showed significantly higher scores in KO mice, characterized by more severe mucosal destruction, crypt loss, and inflammatory cell infiltration (revised Fig. S3F, J). Furthermore, PAS/AB and Mucin2 staining scores were significantly reduced in KO mice, suggesting impaired mucus secretion and goblet cell function (revised Fig. S3G, H, K, L). Expression levels of the tight junction proteins Claudin, Occludin and ZO-1 were also significantly decreased in KO mice, indicating severe disruption of the intestinal epithelial barrier (revised Fig. S3I, M).

In summary, the results of the supplementary 2 % DSS experiment demonstrate that the increased susceptibility of *Timd4* KO mice to DSS-induced colitis is not only evident under high-dose (3 % DSS) conditions but also remains highly significant under the lower 2 % DSS challenge. These data, derived from multiple pathological parameters, collectively confirm the critical role of TIMD4 in maintaining intestinal mucosal homeostasis, and this protective function remains indispensable even under conditions of milder inflammatory injury.

**Revised Fig. S3. *Timd4* deficiency aggravates DSS‑induced acute colitis in a 2 % DSS model.**

(A) Experimental design: 6–8‑week‑old male C57BL/6J mice (WT and *Timd4* KO) were administered 2 % DSS in drinking water for 7 days to induce acute colitis, followed by 2 days of normal water. (B) Body weight changes were assessed daily. Statistics on the rate of weight loss on the day 9. (C) DAI scores were calculated based on body weight loss, stool consistency and rectal bleeding. (D) Representative images and (E) quantitative analysis of colonic length. (F) Representative H&E staining images of colonic tissues. → Inflammation, ☆ Edema, the black dotted lines highlight the crypt. (G) Representative PAS staining and Alcian Blue (AB) staining images of colonic sections. (H, I) Representative immunofluorescence staining images of Mucin2, Claudin, Occludin and ZO‑1. Scale bar: 100 μm. (J) Histological scores based on H&E staining. (K) Histological scores based on PAS/AB staining. (L, M) Quantification of mean fluorescence intensity for Mucin2, Claudin, Occludin and ZO‑1. Data are presented as mean ± SEM (n = 5). Statistical significance was assessed using two‑tailed unpaired Student’s *t*‑test for comparisons between two groups. **p* < 0.05, ***p* < 0.01, ****p* < 0.001.

**3. Deletion of macrophages led to clearly higher histology scores in both WT and KO mice. Also, deletion in WT leads to greater weight loss. Macrophages seem to have a protective effect in the presence and absence of TIMd4 signaling. This anti-inflammatory effect of macrophages that is undermined by Timd signaling may be more important in more chronic models of gut inflammation that are more like human IBD.**

**Response:** We thank the reviewer for this important insight. We agree with reviewer’s points that macrophages indeed exert protective effects in IBD through multiple mechanisms. During the resolution phase of inflammation, macrophages mitigate inflammation by clearing apoptotic neutrophils, thereby preventing secondary necrosis. Macrophages express a variety of surface receptors, including not only the TIM family but also MerTK, CD36, Stabilin-2 and others. These receptors mediate efferocytosis by recognizing "Eat-me" signals released by apoptotic cells, thereby promoting a shift toward an anti-inflammatory phenotype [46]. Furthermore, macrophages are essential for restoring the epithelial barrier. Studies have shown that infiltration of TREM2⁺ macrophages is required for epithelial cell proliferation and colonic mucosal wound healing [47]. Macrophages can also promote epithelial cell proliferation in mice by inducing formyl peptide receptor activation, followed by NOX1 signaling through annexin A1 [48], or by secreting IL-10 to activate the CREB-WISP1 signaling pathway [49]. Thus, the protective role of macrophages in colitis is not limited to TIMD4 expression. Macrophages exert protective effects in both wild-type and *Timd4* KO mice.

We fully agree that chronic colitis models more closely recapitulate the disease progression of human IBD than acute models, and that the anti-inflammatory role of macrophages mediated by TIMD4 signaling may have more critical pathophysiological significance in chronic intestinal inflammation.

To address this point, we established a chronic colitis model by administering 2.5 % DSS to 6–8-week-old female WT and *Timd4* KO mice for four consecutive cycles (each cycle: 5 days of DSS followed by 2 days of normal drinking water) (Fig. Ⅲ A). Our results showed that *Timd4* KO mice exhibited significantly exacerbated colonic inflammation compared to WT mice, as evidenced by markedly shortened colonic length (Fig. Ⅲ B, C), more pronounced body weight loss (Fig. Ⅲ D), and significantly higher DAI scores (Fig. Ⅲ E). Histological assessment (H&E staining) further revealed higher histological scores in the KO group, with more severe mucosal destruction, crypt loss and inflammatory cell infiltration (Fig. Ⅲ F, J). Additionally, *Timd4* deficiency compromised intestinal barrier function: PAS staining, AB staining and Mucin2 immunofluorescence all indicated aggravated impairment of the mucus layer (Fig. Ⅲ G, H, K, L), while immunofluorescence staining for Claudin, Occludin and ZO-1 confirmed disruption of intestinal barrier structural integrity (Fig. Ⅲ I, M).

In summary, the anti-inflammatory effects mediated by macrophages and TIMD4 signaling also play an important role in the chronic colitis model. These findings will be published in detail in our follow-up studies. We again thank the reviewer for this valuable suggestion.

**References:**

[46] Na YR, Stakenborg M, Seok SH, Matteoli G. Macrophages in intestinal inflammation and resolution: a potential therapeutic target in IBD. Nat Rev Gastroenterol Hepatol. 2019;16(9):531-543. doi: 10.1038/s41575-019-0172-4. Epub 2019 Jul 16. PMID: 31312042.

[47] Seno H, Miyoshi H, Brown SL, Geske MJ, Colonna M, Stappenbeck TS. Efficient colonic mucosal wound repair requires Trem2 signaling. Proc Natl Acad Sci U S A. 2009. 106(1):256-61. doi: 10.1073/pnas.0803343106. Epub 2008. PMID: 19109436; PMCID: PMC2629230.

[48] Leoni G, Alam A, Neumann PA, Lambeth JD, Cheng G, McCoy J, Hilgarth RS, Kundu K, Murthy N, Kusters D, Reutelingsperger C, Perretti M, Parkos CA, Neish AS, Nusrat A. Annexin A1, formyl peptide receptor, and NOX1 orchestrate epithelial repair. J Clin Invest. 2013 Jan;123(1):443-54. doi: 10.1172/JCI65831. Epub 2012. PMID: 23241962; PMCID: PMC3533303.

[49] Quiros M, Nishio H, Neumann PA, Siuda D, Brazil JC, Azcutia V, Hilgarth R, O'Leary MN, Garcia-Hernandez V, Leoni G, Feng M, Bernal G, Williams H, Dedhia PH, Gerner-Smidt C, Spence J, Parkos CA, Denning TL, Nusrat A. Macrophage-derived IL-10 mediates mucosal repair by epithelial WISP-1 signaling. J Clin Invest. 2017. 127(9):3510-3520. doi: 10.1172/JCI90229. Epub 2017 Aug 7. PMID: 28783045; PMCID: PMC5669557.

**Fig. Ⅲ. *Timd4* deficiency exacerbates intestinal inflammation and disrupts barrier function in a DSS-induced chronic colitis model.**

(A) Schematic diagram of the chronic colitis model establishment. Female WT and *Timd4* KO mice (6–8 weeks old) were subjected to four consecutive cycles of 2.5 % DSS administration (each cycle: 5 days of DSS followed by 2 days of normal drinking water). (B) Representative images and (C) quantitative analysis of colonic length. (D) Body weight changes were assessed daily. Statistics on the rate of weight loss on the day 8, 10, 12. (E) DAI scores were calculated based on body weight loss, stool consistency and rectal bleeding. Statistics on the rate of DAI on the day 6, 8, 10, 12. (F) Representative H&E staining images of colonic tissues. → Inflammation, ☆ Edema, the black dotted lines highlight the crypt. (G) Representative PAS staining images and AB staining images. (H, I) Representative immunofluorescence staining images of Mucin2, Claudin, Occludin and ZO-1. Scale bars: 100 μm (whole colon sections). (J) Histological scores based on H&E staining. (K) Histological scores based on PAS/AB staining. (L, M) Quantification of mean fluorescence intensity of Mucin2, Claudin, Occludin and ZO-1. Data are presented as mean ± SEM (n = 5). One-way ANOVA was employed to assess differences between multiple groups. Statistical significance was reported as * *p* < 0.05, ** *p* < 0.01, *** *p* < 0.001 and ns, no significance.

**4. Reversal of the effect of loss of Timd4 signaling in KO mice by administration of an CCR2 antagonist is impressive. If this is the case, wouldn't administration of this antagonist be a more feasible treatment of colitis than IR administration of a Timd4 surrogate by nanoparticles?**

**Response:** We sincerely thank the reviewer for acknowledging our use of a CCR2 antagonist to reverse the effect of *Timd4* deletion, and for raising the insightful question regarding its comparison with the TIMD4 mimetic peptide therapeutic strategy. We fully understand the reviewer’s concern: if a CCR2 antagonist can reverse the phenotype, why not directly use it as a therapeutic approach instead of adopting a more complex nanoparticle delivery system? We would like to address this as follows.

First, we emphasize that the primary purpose of using the CCR2 antagonist RS102895 in this study was as a mechanistic validation tool, aimed at definitively demonstrating that the exacerbation of colitis caused by *Timd4* deficiency is dependent on CCR2-mediated monocyte recruitment. Our experimental results showed that the CCR2 antagonist successfully eliminated the phenotypic differences between WT and *Timd4* KO mice, providing strong evidence for the pro-inflammatory mechanism of TIMD4.

However, it is important to note that this does not imply that CCR2 antagonists themselves represent an ideal clinical therapeutic strategy. According to the relevant literature, no CCR2 antagonist has been approved by the FDA or NMPA for the clinical treatment of UC to date. Although some studies have explored the therapeutic potential of CCR2 antagonists in IBD, significant obstacles remain for their clinical translation. Firstly, CCR2 is not only expressed on inflammatory monocytes but also participates in various physiological immune surveillance functions. Long-term systemic inhibition of CCR2 may therefore lead to serious risks of infection or immune dysregulation. Second, existing small-molecule CCR2 antagonists (RS102895) exhibit suboptimal pharmacokinetic profiles, including a short half-life, low bioavailability, and poor targeting capacity to intestinal lesions, often requiring twice-daily administration to maintain efficacy. This dosing regimen is already cumbersome in animal studies and even more challenging to translate into long-term clinical treatment.

In contrast, our designed TIMD4 peptide T4_(365-378)_, delivered via a nanoparticle-targeted system, is enriched at intestinal inflammatory lesions. This approach not only significantly improves local bioavailability but also effectively reduces potential side effects caused by systemic exposure. Importantly, we further conducted comparative experiments to directly evaluate the therapeutic equivalence and dosing advantages of the TIMD4 peptide versus the CCR2 antagonist (revised Fig. 6Q). The results demonstrated that both the T4_(365-378)_-NPs treatment group and the RS102895 treatment group exhibited significant efficacy in ameliorating body weight loss (revised Fig. 6R) and reducing DAI scores (revised Fig. 6S), with no statistically significant difference between the two groups. Both treatments also significantly restored colonic length, with no significant difference between groups (revised Fig. 6T, U). Histopathological assessment showed that both treatments significantly attenuated mucosal destruction, crypt loss and inflammatory cell infiltration, with no significant differences in histological scores (revised Fig. S16A, E). Furthermore, both treatments significantly restored PAS/AB and Mucin2 staining scores, indicating effective improvement in mucus secretion and goblet cell function, with no significant differences between groups (revised Fig. S16B, C, F and G). Regarding intestinal barrier integrity, both treatments significantly upregulated the expression levels of Claudin, Occludin and ZO-1, suggesting effective restoration of intestinal epithelial barrier function, again with no statistically significant differences observed between the two groups (revised Fig. S16D, H).

**Revised Fig. 6. D-mannose-modified HAp-NPs target macrophages to deliver TIMD4 peptide and inhibit NF-κB signaling and chemokine production.**

(Q) Schematic diagram of the acute colitis model and treatment regimen. Acute colitis was induced by administering 3 % DSS for 7 days followed by 2 days of normal drinking water. The CCR2 antagonist group received RS102895 (5 mg/kg, intraperitoneal injection, *i.p.*, twice daily). The T4_(365-378)_-NPs treatment group received the TIMD4 peptide via colonic administration (0.1 mg) on days 1, 4 and 7. (R) Body weight changes were assessed daily. Statistics on the rate of weight loss on the day 9. (S) Body weight, stool consistency, and rectal bleeding were scored and summated to calculate the DAI scores. (T) The quantitative analysis of colonic length. (U) Representative images of colon.

**Revised Fig. S16. TIMD4 peptide T4_(365-378)_ delivered via nanoparticle-targeted system exhibits therapeutic efficacy comparable to CCR2 antagonist in acute DSS-induced colitis.**

(A) Representative H&E staining images of colonic tissues. → Inflammation, ☆ Edema, the black dotted lines highlight the crypt. (B) Representative PAS staining images and AB staining images. (C, D) Representative immunofluorescence staining images of Mucin2, Claudin, Occludin and ZO-1. Scale bars: 100 μm (whole colon sections). (E) Histological scores based on H&E staining. (F) Histological scores based on PAS/AB staining. (G, H) Quantification of mean fluorescence intensity of Mucin2, Claudin, Occludin and ZO-1. Data are presented as mean ± SEM (n = 5). One-way ANOVA was employed to assess differences between multiple groups. Statistical significance was reported as * *p* < 0.05, ** *p* < 0.01, *** *p* < 0.001 and ns, no significance.

**5. If Tim4d signaling is inducing M2 polarization of macrophages this should lead to increased IL-10 secretion in DSS colitis. Is this observed?**

**Response:** We sincerely thank the reviewer for raising this insightful question. If TIMD4 signaling drives M2 polarization of macrophages, increased IL-10 secretion is expected in the DSS-induced colitis model. We fully concur with this logical reasoning, and our experimental data have definitively confirmed this hypothesis.

We measured the mRNA expression levels of M2 macrophage-associated signature genes in colonic tissues of DSS-induced colitis mice. The results demonstrated that, compared with WT control mice, the mRNA expression of *Il-10*, *Tgfb* and *Arg1* was significantly downregulated in colonic tissues of *Timd4* KO mice (revised Fig. S9C). These data indicate that loss of TIMD4 signaling impairs M2 macrophage polarization, which in turn reduces the secretion of anti-inflammatory factors including IL-10. In contrast, TIMD4 signaling is fully functional in WT mice to promote the expression of *Il-10*, *Tgfb* and *Arg1*, thereby maintaining local intestinal immune homeostasis.

We sincerely thank the reviewer for the valuable comments and profound insights on our study.

**Revised Fig. S9. *Timd4* deficiency reduces CD206⁺ cell proportion and anti‑inflammatory gene expression in colonic tissues.**

(A) Representative flow cytometric plots of CD206^+^ cells gated on CD45^+^CD11b^+^CX3CR1^+^ cells. B) Flow cytometric quantification of CD206⁺ cells. (C) mRNA levels of *Il10*, *Arg1* and *Tgfb1* in colonic tissues measured by RT-qPCR. Data are presented as mean ± SEM (n ≥ 3). The unpaired Student’s *t*-test was employed to assess differences between two groups. Statistical significance was reported as * *p* < 0.05, ***p* < 0.01.

**6. It is known that MAPK activation inhibits AMPK. This raises the possibility that in more chronic inflammation than the DSS colitis studied here but more like human UC, MAPK activation would negate the Timd signaling effect? Timd signaling effects need to be tested in a more chronic model of gut inflammation to substantiate its clinical value.**

**Response:** We sincerely thank you for your constructive comments on our study. Your proposal that MAPK activation may inhibit AMPK and further affect the role of the TIMD4 signaling pathway in chronic inflammation is highly insightful. In response to your suggestion that “the effect of TIMD4 signaling should be verified in a chronic inflammatory model to support its clinical value”, we have added the experimental results of *Timd4* knockout in the chronic colitis model in the response (Fig. Ⅲ). This experiment directly addresses your concern: we utilized a DSS-induced chronic colitis model that more closely resembles human UC, and observed that *Timd4* deficiency significantly aggravates chronic intestinal inflammation. This result is consistent with our findings in the acute DSS model, indicating that TIMD4 signaling still exerts a protective effect under chronic inflammatory conditions.

Existing literature documents complex reciprocal regulation between the AMPK and MAPK signaling pathways. In general, AMPK activation is known to inhibit inflammation-related MAPK signaling. However, there is currently a lack of clear direct evidence as to whether sustained activation of the MAPK pathway directly inhibits AMPK activity in the context of chronic inflammation. Given that we have already observed that the protective effect of TIMD4 signaling remains robust in our chronic colitis model, we conclude that even if potential negative regulation of AMPK by MAPK exists, this regulation is insufficient to completely counteract the pro-inflammatory effect induced by *Timd4* deletion under our current experimental conditions. We will take this important mechanistic question as a key focus for future research and further explore it in subsequent studies. Thank you again for your valuable comments.

**7. Treatment of DSS colitis by IR administration of Timd peptide-expressing NPs alone is not more effective than Vedo alone. However, it acts in synergy with Vedo. Does it enhance the effect of somewhat more powerful agents such as anti-TNFa or anti-IL-12p40?**

**Response:** We sincerely thank the reviewer for the valuable comments. In response to your question regarding “whether T4_(356-378)_-NPs could also enhance the efficacy of more potent therapeutic agents such as anti-TNFα or anti-IL-12p40,” we have added the results of a combination therapy experiment using T4_(356-378)_-NPs and anti-TNFα in the revised manuscript.

In this study, 6–8-week-old female C57BL/6J mice were randomly assigned to the following groups: (1) Water group: received normal drinking water for 9 consecutive days; (2) DSS group: received 3 % DSS in drinking water for 7 days to induce acute colitis, followed by 2 days of normal water; (3) anti-TNFα treatment group (Anti-mouse TNFα-InVivo, Selleck, XT3.11): intraperitoneal injection at 4 mg/kg on days 1, 4 and 8 after colitis induction; (4) Isotype control IgG group (Ultra-LEAF™ Purified Rat IgG2a, BioLegend, 400544): intraperitoneal injection at 4 mg/kg on days 1, 4 and 8 after colitis induction; (5) T4_(356-378)_-NPs group: intrarectal administration at 0.1 mg on days 2, 4 and 7 after colitis induction; (6) Combination therapy group: received both anti-TNFα and T4_(356-378)_-NPs according to the above regimens (revised Fig. 8A).

The results demonstrated that T4_(356-378)_-NPs significantly enhanced the therapeutic efficacy of anti-TNFα. Compared with the anti-TNFα monotherapy group, the combination therapy group exhibited significantly reduced body weight loss (revised Fig. 8B), significantly lower DAI scores (revised Fig. 8C), and significantly restored colonic length (revised Fig. 8D, E). Histopathological analysis revealed that the combination therapy markedly attenuated mucosal destruction, crypt loss and inflammatory cell infiltration (revised Fig. 8F, J). PAS/AB staining and Mucin2 staining indicated better recovery of mucus secretion and goblet cell function (revised Fig. 8G, H, K, L). Furthermore, the expression levels of the tight junction proteins Claudin, Occludin and ZO-1 were significantly upregulated (revised Fig. 8I, M).

In summary, T4_(356-378)_-NPs not only synergize with anti-α4β7 therapy but also significantly enhance the efficacy of anti-TNFα treatment in the DSS-induced colitis model, further supporting the potential of the TIMD4 signaling pathway as a therapeutic target for inflammatory bowel disease. We once again thank the reviewer for the profound insights, which have greatly improved our study.

**Revised Fig. 8 T4_(356‑378)_‑NPs enhance the therapeutic efficacy of anti‑TNFα in DSS‑induced acute colitis.**

(A) Experimental scheme of combination therapy. 6–8‑week‑old female C57BL/6J mice were randomly assigned to six groups: (1) Water group; (2) DSS group; (3) anti‑TNFα group (anti‑mouse TNFα antibody, XT3.11, Selleck, 4 mg/kg, *i.p.*, on day 1, 4, 8); (4) Isotype control IgG group (Ultra‑LEAF™ Purified Rat IgG2a, BioLegend, 400544, 4 mg/kg, *i.p.* on day 1, 4, 8); (5) T4_(356‑378)_‑NPs group (0.1 mg, *p.r.* on day 2, 4, 7); (6) Combination therapy group (received both anti‑TNFα and T4_(356‑378)_‑NPs according to the above regimens). B) Body weight changes were assessed daily. Statistics on the rate of weight loss on the day 9. (C) Representative images and (D) quantitative analysis of colonic length. (E) Body weight, stool consistency and rectal bleeding were scored and summated to calculate the DAI scores. (F) Representative H&E staining images of colonic tissues. → Inflammation, ☆ Edema, the black dotted lines highlight the crypt. (G) Representative PAS staining images and AB staining images. (H, I) Representative immunofluorescence staining images of Mucin2, Claudin, Occludin and ZO-1. Scale bars: 100 μm. J) Histological scores based on H&E staining. (K) Histological scores based on PAS/AB staining. (L, M) Quantification of mean fluorescence intensity of Mucin2, Claudin, Occludin and ZO-1. Data are presented as mean ± SEM (n = 5). One-way ANOVA was employed to assess differences between multiple groups. Statistical significance was reported as * *p* < 0.05, ** *p* < 0.01, *** *p* < 0.001 and ns, no significance.

**8. Does it enhance Vedo when both Vedo and peptide are initiated 4 days after DSS colitis is initiated.**

**Response:** We sincerely thank the reviewer for raising this critical question with significant clinical translational implications. The reviewer pointed out that our original dosing regimen (days 1, 4 and 7) was initiated during the early stages of DSS-induced colitis (even before disease onset), which does not reflect clinical practice, as the vast majority of ulcerative colitis patients receive treatment only after entering the active disease phase. We fully concur with this perspective. To more accurately recapitulate the clinical treatment scenario, we performed additional experiments using a therapeutic dosing regimen, in which the initiation of both T4_(356-378)_-NPs and anti-α4β7 treatment was delayed until day 4 post-DSS induction (i.e., the active disease phase).

In this study, 6–8-week-old female C57BL/6J mice were randomly assigned to the following groups: (1) Water group: received normal drinking water for 9 consecutive days; (2) DSS group: received 3 % DSS in drinking water for 7 days to induce acute colitis, followed by 2 days of normal water; (3) anti-α4β7 treatment group (Purified anti-mouse LPAM-1 (Integrin α4β7) antibody, BioLegend, clone DATK32): intraperitoneal injection at 50 μg on days 4 and 7; (4) Isotype control IgG group (Ultra-LEAF™ Purified Rat IgG2a, BioLegend, 400544): intraperitoneal injection at 50 μg on days 4 and 7; (5) T4_(356-378)_-NPs group: intrarectal administration at 0.1 mg on days 4 and 7; (6) Combination therapy group: received both anti-α4β7 and T4_(356-378)_-NPs according to the above regimens (revised Fig. S18A).

The results demonstrated that T4_(356-378)_-NPs significantly enhanced the therapeutic efficacy of anti-α4β7, even in this therapeutic model. Compared with the anti-α4β7 monotherapy group, the combination therapy group exhibited significantly reduced body weight loss (revised Fig. S18B), significantly lower DAI scores (revised Fig. S18E), and significantly restored colonic length (revised Fig. S18C, D). Histopathological analysis revealed that combination therapy markedly attenuated mucosal damage, crypt loss, and inflammatory cell infiltration (revised Fig. S18F, J). PAS/AB staining and Mucin2 immunofluorescence staining indicated more pronounced improvement in mucus secretion and goblet cell function in the combination therapy group (revised Fig. S18G, H, K, L). Furthermore, the expression levels of the tight junction proteins Claudin, Occludin and ZO-1 were also significantly upregulated (revised Fig. S18I, M).

Collectively, these results indicate that T4_(356-378)_-NPs significantly enhance the efficacy of anti-α4β7 even in a therapeutic treatment model, further supporting the clinical translational potential of targeting the TIMD4 signaling pathway in the treatment of inflammatory bowel disease. We once again thank the reviewer for the profound insights, which have greatly strengthened the design of this study and brought it closer to clinical reality.

**Revised Fig. S18. T4_(356‑378)_-NPs enhance the therapeutic efficacy of anti‑α4β7 antibody in colitis therapeutic model.**

(A) Experimental protocol. Female C57BL/6J mice (6–8 weeks old) were randomly assigned to six groups: Water group (normal drinking water for 9 days); DSS group (3 % DSS for 7 days, followed by normal water for 2 days); anti‑α4β7 group (clone DATK32, intraperitoneal injection, 50 μg, on days 4 and 7); Isotype control IgG group (rat IgG2a, intraperitoneal injection, 50 μg, on days 4 and 7); T4_(356‑378)_-NPs group (intrarectal administration, 0.1 mg, days 4 and 7); Combination group (anti‑α4β7 + T4_(356‑378)_-NPs according to the above regimens). B) Body weight changes were assessed daily. Statistics on the rate of weight loss on the day 9. (C) Representative images and (D) quantitative analysis of colonic length. (E) Body weight, stool consistency and rectal bleeding were scored and summated to calculate the DAI scores. (F) Representative H&E staining images of colonic tissues. → Inflammation, ☆ Edema, the black dotted lines highlight the crypt. (G) Representative PAS staining images and AB staining images. (H, I) Representative immunofluorescence staining images of Mucin2, Claudin, Occludin and ZO-1. Scale bars: 100 μm (whole colonic sections). (J) Histological scores based on H&E staining. (K) Histological scores based on PAS/AB staining. (L, M) Quantification of mean fluorescence intensity of Mucin2, Claudin, Occludin and ZO-1. Data are presented as mean ± SEM (n = 5). One-way ANOVA was employed to assess differences between multiple groups. Statistical significance was reported as * *p* < 0.05, ** *p* < 0.01, *** *p* < 0.001 and ns, no significance.

**9. How long does the effect of Timd signaling last? Does it ameliorate DSS colitis when administered several days prior to DSS administration?**

**Response:** We sincerely thank the reviewer for the valuable comments. Your concern regarding the duration of the TIMD signaling pathway effect and its prophylactic potential when administered prior to DSS treatment is highly significant. To address this, we performed an additional experiment using a prophylactic dosing regimen, in which T4_(356-378)_-NPs were administered prior to DSS-induced colitis to evaluate their preventive efficacy.

The experimental protocol was as follows: T4_(356-378)_-NPs (0.1 mg) were administered by intrarectal instillation one day before the start of 3 % DSS in drinking water; mice were then given 3 % DSS in drinking water for 7 consecutive days to induce acute colitis, followed by normal drinking water for 2 days (revised Fig. 6V). The results demonstrated that even in this prophylactic model, T4_(356-378)_-NPs effectively ameliorated DSS-induced colonic inflammation. Compared with the DSS model group, mice in the T4_(356-378)_-NPs pretreatment group exhibited significantly reduced body weight loss (revised Fig. 6W), significantly lower DAI scores (revised Fig. 6X), and significantly restored colonic length (revised Fig. 6Y, Z). Histopathological analysis showed that prophylactic administration of T4_(356-378)_-NPs markedly attenuated mucosal damage, crypt loss and inflammatory cell infiltration (revised Fig. S17A, E). PAS/AB staining and Mucin2 immunofluorescence staining revealed significantly improved mucus secretion and goblet cell function in the prophylactic group (revised Fig. S17B, C, F and G). Furthermore, the expression levels of the tight junction proteins Claudin, Occludin and ZO-1 were also significantly upregulated (revised Fig. S17D, H).

Collectively, these results indicate that T4_(356-378)_-NPs not only enhance the efficacy of therapeutic agents such as anti-α4β7 or anti-TNFα in treatment models, but also exhibit a pronounced anti-inflammatory effect when administered prophylactically. This suggests that the protective effect of the TIMD4 signaling pathway persists for at least up to the peak of DSS-induced inflammation (day 9 post-administration), highlighting its considerable prophylactic potential. This finding further supports the clinical translational value of targeting the TIMD4 signaling pathway in the treatment of inflammatory bowel disease. We once again thank the reviewer for the insightful suggestions, which have enabled us to more comprehensively evaluate the duration of action and application potential of T4_(356-378)_-NPs, thereby greatly strengthening this study.

**Revised Fig. 6. D-mannose-modified HAp-NPs target macrophages to deliver TIMD4 peptide and inhibit NF-κB signaling and chemokine production.**

(V) Experimental protocol: Mice received a single intrarectal instillation of T4_(356‑378)_-NPs (0.1 mg) one day before the start of 3 % DSS in drinking water; DSS was administered for 7 consecutive days, followed by normal water for 2 days. (W) Body weight changes were assessed daily. Statistics on the rate of weight loss on the day 8 and day 9. (X) Body weight, stool consistency and rectal bleeding were scored and summated to calculate the DAI scores. (Y) Representative images of colon. (Z) The quantitative analysis of colonic length.

**Revised Fig. S17. Prophylactic administration of T4_(356-378)_-NPs ameliorates DSS‑induced acute colitis in mice.**

(A) Representative H&E staining images of colonic tissues. → Inflammation, ☆ Edema, the black dotted lines highlight the crypt. (B) Representative PAS staining images and AB staining images. (C, D) Representative immunofluorescence staining images of Mucin2, Claudin, Occludin and ZO-1. Scale bars: 100 μm (whole colonic sections). (E) Histological scores based on H&E staining. (F) Histological scores based on PAS/AB staining. (G, H) Quantification of mean fluorescence intensity of Mucin2, Claudin, Occludin and ZO-1. Data are presented as mean ± SEM (n = 5). One-way ANOVA was employed to assess differences between multiple groups. Statistical significance was reported as * *p* < 0.05, ** *p* < 0.01, *** *p* < 0.001 and ns, no significance.
