# Supplementary material for: A macrophage-targeted TIMD4 Peptide-Nanoparticle attenuates colitis symptoms and monocyte recruitment in a mouse model
Source: PLoS Biol. 2026 Oct 21;24(10):e3004056. doi: 10.1371/journal.pbio.3004056 (PMC13686490; doi:10.1371/journal.pbio.3004056)
Supplement: Supplementary file 3 [file pbio.3004056.s032.docx]

**Response to Reviewers**

Reviewer #2:

> The authors have provided positive response to each of the comments of the 2nd review including the comment of both reviewers. In many cases this included extensive new data.

**Response:** We sincerely thank the reviewer for the positive assessment of our revisions. We greatly appreciate the constructive comments provided throughout the review process, which have helped us strengthen the experimental evidence and improve the rigor and clarity of the manuscript.

> Reviewer #3:

> 1) The authors show that TIMD4 has a protective role in DSS colitis. They claim that the underlying mechanism is the increased production of cytokines and chemokines (especially CCL2) by *Timd4*-deficient macrophages. However, they provide only indirect evidence e.g. by treating WT and *Timd4*-KO mice with clodronate liposomes or a CCR2 antagonist. If *Timd4*-deficient macrophages would be the main disease drivers, then depletion of macrophages with clodronate should improve colitis in *Timd4*-KO mice, which is not the case. Of course, one could argue that macrophages also have a protective role, independent of their expression of Timd4 and then the depletion of these macrophages would not improve colitis even in *Timd4*-KO mice. Unfortunately, no data in this direction are provided.

**Response:** We thank the reviewer for highlighting this important issue. We agree that the worsening of colitis following clodronate-liposome treatment is not consistent with interpreting *Timd4*-deficient macrophages as the principal disease-driving population. We have therefore revised the manuscript to clarify the interpretation of the macrophage-depletion and CCR2-antagonist experiments and to avoid attributing the aggravated colitis in *Timd4* KO mice exclusively to increased cytokine or chemokine production.

We have clarified this interpretation in the revised Results and Discussion.

Results: “Macrophage depletion exacerbated colitis in both WT and *Timd4* KO mice, as reflected by greater colon shortening and higher histopathological scores. Following depletion, no statistically significant differences in colon length, body weight loss or histopathological scores were detected between WT and *Timd4* KO mice (Fig. 2E–G, J, K). Together with the increased TIMD4 expression in colonic macrophages following DSS treatment, these findings support the involvement of macrophages in TIMD4-associated protection against DSS-induced colitis.”

Discussion: “Macrophage depletion aggravated DSS-induced colitis in both WT and *Timd4* KO mice, consistent with a protective role for macrophages in this model. Following macrophage depletion, no statistically significant differences in colon length, body weight loss or histopathological scores were detected between the two genotypes. These findings suggest that the protective effects of macrophages during DSS-induced colitis may be mediated, at least in part, by TIMD4.”

> 2) The evidence that CCL2 is the main disease driver in Timd4-KO mice is also very thin and indirect. Just the fact that treatment with an CCR2-antagonst induces somewhat more improvement in Timd4-KO mice than in WT mice, does not mean very much.

**Response:** Thanks for the reviewer’s helpful comment. The CCR2-antagonist experiment was also reinterpreted more cautiously in the revised Results and Discussion.

Results: “In the absence of RS102895, *Timd4* KO mice exhibited greater body weight loss, higher DAI scores, more pronounced colon shortening and higher levels of IL-6, IL-1β and TNF-α in colonic tissues than those of WT mice. Following RS102895 treatment, no statistically significant differences in these parameters were observed between WT and *Timd4* KO mice (Fig. 4B–F).”

Discussion: “Pharmacological blockade of CCR2 attenuated colitis and reduced colonic monocyte accumulation in *Timd4* KO mice, suggesting that CCR2-dependent monocyte recruitment may contribute to the aggravated colitis associated with *Timd4* deficiency.”

These revisions more accurately distinguish the overall protective contribution of macrophages from the specific contribution of TIMD4-associated chemokine regulation and CCR2-dependent monocyte recruitment.

> 3) There a lot of other potential mechanisms how Timd4 deficiency could exacerbate colitis, e.g. due to the accumulation of apoptotic cells (see Fig. 1 R, S) or missing interaction of TIMD4 with T cells (PMID: 18367551).

**Response:** We thank the reviewer for highlighting these alternative mechanisms. We agree that altered chemokine production may not fully account for the aggravated colitis observed in *Timd4* KO mice. We have therefore expanded the Discussion to acknowledge the potential contribution of impaired apoptotic cell clearance. The corresponding discussion has been added to the revised manuscript as follows: “In addition, we observed a significant increase in apoptotic cell accumulation in the colonic tissues of *Timd4* KO mice. Given the established role of TIMD4 as an efferocytic receptor, this finding suggests that *Timd4* deficiency may also impair apoptotic cell clearance”.

> 4) Nevertheless, I would not be overly critical in this regard, as the authors provide at least some potential mechanisms. However, I would request that they modify their rather strong statements (e.g. line 482 ff "In the present study, we demonstrate that TIMD4 exerts a protective effect against IBD by precisely modulating controls the production of Ccl2 and additional chemokines via the LKB1/AMPK/NF-κB signaling axis, which effectively curtails inhibits inflammatory monocyte infiltration and prevents local immune overactivation, thus exerting a protective effect against IBD.")

**Response:** We thank the reviewer for this important comment. We agree that the original statement was overly definitive given the evidence presented. We have therefore revised the text to distinguish the observed associations from the proposed mechanism and to limit the conclusion to the DSS-induced colitis model. The revised sentence reads:

“Collectively, these data suggest that TIMD4 may protect against DSS-induced colitis, at least in part, by regulating chemokine production via the LKB1–AMPK–NF-κB axis, thereby potentially limiting monocyte infiltration and local immune responses.”

In addition, we carefully reviewed the entire manuscript and revised other statements where necessary to avoid overly strong causal or mechanistic interpretations. All corresponding revisions are highlighted in red in the revised manuscript.

> 5) The protective effects of nanoparticles coated with a Timd4 peptide T4(365-378)-NPs, remains a bit enigmatic to me. After uptake of nanoparticles by macrophages, how should the nanoparticles get access to the intracellular space to induce signal transduction in macrophages? Would be great to see data (not only for p-P65 like in Fig. 6G, H, but also more relevant read-outs). E.g. does treatment with nanoparticles reduce the LPS-induced expression of CCL2 and other cytokines / chemokines. However, I would not consider this as a mandatory point.

**Response:** We thank the reviewer for raising this important question. To clarify a plausible route for intracellular delivery, we have added relevant references and the following discussion:

“Previous studies have shown that mannose modification can promote nanoparticle uptake by macrophages through mannose receptor-mediated endocytosis [44]. Following internalization, the acidic endosomal environment may promote the dissolution of hydroxyapatite and other calcium phosphate-based carriers, thereby facilitating the intracellular release of their cargo [45].”

We acknowledge that the intracellular trafficking and cargo release of T4_(365–378)_-NPs were not directly examined in the present study. Accordingly, we present the above process as a plausible delivery mechanism rather than a mechanism established by our data.

In addition to P65 phosphorylation, we assessed downstream inflammatory mediators in vivo. Treatment with TIMD4-derived peptide nanoparticles significantly reduced the colonic levels of monocyte-recruiting chemokines and pro-inflammatory cytokines in both *Timd4* KO and WT mice (Figs. S14A, B and S15G, H). Although these experiments do not replace direct analysis in LPS-stimulated macrophages in vitro, we provide in vivo evidence that nanoparticle treatment is associated with suppression of the relevant inflammatory mediators in the DSS-induced colitis model.

> Minor points:

> 1) Line 200: Fig. 1 H-K instead of Fig. 1 D-G

**Response:** Thank you for identifying this incorrect figure citation. We are terribly sorry for our carelessness. We have replaced “Fig. 1D–G” with “Fig. 1H–K” at line 203.

> 2) Line 206: Please write: increased number of apoptotic cells instead of "increased apoptosis". Most likely apoptotic cells are less efficiently cleared in *Timd4* KO mice. Fig 1R-S instead of Fig. 1S

**Response:** Thank you for this important clarification. We have replaced “increased apoptosis” with “increased number of apoptotic cells” at line 210, which more accurately describes the observed result without implying increased induction of apoptosis. In addition, the figure citation has been corrected from “Fig. 1S” to “Fig. 1R, S.”

> 3) Line 211: In addition instead of “Meanwhile”

**Response:** Thank you for this suggestion. We have replaced “Meanwhile” with “In addition” at line 215. We also replaced definitive terms that could exceed the scope of the available evidence, including “demonstrate” “confirm” “establish” and “critical driver” with more appropriately qualified terms such as “show” “suggest” “support” “is associated with”, “observe” and “contributes to” et.al. All revisions are highlighted in red in the revised manuscript.

> 4) Unclear if treatment with T4_(365-378)_-NPs in combination with an CCR2-anatgonist, anti-α4β7 or anti-TNFα was done in WT or *Timd4* KO mice.

**Response:** Thank you for requesting this clarification. The comparative experiment involving T4_(365–378)_-NPs and the CCR2 antagonist, as well as the combination experiments involving T4_(365–378)_-NPs with either anti-α4β7 or anti-TNF-α antibodies, were all performed in WT mice. We have now explicitly specified the mouse genotype in the **Materials and Methods**, **Results** and corresponding **Figures** and **Figure legends**.
