# Supplementary material for: A macrophage-targeted TIMD4 Peptide-Nanoparticle attenuates colitis symptoms and monocyte recruitment in a mouse model
Source: PLoS Biol. 2026 Oct 21;24(10):e3004056. doi: 10.1371/journal.pbio.3004056 (PMC13686490; doi:10.1371/journal.pbio.3004056)
Supplement: Peer review history

# Editor report: Decision Letter 0

**DOI:** 10.1371/journal.pbio.3004056.r001

**Authors:** Melissa Vazquez Hernandez

26 Nov 2025

Dear Dr Gao,

Thank you for submitting your revised manuscript entitled "Timd4 Peptide-Nanoparticle Targets Macrophages to Ameliorate Colitis via MO25-STRAD-LKB1 Complex Stabilization and Attenuated Monocyte Recruitment" for consideration as a Research Article by PLOS Biology.

Your manuscript has now been evaluated by the PLOS Biology editorial staff as well as by an academic editor with relevant expertise and I am writing to let you know that we would like to send your submission out for external peer review.

However, before we can send your manuscript to reviewers, we need you to complete your submission by providing the metadata that is required for full assessment. To this end, please login to Editorial Manager where you will find the paper in the 'Submissions Needing Revisions' folder on your homepage. Please click 'Revise Submission' from the Action Links and complete all additional questions in the submission questionnaire.

Once your full submission is complete, your paper will undergo a series of checks in preparation for peer review. After your manuscript has passed the checks it will be sent out for review. To provide the metadata for your submission, please Login to Editorial Manager (https://www.editorialmanager.com/pbiology) within two working days, i.e. by Nov 28 2025 11:59PM.

If your manuscript has been previously peer-reviewed at another journal, PLOS Biology is willing to work with those reviews in order to avoid re-starting the process. Submission of the previous reviews is entirely optional and our ability to use them effectively will depend on the willingness of the previous journal to confirm the content of the reports and share the reviewer identities. Please note that we reserve the right to invite additional reviewers if we consider that additional/independent reviewers are needed, although we aim to avoid this as far as possible. In our experience, working with previous reviews does save time.

If you would like us to consider previous reviewer reports, please edit your cover letter to let us know and include the name of the journal where the work was previously considered and the manuscript ID it was given. In addition, please upload a response to the reviews as a 'Prior Peer Review' file type, which should include the reports in full and a point-by-point reply detailing how you have or plan to address the reviewers' concerns.

During the process of completing your manuscript submission, you will be invited to opt-in to posting your pre-review manuscript as a bioRxiv preprint. Visit http://journals.plos.org/plosbiology/s/preprints for full details. If you consent to posting your current manuscript as a preprint, please upload a single Preprint PDF.

Feel free to email us at plosbiology@plos.org if you have any queries relating to your submission.

Kind regards,

Melissa

Melissa Vazquez Hernandez, Ph.D.

Associate Editor

PLOS Biology

mvazquezhernandez@plos.org

# Peer review: Decision Letter 1

**DOI:** 10.1371/journal.pbio.3004056.r002

**Authors:** Melissa Vazquez Hernandez

7 Jan 2026

Dear Dr Gao,

Thank you for your patience while your manuscript "Timd4 Peptide-Nanoparticle Targets Macrophages to Ameliorate Colitis via MO25-STRAD-LKB1 Complex Stabilization and Attenuated Monocyte Recruitment" was peer-reviewed at PLOS Biology. Your manuscript has been evaluated by the PLOS Biology editors, an Academic Editor with relevant expertise, and by two independent reviewers.

As you will see in the reviewer reports, although the reviewers acknowledge the potential interest in your findings, they have also raised a substantial number of crucial concerns. Based on their specific comments and following discussion with the Academic Editor, it is clear that a substantial amount of work would be required to meet the criteria for publication in PLOS Biology. Given our and the reviewer interest in your study, we would be open to inviting a comprehensive revision of the work; however, this would need to thoroughly address all the reviewers' comments in full, specially since reviewer #2 suggests rejection. A successful revision will need to resolve issues related to the experimental rigor necessary to support the conclusions, such as lack of convincing and validated evidence for TIMD4 expression and mechanism, major methodological and interpretive deficiencies, overstatement of therapeutic relevance, and failure to bridge mouse DSS findings to human biology.

IMPORTANT: after discussion with the Academic Editor and the reviewers, we will look for a third reviewer upon submission of the revision.

Given the extent of revision that would be needed, we cannot make a decision about publication until we have seen the revised manuscript and your response to the reviewers' comments. Your revised manuscript would need to be seen by the reviewers again, but please note that we would not engage them unless their main concerns have been addressed.

We appreciate that these requests represent a great deal of extra work, and we are willing to relax our standard revision time to allow you 6 months to revise your study. Please email us (plosbiology@plos.org) if you have any questions or concerns, or envision needing a (short) extension.

In addition to these revisions, you will need to complete some formatting changes, which you will receive in a follow up email. A member of our team will be in touch with a set of requests shortly.

We appreciate that these requests represent a great deal of extra work, and we are willing to relax our standard revision time to allow you 6 months to revise your study. Please email us (plosbiology@plos.org) if you have any questions or concerns, or envision needing a (short) extension.

At this stage, your manuscript remains formally under active consideration at our journal; please notify us by email if you do not intend to submit a revision so that we may withdraw it.

**IMPORTANT - SUBMITTING YOUR REVISION**

Your revisions should address the specific points made by each reviewer. Please submit the following files along with your revised manuscript:

1. A 'Response to Reviewers' file - this should detail your responses to the editorial requests, present a point-by-point response to all of the reviewers' comments, and indicate the changes made to the manuscript.

*NOTE: In your point by point response to the reviewers, please provide the full context of each review. Do not selectively quote paragraphs or sentences to reply to. The entire set of reviewer comments should be present in full and each specific point should be responded to individually, point by point.

You should also cite any additional relevant literature that has been published since the original submission and mention any additional citations in your response.

2. In addition to a clean copy of the manuscript, please also upload a 'track-changes' version of your manuscript that specifies the edits made. This should be uploaded as a "Revised Article with Changes Highlighted " file type.

*Resubmission Checklist*

When you are ready to resubmit your revised manuscript, please refer to this resubmission checklist: https://plos.io/Biology_Checklist

To submit a revised version of your manuscript, please go to https://www.editorialmanager.com/pbiology/ and log in as an Author. Click the link labelled 'Submissions Needing Revision' where you will find your submission record.

Please make sure to read the following important policies and guidelines while preparing your revision:

*Published Peer Review*

Please note while forming your response, if your article is accepted, you may have the opportunity to make the peer review history publicly available. The record will include editor decision letters (with reviews) and your responses to reviewer comments. If eligible, we will contact you to opt in or out. Please see here for more details:

https://blogs.plos.org/plos/2019/05/plos-journals-now-open-for-published-peer-review/

*PLOS Data Policy*

Please note that as a condition of publication PLOS' data policy (http://journals.plos.org/plosbiology/s/data-availability) requires that you make available all data used to draw the conclusions arrived at in your manuscript. If you have not already done so, you must include any data used in your manuscript either in appropriate repositories, within the body of the manuscript, or as supporting information (N.B. this includes any numerical values that were used to generate graphs, histograms etc.). For an example see here: http://www.plosbiology.org/article/info%3Adoi%2F10.1371%2Fjournal.pbio.1001908#s5

*Blot and Gel Data Policy*

We require the original, uncropped and minimally adjusted images supporting all blot and gel results reported in an article's figures or Supporting Information files. We will require these files before a manuscript can be accepted so please prepare them now, if you have not already uploaded them. Please carefully read our guidelines for how to prepare and upload this data: https://journals.plos.org/plosbiology/s/figures#loc-blot-and-gel-reporting-requirements

*Protocols deposition*

To enhance the reproducibility of your results, we recommend that if applicable you deposit your laboratory protocols in protocols.io, where a protocol can be assigned its own identifier (DOI) such that it can be cited independently in the future. Additionally, PLOS ONE offers an option for publishing peer-reviewed Lab Protocol articles, which describe protocols hosted on protocols.io. Read more information on sharing protocols at https://plos.org/protocols?utm_medium=editorial-email&utm_source=authorletters&utm_campaign=protocols

Thank you again for your submission to our journal. We hope that our editorial process has been constructive thus far, and we welcome your feedback at any time. Please don't hesitate to contact us if you have any questions or comments.

Sincerely,

Melissa

Melissa Vazquez Hernandez, Ph.D.

Associate Editor

PLOS Biology

mvazquezhernandez@plos.org

-----------------------------------------

REVIEWERS' COMMENTS

-----------------------------------------

Reviewer #1:

¬This study fails to provide compelling evidence to support the definitive statement in the title or the mechanism proposed in the summary diagram.

The introduction and discussion consistently confuses mouse and human monocyte/macrophage biology. The species needs to clear at every point. For example, Ly6C is a marker for monocyte subsets only in mouse and F4/80 is not used as a marker in humans. The authors should employ the convention for naming genes and proteins. Timd4 for gene/TIMD4 for protein in rodents; TIMD4 for gene/mRNA/TIMD4 for protein in humans. They should also use accepted gene names/symbols (Stk11, Cap39, Strada/b)

TIMD4 has been proposed as a marker for long-lived resident intestinal macrophages in mice (see also PMID: 29789388) but immunolocalisation has been unconvincing and Timd4 mRNA is very low in bulk isolated intestinal macrophages (PMID: 33031383) or in total mRNA in mouse or human colon. The highest expression is in spleen, lymph node, bone marrow and liver (where TIMD4 is a Kupffer cell marker).

The rationale for this study is based upon the claim that TIMD4 is over-expressed in inflamed sites in ulcerative colitis. The IF images and quantitation in Figure 1A based upon the Santacruz antibody are unconvincing. The staining in the inflamed colon does not look cellular; colocalisation of a macrophage marker is needed and validation that the antibody binds TIMD4 exclusively on a Western blot. The claim is supported by analysis of an older NCBI-GEO microarray data which shows relative rather than absolute expression. Notably, GSE47905 appears unrelated. The author should analyse data in PMID: 34450030 (bulk RNA-seq data GSE150961, single-cell RNA-seq data GSE150516). As far as I can see TIMD4 was not identified as a disease-associated gene. The evidence that TIMD4 is increased in mouse DSS colitis Fig 1D, E, F, G is also unconvincing; the IHC quantification method is not described and the IHC result appears incompatible with the Western blot data. In any case the model makes no sense. A role for TIMD4 as a sensor of apoptotic cells does not require that it be over-expressed.

Methods state that "C57BL/6J wild-type (WT) mice were obtained from Vital River Laboratory Animal Technology. Timd4 knockout (KO) mice were provided by RIKEN BioResource Research Center (RBRC04895) [39]. Female mice (6-8 weeks old) were randomly divided into experimental and control groups".

This is insufficient information. Ref [39] is not the original reference to the knockout. Precise genetic background and possible role of intestinal microbiome (PMID: 31004013) need to be considered. The comparison between WT and KO in a colitis model is really only valid if the WT controls are litter mates of the Timd4ko mice. It is not clear why the DSS colitis model was examined only in juvenile (6-8 week) female mice. There is a substantial literature on male-female differences in the DSS colitis model. The apparent mild exacerbation in the Timd4ko mice could well be female-specific. Since the disease is more common and more severe in males this needs to be examined.

The authors attribute the impact of the Timd4ko on the DSS model to cell autonomous differences in recruited monocytes. The DSS model involves a massive granulocytosis and neutrophil infiltration. Given the major sites of expression of Timd4 in spleen, marrow and liver, indirect effects on granulocytes are equally plausible. What is the effect of the KO on blood cell homeostasis?

There is no detectable expression of Timd4 mRNAin mouse bone marrow-derived macrophages in published data (see e.g. data on BioGPS.org, also PMID: 34134993). This being the case, it is not clear why BMDM from Timd4ko mice would be different from WT. The authors appear to demonstrate TIMD4 protein in BMDM and its loss in the KO in Figure S8. There is insufficient detail provided on how the BMDM were generated. What cell concentration, substratum, where they harvested and replated? If so, how? TIMD4 is expressed by resident macrophage in bone marrow. I wonder if the authors have maintained these cells in their cultures. In any case, they need to show surface TIMD4 on their BMDM by FACS.

There is a massive jump of logic with minimal data support to invoke the STK11/CAP39/STRAD pathway in TIMD4 signalling. It seems to rely on the data in Fig S8 apparently showing the effect of AMPKinase inhibitor and LKB1(STK11) knockdown. There is no indication provided of the reproducibility of the Western blots in this Figure. It is well known that LPS promotes dephosphorylation of AMPKinase and inhibitors increase the LPS response. It is not clear why they show only the LPS-stimulated state in the Western blots. There is no dose response, no control for the knockdown. The fact that the effects of the inhibitor of AMPkinase are not additive with the apparent effect of the Timd4KO does not necessarily imply that Timd4 acts via AMPKinase. Subsequent analysis in Figure 5 relies almost entirely on over-expression and co-IP localisation in HEK293 cells. There is no compelling evidence that this pathway is engaged in macrophages.

A second jump in logic proposes "synergistic" effects of a TIMD4 peptide and Vedolizumab. The latter is a humanised antibody against a4/b7 integrin. The source of the antibody is not provided, there is no indication of a rationale for the dose, no control human Ig, and no evidence that the antibody reacts with mouse (which it does not PMID: 33847343.

Descriptions of methods and the figure legends our uniformly entirely inadequate. To cite just three examples:

Figure 3 includes an analysis of RNA-seq data focussed on chemokine gene expression. ). The full extent of the legend is: B, C) Resident macrophages from WT

and Timd4 KO mice were collected and subjected to RNA sequencing. B) Heat map analysis of chemokines. C) GSEA analysis of RNA-seq data.

The methods state that: Mouse tissue-resident macrophages were stimulated with LPS (100 ng/mL) for 3 h. RNA-seq was carried out by the Beijing Genomics Institute.

Precisely what cells, how purified, how sequenced, how quantified, full dataset…….

Figure 5 shows a large series of Western blots. How often replicated? Quantified? Apparent the final panel, labelled: (L) PEMs from WT vs. Timd4 KO mice, supports the statement (line 311) that "as predicted, Timd4 promoted membrane localization of LKB1 (Fig. 5J-L). Why suddenly look at peritoneal Macs (PEMs) (which do express TIMD4) rather than BMDM? How were PEM isolated? Does the KO alter PEM phenotype? How are the two images different? Quantification?

For Figure S7, text states that "In addition, Timd4 deficiency also reduced the proportion of M2-type macrophages with tissue-repair functions (Fig. S7), which was consistent with that Timd4 promotes macrophages polarized to be M2 phenotype [21]. Figure S7 actually shows "Representative flow cytometric plots of CD206+ cells gated on CD45+CD11b+CX3CR1+ cells". Where did the cells come from? What is the rationale for the gating? Where is it shown? What tissue repair functions are demonstrated? In any case, the M2 concept is not widely accepted and CD206 is expressed by subsets of resident tissue macrophages.

-----------------------------------------

Reviewer #2:

In this manuscript Liu et al present convincing evidence showing that Timd-signaling can ameliorate colitis in the DSS colitis model by reducing the traffic of macrophages to the gut. As such, it enhances the effect of concomitant Vedolizumab administration, another cell traffic inhibitor. However, the data presented stops well short of proving that such signaling would be effect in human disease. Thus, as mentioned at greater length in the specific comments, it is not clearly proven that this treatment approach would be effective in more chronic inflammation than is captured by the acute DSS colitis model since the latter are driven by dendritic cell function that is not inhibited by Timd signaling and signaling pathways that counter Timd signaling activity.

Specific Comments

1. Figure 1A: Timd4 staining of UC tissue is not identified as to location. Is it mainly in the epithelial layer or in the lamina propria? Localization of staining in DSS colitis in Figs N, R-S is likewise unclear.

2. DSS colitis data in Figs 1 and 2 are somewhat different: difference in WT vs KO is not significant at 6 days in Figure 1 but is significant at this time point in Figure 2. In general, differences between WT and KO are not very impressive. Were differences seen at a lower DSS dose (such as 2% DSS)? It may be that at high doses of DSS, such as the 3% dose used, one is seeing an inordinately great effect of loss of TIMd4 signaling.

3. Deletion of macrophages led to clearly higher histology scores in both WT and KO mice. Also, deletion in WT leads to greater weight loss. Macrophages seem to have a protective effect in the presence and absence of TIMd4 signaling. This anti-inflammatory effect of macrophages that is undermined by Timd signaling may be more important in more chronic models of gut inflammation that are more like human IBD.

4. Reversal of the effect of loss of Timd4 signaling in KO mice by administration of an CCR2 antagonist is impressive. If this is the case, wouldn't administration of this antagonist be a more feasible treatment of colitis than IR administration of a Timd4 surrogate by nanoparticles?

5. If Tim4d signaling is inducing M2 polarization of macrophages this should lead to increased IL-10 secretion in DSS colitis. Is this observed?

6. It is known that MAPK activation inhibits AMPK. This raises the possibility that in more chronic inflammation than the DSS colitis studied here but more like human UC, MAPK activation would negate the Timd signaling effect? Timd signaling effects need to be tested in a more chronic model of gut inflammation to substantiate its clinical value.

7. Treatment of DSS colitis by IR administration of Timd peptide-expressing NPs alone is not more effective than Vedo alone. However, it acts in synergy with Vedo. Does it enhance the effect of somewhat more powerful agents such as anti-TNFa or anti-IL-12p40? Does it enhance Vedo when both Vedo and peptide are initiated 4 days after DSS colitis is initiated. How long does the effect of Timd signaling last? Does it ameliorate DSS colitis when administered several day prior to DSS administration?

# Author comment: Author response to Decision Letter 2

**DOI:** 10.1371/journal.pbio.3004056.r003

23 Jul 2026

Attachment Submitted filename: Response to Reviewers.docx

# Peer review: Decision Letter 2

**DOI:** 10.1371/journal.pbio.3004056.r004

**Authors:** Melissa Vazquez Hernandez

9 Sep 2026

Dear Dr Gao,

Thank you for your patience while we considered your revised manuscript "TIMD4 Peptide-Nanoparticle Targets Macrophages to Ameliorate Colitis via MO25-STRAD-LKB1 Complex Stabilization and Attenuated Monocyte Recruitment" for publication as a Research Article at PLOS Biology. This revised version of your manuscript has been evaluated by the PLOS Biology editors, the Academic Editor, one of the original reviewers and a new reviewer.

Based on the reviews, we are likely to accept this manuscript for publication, provided you satisfactorily address the remaining points raised by the reviewers, specially Reviewer 3, who raises concerns about the strength of the mechanistic conclusions as the evidence that TIMD4-deficient macrophages are the main drivers of aggravated colitis is indirect, macrophage depletion does not improve disease in TIMD4-KO mice, and the evidence identifying CCL2/CCR2 signaling as the principal pathogenic mechanism is limited. While we do not request additional experiments, we do request that you tone down your statements as they are currently overstating the data presented, probably due to the way the study is written. The conclusions are presented very definitively, sometimes without the relevant context needed to judge whether they are definitively supported, or in what contexts. Therefore, we request that you edit the results and discussion to be clearer about the nature of the evidence underlying the claims, following Reviewer 3's comments. Some examples regarding this issue can also be:

*on line 358 you say: "In summary, these findings demonstrate that TIMD4 promotes the assembly of the LKB1 complex through its intracellular domain, enhancing LKB1 membrane localization and phosphorylation, and potentiating AMPK activation. This cascade suppresses NF‑κB‑driven chemokine production..."

^It is not clear in what system does this happen. While those details are present in the preceding paragraphs, this statement makes it seems like this is a universal mechanism in all cells.

*The abstract should specify further in what model systems the data come from. Which could also be extended to the discussion. This would help overcome concerns about overstating claims. Ex: is this data from in vivo or in vitro studies?: "Mechanistically, TIMD4 stabilized the MO25-STRAD-LKB1 complex via its intracellular domain, thereby activating the AMPK pathway and suppressing NF-κBdependent chemokine production in macrophages". If so you might add 'Mechanistically, in vitro studies reveal that ..."

ADDITIONALLY, please also make sure to address the following data and other policy-related requests.

1) We routinely suggest changes to titles to ensure maximum accessibility for a broad, non-specialist readership, and to ensure they reflect the contents of the paper. Please ensure you change both the manuscript file and the online submission system, as they need to match for final acceptance:

"A macrophage-targeted TIMD4 Peptide-Nanoparticle attenuates colitis symptoms and monocyte recruitment in a mouse model"

2) Please add weblink of the funding agencies in the Financial Disclosure statement in the manuscript details.

3) The Ethics statement needs to be a separate, independent (and the first) subheading in the Material & Methods section. It must include the full name of the IACUC/ethics committee that reviewed and approved the animal care and use, as well as the protocol/permit/project license number. https://journals.plos.org/plosbiology/s/ethical-publishing-practice

4) We do not have a word limit. Please move the supplementary materials and methods to the main text which can provide the readers an easier access to all information.

5) Thank you for providing the underlying data for all figures. I believe we might be missing the data for Figures 5D and 6D. Additionally, the data for 1X and 1Y might be mislabeled.

6) Please cite the location of the data clearly in all relevant main and supplementary Figure legends, e.g. “The data underlying this Figure can be found in S1 Data” or “The data underlying this Figure can be found in https://doi.org/10.5281/zenodo.XXXXX”

7) For figures containing FACS data (Figures 2BI, 3HJ, 4G, S6, S9A), please provide the FCS files and a picture showing the successive plots and gates that were applied to the FCS files to generate the figure. We ask that you please deposit this data to an open access repository like Zenodo and provide the accession number/URL of the deposition in the Data Availability Statement in the online submission form. You can also upload the files in the system.

8) Please ensure that your Data Statement in the submission system accurately describes where your data can be found and is in final format, as it will be published as written there

9) Per journal policy, if you have generated any custom code during the course of this investigation, please make it available without restrictions. Please ensure that the code is sufficiently well documented and reusable, and that your Data Statement in the Editorial Manager submission system accurately describes where your code can be found. More information on our Code Policy, what and how to share can be found here: https://journals.plos.org/plosbiology/s/code-availability

Please note that we cannot accept sole deposition of code in GitHub, as this could be changed after publication. However, you can archive this version of your publicly available GitHub code to Zenodo. Once you do this, it will generate a DOI number, which you will need to provide in the Data Accessibility Statement (you are welcome to also provide the GitHub access information). See the process for doing this here: https://docs.github.com/en/repositories/archiving-a-github-repository/referencing-and-citing-content

As you address these items, please take this last chance to review your reference list to ensure that it is complete and correct. If you have cited papers that have been retracted, please include the rationale for doing so in the manuscript text, or remove these references and replace them with relevant current references. Any changes to the reference list should be mentioned in the cover letter that accompanies your revised manuscript.

In addition to these revisions, you may need to complete some formatting changes, which you will receive in a follow up email. A member of our team will be in touch with a set of requests shortly. If you do not receive a separate email within a few days, please assume that checks have been completed, and no additional changes are required.

We expect to receive your revised manuscript within two weeks.

To submit your revision, please go to https://www.editorialmanager.com/pbiology/ and log in as an Author. Click the link labelled 'Submissions Needing Revision' to find your submission record. Your revised submission must include the following:

- a cover letter that should detail your responses to any editorial requests, if applicable, and whether changes have been made to the reference list

- a Response to Reviewers file that provides a detailed response to the reviewers' comments (if applicable, if not applicable please do not delete your existing 'Response to Reviewers' file.)

- a track-changes file indicating any changes that you have made to the manuscript.

NOTE: If Supporting Information files are included with your article, note that these are not copyedited and will be published as they are submitted. Please ensure that these files are legible and of high quality (at least 300 dpi) in an easily accessible file format. For this reason, please be aware that any references listed in an SI file will not be indexed. For more information, see our Supporting Information guidelines:

https://journals.plos.org/plosbiology/s/supporting-information

*Published Peer Review History*

Please note that you may have the opportunity to make the peer review history publicly available. The record will include editor decision letters (with reviews) and your responses to reviewer comments. If eligible, we will contact you to opt in or out. Please see here for more details:

https://plos.org/published-peer-review-history/

*Press*

Should you, your institution's press office or the journal office choose to press release your paper, please ensure you have opted out of Early Article Posting on the submission form. We ask that you notify us as soon as possible if you or your institution is planning to press release the article.

*Protocols deposition*

To enhance the reproducibility of your results, we recommend that if applicable you deposit your laboratory protocols in protocols.io, where a protocol can be assigned its own identifier (DOI) such that it can be cited independently in the future. Additionally, PLOS ONE offers an option for publishing peer-reviewed Lab Protocol articles, which describe protocols hosted on protocols.io. Read more information on sharing protocols at https://plos.org/protocols?utm_medium=editorial-email&utm_source=authorletters&utm_campaign=protocols

Please do not hesitate to contact me should you have any questions.

Sincerely,

Melissa

Melissa Vazquez Hernandez, Ph.D.

Senior Editor

mvazquezhernandez@plos.org

PLOS Biology

------------------------------------------------------------------------

REVIEWERS' COMMENTS

------------------------------------------------------------------------

Reviewer #2:

THe authors have provided positive response to each of the comments of the 2nd review including the comment of both reviewers. In many cases this included extensive new data.

------------------------------------------------------------------------

Reviewer #3:

The authors show that TIMD4 has a protective role in DSS colitis. They claim that the underlying mechanism is the increased production of cytokines and chemokines (especially CCL2) by Timd4-deficient macrophages. However, they provide only indirect evidence e.g. by treating WT and Timd4-KO mice with clodronate liposomes or a CCR2 antagonist. If Timd4-deficient macrophages would be the main disease drivers, then depletion of macrophages with clodronate should improve colitis in Timd4-KO mice, which is not the case. Of course, one could argue that macrophages also have a protective role, independent of their expression of Timd4 and then the depletion of these macrophages would not improve colitis even in Timd4-KO mice. Unfortunately, no data in this direction are provided.

The evidence that CCL2 is the main disease driver in Timd4-KO mice is also very thin and indirect. Just the fact that treatment with an CCR2-antagonst induces somewhat more improvement in Timd4-KO mice than in WT mice, does not mean very much.

There a lot of other potential mechanisms how TimD4 deficiency could exacerbate colitis, e.g. due to the accumulation of apoptotic cells (see Fig. 1 R, S) or missing interaction of TIMD4 with T cells (PMID: 18367551).

Nevertheless, I would not be overly critical in this regard, as the authors provide at least some potential mechanisms. However, I would request that they modify their rather strong statements (e.g. line 482 ff "In the present study, we demonstrate that TIMD4 exerts a protective effect against IBD by precisely modulating controls the production of Ccl2 and additional chemokines via the LKB1/AMPK/NF-κB signaling axis, which effectively curtails inhibits inflammatory monocyte infiltration and prevents local immune overactivation, thus exerting a protective effect against IBD.")

The protective effects of nanoparticles coated with a Timd4 peptide T4(365-378)-NPs, remains a bit enigmatic to me. After uptake of nanoparticles by macrophages, how should the nanoparticles get access to the intracellular space to induce signal transduction in macrophages? Would be great to see data (not only for p-P65 like in Fig. 6G, H, but also more relevant read-outs). E.g. does treatment with nanoparticles reduce the LPS-induced expression of CCL2 and other cytokines / chemokines. However, I would not consider this as a mandatory point.

Regarding the reply to the comments of reviewer 1, I feel that these are acceptable.

Overall I think that the paper is suitable for publication. Some minor points are listed below.

Minor points:

1. Line 200: Fig. 1 H-K instead of Fig. 1 D-G

2. Line 206: Please write: increased number of apoptotic cells instead of "increased apoptosis". Most likely apoptotic cells are less efficiently cleared in Timd4 KO mice. Fig 1R-S instead of Fig. 1S

3. Line 211: In addition instead of "Meanwhile"

4. Unclear if treatment with T4(365-378)-NPs in combination with an CCR2-anatgonist, anti-α4β7 or anti-TNFα was done in WT or TIMD4-KO mice.

# Author comment: Author response to Decision Letter 3

**DOI:** 10.1371/journal.pbio.3004056.r005

23 Sep 2026

Attachment Submitted filename: Response to Reviewer .docx

# Editor report: Decision Letter 3

**DOI:** 10.1371/journal.pbio.3004056.r006

**Authors:** Melissa Vazquez Hernandez

29 Sep 2026

Dear Dr Gao,

Thank you for the submission of your revised Research Article "A macrophage-targeted TIMD4 Peptide-Nanoparticle attenuates colitis symptoms and monocyte recruitment in a mouse model" for publication in PLOS Biology. On behalf of my colleagues and the Academic Editor, Ken Cadwell, I am pleased to say that we can in principle accept your manuscript for publication, provided you address any remaining formatting and reporting issues. These will be detailed in an email you should receive within 2-3 business days from our colleagues in the journal operations team; no action is required from you until then. Please note that we will not be able to formally accept your manuscript and schedule it for publication until you have completed any requested changes.

Please take a minute to log into Editorial Manager at http://www.editorialmanager.com/pbiology/, click the "Update My Information" link at the top of the page, and update your user information to ensure an efficient production process.

PRESS

We frequently collaborate with press offices. If your institution or institutions have a press office, please notify them about your upcoming paper at this point, to enable them to help maximise its impact. If the press office is planning to promote your findings, we would be grateful if they could coordinate with biologypress@plos.org. If you have previously opted in to the early version process, we ask that you notify us immediately of any press plans so that we may opt out on your behalf.

We also ask that you take this opportunity to read our Embargo Policy regarding the discussion, promotion and media coverage of work that is yet to be published by PLOS. As your manuscript is not yet published, it is bound by the conditions of our Embargo Policy. Please be aware that this policy is in place both to ensure that any press coverage of your article is fully substantiated and to provide a direct link between such coverage and the published work. For full details of our Embargo Policy, please visit http://www.plos.org/about/media-inquiries/embargo-policy/.

Thank you again for choosing PLOS Biology for publication and supporting Open Access publishing. We look forward to publishing your study.

Sincerely,

Melissa

Melissa Vazquez Hernandez, Ph.D., Ph.D.

Senior Editor

PLOS Biology

mvazquezhernandez@plos.org
